# Dense genotyping-by-sequencing linkage maps of two Synthetic W7984×Opata reference populations provide insights into wheat structural diversity

**DOI:** 10.1038/s41598-018-38111-3

**Published:** 2019-02-11

**Authors:** Juan J. Gutierrez-Gonzalez, Martin Mascher, Jesse Poland, Gary J. Muehlbauer

**Affiliations:** 1 Department of Agronomy and Plant Genetics, University of Minnesota, Saint Paul, MN 55108 USA; 2 Leibniz Institute of Plant Genetics and Crop Plant Research (IPK), D-06466 Seeland OT, Gatersleben Germany; 3 German Centre for Integrative Biodiversity Research (iDiv) Halle-Jena-Leipzig, Deutscher Platz 5e, 04103 Leipzig, Germany; 4 Wheat Genetics Resource Center, Department of Plant Pathology, Kansas State University, 4024 Throckmorton Plant Sciences Center, Manhattan, KS 66506 USA; 5 Department of Plant and Microbial Biology, University of Minnesota, Saint Paul, MN 55108 USA

## Abstract

Wheat (*Triticum aestivum*) genetic maps are a key enabling tool for genetic studies. We used genotyping-by-sequencing-(GBS) derived markers to map recombinant inbred line (RIL) and doubled haploid (DH) populations from crosses of W7984 by Opata, and used the maps to explore features of recombination control. The RIL and DH populations, SynOpRIL and SynOpDH, were composed of 906 and 92 individuals, respectively. Two high-density genetic linkage framework maps were constructed of 2,842 and 2,961 cM, harboring 3,634 and 6,580 markers, respectively. Using imputation, we added 43,013 and 86,042 markers to the SynOpRIL and SynOpDH maps. We observed preferential recombination in telomeric regions and reduced recombination in pericentromeric regions. Recombination rates varied between subgenomes, with the D genomes of the two populations exhibiting the highest recombination rates of 0.26–0.27 cM/Mb. QTL mapping identified two additive and three epistatic loci associated with crossover number. Additionally, we used published POPSEQ data from SynOpDH to explore the structural variation in W7984 and Opata. We found that chromosome 5AS is missing from W7984. We also found 2,332 variations larger than 100 kb. Structural variants were more abundant in distal regions, and overlapped 9,196 genes. The two maps provide a resource for trait mapping and genomic-assisted breeding.

## Introduction

Modern cultivated wheat is an allohexaploid (2n = 6×  = 42) that derives from two hybridizations of three ancestral diploid genomes (A, B, and D). The first hybridization occurred around 0.5 MYA to form the allotetraploid emmer wheat (*T*. *turgidum* L., AABB)^1^. The second, a more recent hybridization, took place around 8,000 years ago between emmer wheat and goat grass (*Aegilops tauschii* Coss.), the progenitor of the wheat D genome, and led to the formation of hexaploid wheat^2^. Because of the recent hybridization and the reduced number of hybridization events between *T*. *turgidum* and *Ae*. *taushii*, the wheat D genome has a narrow genetic base^3^. Yet, *Ae*. *tauschii* is a source of economically important traits such as yield, disease resistance, bread-making quality, and suppression of pre-harvest sprouting^4^. Present-day relatives of ancient emmer wheat and goat grass are recognized as important sources of variation that merit exploring^3,5^. For instance, tetraploid emmer relatives carry beneficial alleles for grain quality traits, abiotic factors (drought, heat, salinity, and waterlogging), and resistance to several rusts, viruses, fungi and nematodes^6^. Nevertheless, despite the potential, genetic diversity derived from *T*. *turgidum* and *Ae*. *tauschii* is not well represented in modern bread wheat germplasm. The observation that both *Ae*. *tauschii* and *T*. *turgidum* retain a higher genetic diversity compared to wheat cultivars and landraces has inspired the modern recreation of synthetic hexaploid wheat by mimicking the process that spontaneously arose in nature.

Genetic studies and breeding benefit from the availability of mapping populations. Two of the most widely used wheat populations were developed from the cross of a synthetic wheat (Synthetic W7984, herein abbreviated ‘W7984’) and Opata M85 (‘Opata’). The original recombinant inbred line (RIL) population W7984 × Opata was developed in the early 90 s as a cross between a synthetic hexaploid wheat, generated by crossing a *Ae*. *tauschii* (DD) accession with the durum wheat ‘Altar 84’ (AABB), and the spring wheat cultivar Opata 85^7^. This reference population is known as ‘SynOpRIL’, and was later reconstructed and the number of individuals increased to 2,039^8^. In parallel, a doubled haploid (DH) population of 215 individuals was developed using the same parental accessions and designated the ‘SynOpDH’ population. With the availability of a reference genome sequence, high-density anchored genetic maps will greatly enhance the utility of the SynOpRIL and SynOPDH populations.

Recent advances in sequencing technologies, assembly pipelines and algorithms have allowed tackling highly repetitive polyploid genomes^5,9–11^. The recent publication of an almost complete, fully annotated, anchored and ordered reference genome assembly of wheat, the Chinese Spring RefSeq v1.0 (hereafter RefSeq v1.0) has boosted wheat genomic studies in an unprecedented manner^10^. Integration of this reference genome with genetic map resources will enable greater utility for studies needing genetic positions such as genomic evaluation of *in silico* breeding progeny.

Marker-assisted selection and genomic selection necessitate a set of high-density markers for accuracy. Moreover, a prerequisite to build an accurate and comprehensive genetic map suitable for QTL positioning is a set of reliable markers evenly distributed across the genome^12^. Genotyping-by-sequencing (GBS) is a high-density genotyping approach extensively used in breeding and genetics because of its low cost, high number and uniform distribution of markers, and the capacity to simultaneously perform polymorphism discovery and genotyping^13,14^. However, to reduce costs, a large number of samples are pooled and sequenced together. Thus, although GBS is able to produce a substantial number of markers, keeping per-sample cost down requires multiplexing of the samples, which in turn lowers the sequencing depth and creates a substantial rate of missing observations^14^. Imputation of genotypes frequently follows GBS to estimate missing observations and thus to reduce the proportion of missing data contained across samples.

Here, we used a subset of both SynOpRIL and SynOpDH populations to (i) construct two highly-dense framework genetic maps and populate them with imputed markers; (ii) study recombination rates and segregation distorted regions; (iii) identify loci that affect the number of recombination events; and (iv) examine the genomes of W7984 and Opata for structural variations. Altogether, our findings provide a rich resource for marker-assisted selection, QTL mapping and genomic-assisted breeding.

## Results

### High-density linkage map construction

**Figure 1:**
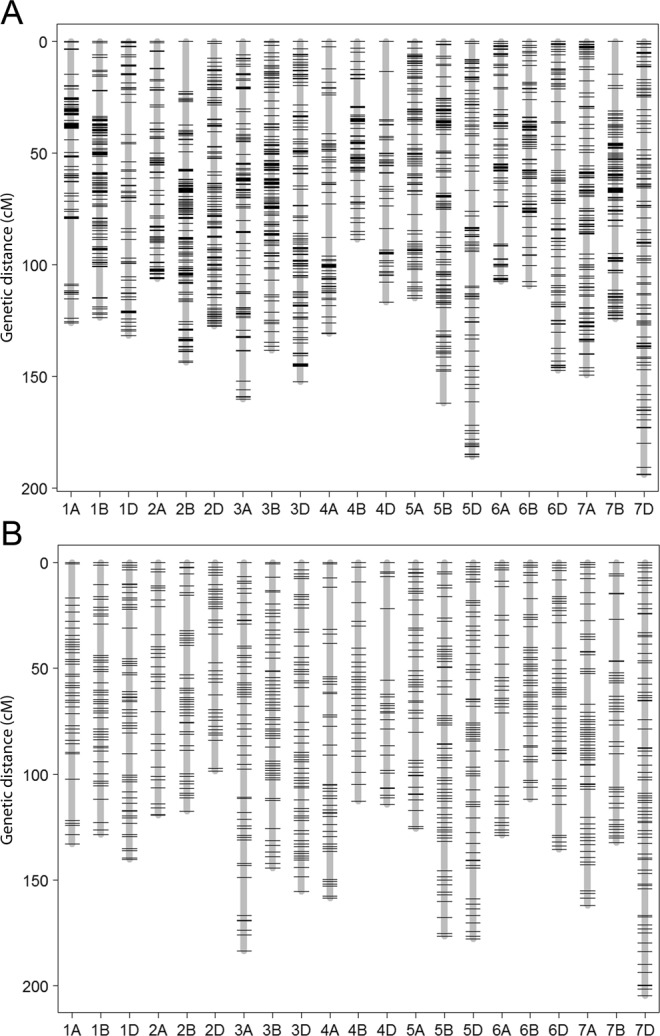
Genetic linkage maps constructed for the (**A**) SynOpRIL and (**B**) SynOpDH reference populations derived from the cross W7984 × Opata. Linkage groups corresponding to the 21 wheat chromosomes are shown in x-axis. The y-axis shows genomic distances in cM.

We aimed to develop a new set of GBS-SNP markers under strict quality controls and extensive manual curation. The high-quality GBS markers were used as the foundation for constructing two accurate framework maps using the two reference populations SynOpRIL and SynOpDH. In the SynOpRIL population, we genotyped 1,100 individuals plus the parental lines. After removing lines based on high rate of missing or heterozygous observations (161), high similarity (27), and a high number of double crossovers (6), 906 met the threshold and were used to create the genetic map (Supplementary Data S1). Stringent filtering removed low-quality markers and left 5,931 markers as input for linkage analysis. A genetic linkage map, termed *SynOpRIL906*, was constructed using 3,634 of those high quality GBS-SNPs giving an average rate of missing allele calls of the mapped framework markers at 3.2%. We built a complete framework map distributed into 1,956 unique genetic bins (Fig. 1A and Supplementary Data S2) that covered 2,842 cM. Individual chromosomes averaged 135 cM long, ranging from 89 cM (chromosome 4B) to 194 cM (chromosome 7D), and the number of SNPs per chromosome was significantly correlated with the physical size of the chromosome (Pearson’s correlation ρ_p_ = 0.72, p = 0.0002). The average bin interval size (cM/num. of bins) of the map was 1.45 cM, ranging from 1.07 cM (2B) to 2.65 cM (4D) (Table 1). The B genome had the largest number of markers, 1,489 (40.97%), followed by A with 1,177 (32.39%) and D (968, 26.64%). The largest gap was located on chromosome 1A with 29.3 cM. There were only two more gaps larger than 20 cM.

**Table 1:** Summary statistics of genetic maps of *SynOpRIL906* and *SynOpDH92*.

Chr	SynOpRIL906							SynOpDH92						
	no. of markers	Markers (%)	Bins	Length (cM)	ABI^a^ (cM)	Marker/cM^b^	cM/Mb	no. of markers	Markers (%)	Bins	Length (cM)	ABI^a^ (cM)	Marker/cM^b^	cM/Mb
1A	154	4.24	81	126.2	1.56	1.22	0.21	263	4.00	41	132.8	3.24	1.98	0.23
1B	215	5.92	113	123.6	1.09	1.74	0.18	416	6.32	46	128.4	2.79	3.24	0.19
1D	90	2.48	63	131.7	2.09	0.68	0.27	188	2.86	52	140.1	2.69	1.34	0.28
2A	162	4.46	74	106.2	1.44	1.53	0.14	186	2.83	30	119.3	3.98	1.56	0.16
2B	250	6.88	134	143.8	1.07	1.74	0.18	418	6.35	42	117.6	2.80	3.55	0.15
2D	181	4.98	113	127.7	1.13	1.42	0.20	176	2.67	36	98.6	2.74	1.78	0.15
3A	184	5.06	99	160.1	1.62	1.15	0.21	357	5.43	52	183.6	3.53	1.94	0.24
3B	242	6.66	125	138.3	1.11	1.75	0.17	460	6.99	56	144.4	2.58	3.19	0.17
3D	196	5.39	108	152.6	1.41	1.28	0.25	334	5.08	58	155.2	2.68	2.15	0.25
4A	118	3.25	63	130.9	2.08	0.90	0.18	312	4.74	43	158.6	3.69	1.97	0.21
4B	152	4.18	67	88.8	1.33	1.71	0.13	273	4.15	29	112.7	3.89	2.42	0.17
4D	67	1.84	44	116.8	2.65	0.57	0.23	109	1.66	24	114.4	4.77	0.95	0.23
5A	159	4.38	87	114.9	1.32	1.38	0.29	244	3.71	43	125.8	2.93	1.94	0.28
5B	201	5.53	121	162.1	1.34	1.24	0.23	351	5.33	63	176.6	2.80	1.99	0.25
5D	154	4.24	89	186.2	2.09	0.83	0.33	278	4.22	62	177.8	2.87	1.56	0.31
6A	145	3.99	79	107.5	1.36	1.35	0.17	263	4.00	32	128.8	4.02	2.04	0.21
6B	164	4.51	81	109.5	1.35	1.50	0.15	356	5.41	46	111.7	2.43	3.19	0.16
6D	119	3.27	82	147.4	1.80	0.81	0.31	218	3.31	48	135.4	2.82	1.61	0.29
7A	255	7.02	115	149.5	1.30	1.71	0.20	502	7.63	60	162.0	2.70	3.10	0.22
7B	265	7.29	113	124.4	1.10	2.13	0.17	478	7.26	38	132.5	3.49	3.61	0.18
7D	161	4.43	105	194.0	1.85	0.83	0.31	398	6.05	72	204.6	2.84	1.95	0.32
A genome	1177	32.39	598	895.3	1.50	1.31	0.19	2127	32.33	301	1011.0	3.36	2.10	0.22
B genome	1489	40.97	754	890.4	1.18	1.67	0.17	2752	41.82	320	923.8	2.89	2.98	0.18
D genome	968	26.64	604	1056.3	1.75	0.92	0.27	1701	25.85	352	1026.1	2.92	1.66	0.26
Total	3634		1956	2842.1	1.45	1.28	0.21	6580		973	2961.0	3.04	2.22	0.22

^a^ABI: Average bin interval size.

^b^Marker density: number of markers per cM.

For the SynOpDH reference population, a total of 177 double haploids plus the parents were genotyped, of which 92 passed quality controls and were used. DHs that fail to pass quality controls were due to very low sequencing depth (39), high rate of missing or heterozygous observations (43), or excess of double recombinants (3) (Supplementary Data S1). A total of 18,822 GBS-SNP markers passed the initial quality filters. After removing markers with excess heterozygosity and missing observations, and testing for linkage, a final number of 6,580 GBS markers were grouped into 21 linkage groups and incorporated in the genetic map (Fig. 1B and Supplementary Data S3). The rate of missing allele observations of the mapped markers was 3.3%. The Pearson’s correlation between number of SNPs per chromosome and the chromosome size was ρ_p_ = 0.63, p = 0.0021. This map, which was termed *SynOpDH92*, was 2,961 cM and had 973 recombination bins (Table 1). Individual chromosomes averaged 141 cM in size ranging from 98 (chromosome 2D) to 204 (chromosome 7D). The average bin interval size was 3.04 cM, ranging from 2.89 cM (B genome) to 3.36 cM (A genome), and a marker density of 2.22 markers per cM. As in the *SynOpRIL906*, the B genome had the largest number of markers, 2,752 (41.82%), followed by A with 2,127 (32.33%) and D with 1,701 (25.85%). Overall, the map exhibited good contiguity, with only one gap larger than 20 cM, located on chromosome 4D, of 33.8 cM long.

**Figure 2:**
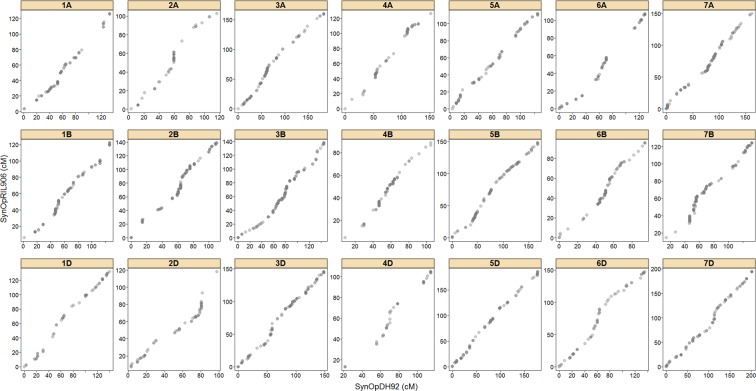
Marker order correlation between *SynOpRIL906* and *SynOpDH92*. Graph shows alignment of the 2,113 shared markers for all 21 linkage groups. Axis distances in cM.

The *SynOpRIL906* and *SynOpDH92* framework maps were subjected to several steps of quality control. First, *SynOpRIL906* and the *SynOpDH92* shared a total of 2,113 markers in common. Alignment of these shared positions in both maps showed that there was a linear relationship between the two (Fig. 2). The correlation between marker order was found to be very high (Spearman’s rank correlation coefficient ρ_s_ = 0.969, p < 2.2E-16). It is important to note that both maps lack markers on the short arm of chromosome 5A. Second, genetic positions in the *SynOpRIL906* and *SynOpDH92* maps were also checked for correlations with physical positions in the RefSeq v1.0 assembly (Supplementary Fig. S1). Spearman’s correlation between both *SynOpRIL906* and *SynOpDH92* linkage map positions and the RefSeq v1.0 positions was ρ_s_ = 0.999 p < 2.2E-16. Third, pair-wise recombination fractions were estimated and used to plot heatmaps. Recombination fraction heatmaps show strong linkage between adjacent markers and no abrupt transitions that could indicate presence of misplaced markers (Supplementary Figs S2 and S3).

### Marker Imputation

The purpose of marker imputation is to estimate unobserved genotype calls from marker datasets. To increase the number of markers on the framework maps of both populations, we imputed markers that originally did not pass quality filters for framework markers (see Materials & Methods). A hidden Markov model^15^ that has shown high accuracy in low-coverage biallelic populations generated a total of 49,096 and 99,888 imputed markers for the SynOpRIL and SynOpDH populations, respectively (Supplementary Data S4 and S5). Following imputation, the new imputed markers were added to the framework maps. Pair-wise Hamming distances were computed between imputed and framework markers and used to assign imputed markers to bins. A total number of 43,013 (87.6%) and 86,042 (86.1%) imputed markers were successfully incorporated to the *SynOpRIL906* and *SynOpDH92* maps, respectively (Supplementary Data S6 and S7). The correlation between the total number of SNPs (imputed plus framework) per chromosome and chromosome physical size was moderately high (Pearson’s ρ_p_ = 0.64 p = 0.0017; and ρ_p_ = 0.58 p = 0.0063, respectively). The statistics of the maps including added imputed markers is summarized in Supplementary Table S2.

### Allele frequencies and segregation distortion

**Figure 3:**
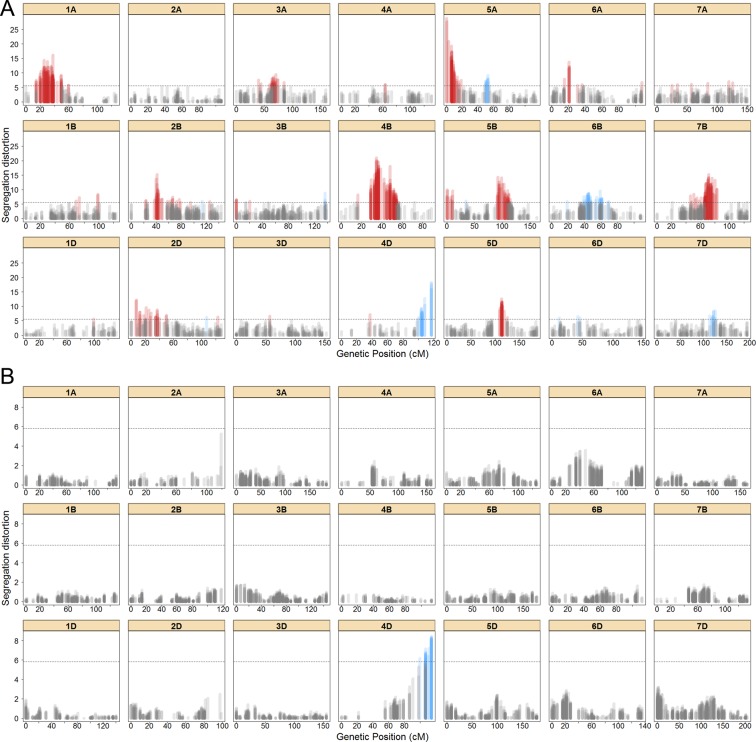
Segregation distorted regions for the *SynOpRIL906* (**A**) and *SynOpDH92* maps (**B**). The x-axis shows map distances in cM and the y-axis the −log10 p-values from tests of 1:1 segregation at each marker. Dashed horizontal lines indicate the level of significance after Bonferroni correction. Colored bars indicate that a particular marker is significantly distorted, either towards the W7984 allele (blue) or Opata allele (red).

Segregation distortion, the deviation of the segregation ratio of a locus from the expected Mendelian ratio, is often observed in mapping populations and is known to impact genetic map construction. Parental allele frequencies for all framework markers in both populations were computed (Supplementary Fig. S4), and used to assess the presence of segregation distorted markers. Overall, markers in the *SynOpRIL906* map had a higher frequency of the Opata alleles (52.2% vs. 47.8%) (Supplementary Fig. S4A). Out of the 3,634 mapped markers in the *SynOpRIL906* framework map, 572 (15.7%) showed significant distortion after Bonferroni correction for multiple testing (p < 0.01, single test), with marker alleles biased toward either parent (Fig. 3A). Among the 572 segregation distorted markers, 95 showed deviation towards W7984 alleles and 477 towards Opata alleles, with 188 (32.9%) present in the A genome, 303 (53.0%) in the B genome, and 81 (14.1%) in the D genome. All chromosomes except 2A had at least one marker displaying segregation distortion. Rather than randomly distributed, markers exhibiting segregation distortion tend to be clustered in the linkage maps (Fig. 3A). Clusters of 10 or more adjacent distorted markers were defined as segregation distorted regions (SDRs), most of which occurred in long ( > 20 cM) blocks. There were 12 SDRs across all chromosomes, four in the A genome, five in B, and three in D. Five of those SDRs had more than 40 markers. The cluster in chromosome 4B has the largest number of distorted markers (108).

Considerably less segregation distortion was observed in the *SynOpDH92* map (Fig. 3B), where there was slightly more presence of W7984 alleles (50.2%) than Opata alleles (49.8%) (Supplementary Fig. S4B). Just one SDR was highlighted as significant, in chromosome 4D. This region comprised a total of 29 (0.44%) markers exhibiting segregation distortion, all W7984 alleles.

### Recombination frequencies and recombination QTL

**Figure 4:**
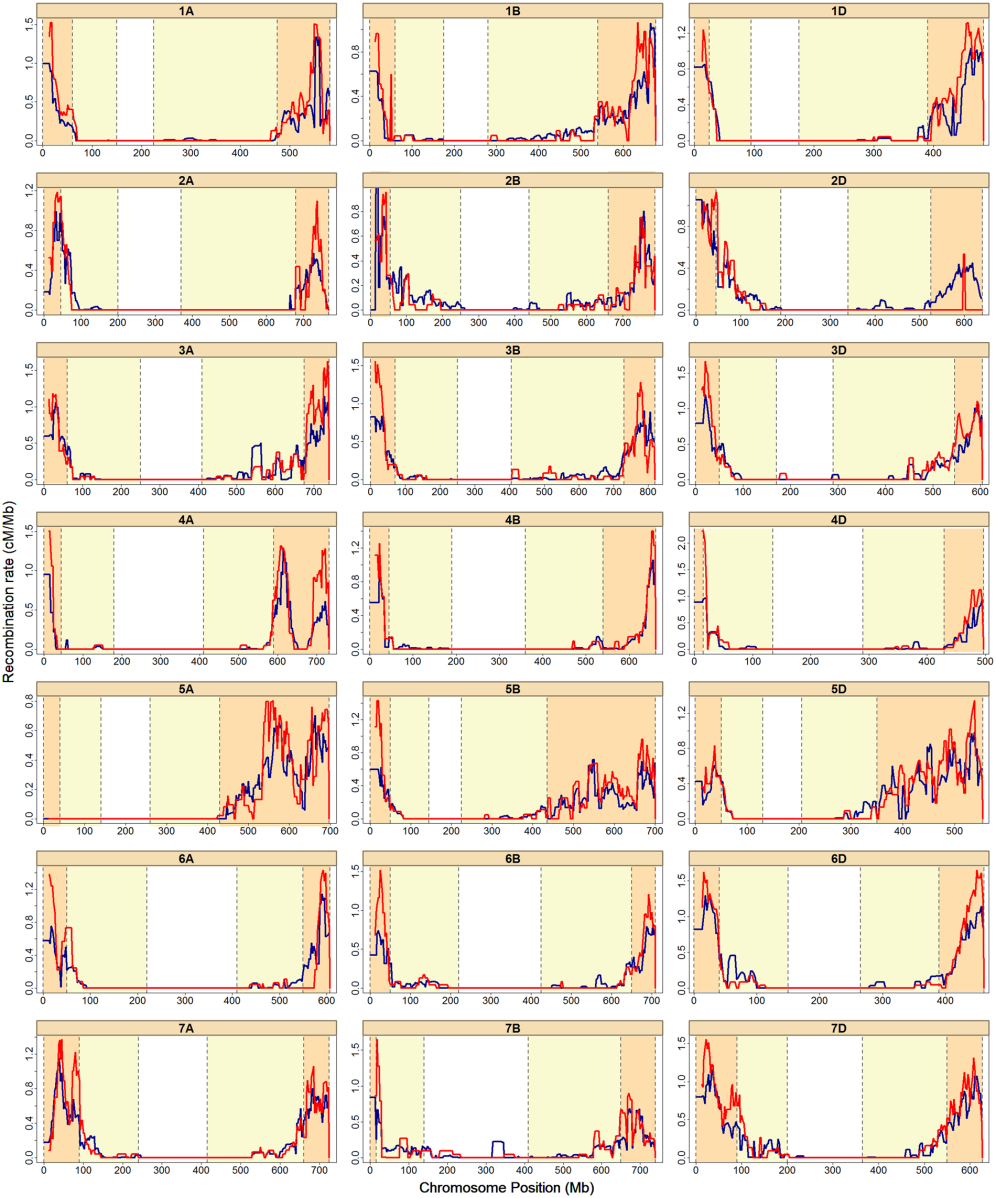
Estimates of recombination rates (cM/Mb). Distribution of recombination rates for the *SynOpRIL906* (blue) and *SynOpDH92* (red) maps across the 21 wheat chromosomes. The x-axis represents the chromosomal positions (Mb), and the y-axis recombination rates in cM/Mb. Vertical dashed lines correspond to the regions outlined in^10^, which divided each chromosome according to gene content and recombination rates into distal regions (orange), centromeric regions (white), and intermediate (yellow).

**Figure 5:**
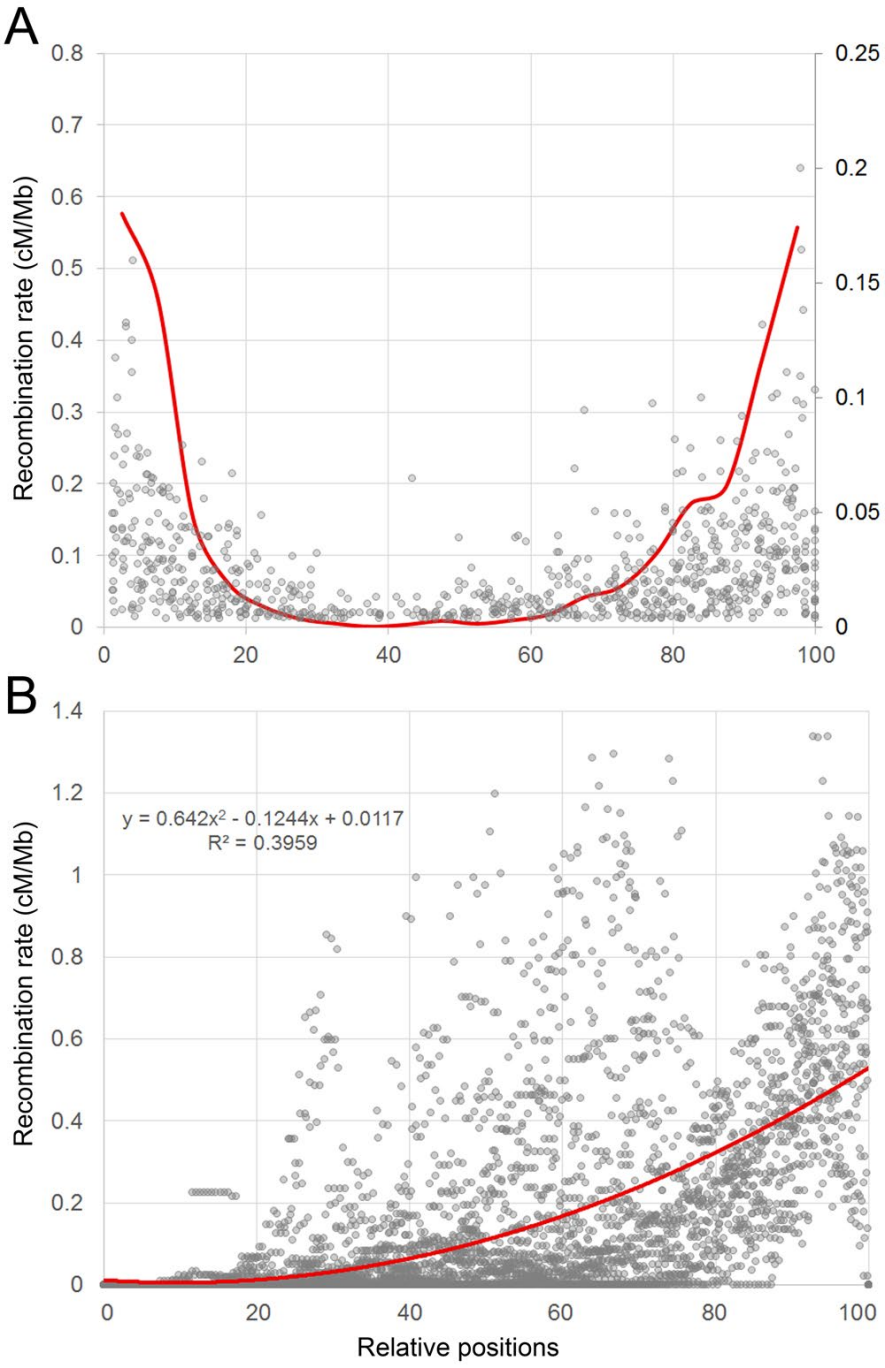
Recombination trend (cM/Mb) along wheat genome was calculated using the *SynOpRIL906* map. (**A**) Physical distances were normalized either by the relative length of each chromosome (**A**) or by the relative distance from the centromere (**B**). Dots in (**A**) represent the relative frequency of GBS-SNP markers across normalized chromosomes; while in (**B**) represent each recombination rate datapoint, with the curve and the equation that best fits the data. Centromere positions were obtained from^10^.

The recombination rate (cM/Mb) tends to vary widely across the genome. We computed recombination rates for all chromosomes using the two framework maps (Supplementary Table S3). On average, the D genome had the highest values, 0.27 and 0.26 cM/Mb for *SynOpRIL906* and *SynOpDH92*, respectively. The A genome exhibited 0.19 and 0.22, and B genome had 0.17 and 0.18 cM/Mb. The average recombination rates for all three genomes were 0.21 cM/Mb for *SynOpRIL906* and 0.22 cM/Mb for *SynOpDH92*. There was a strong negative correlation between recombination rates and the sizes of physical chromosomes (ρ_p_ = −0.62, p = 0.0025 for *SynOpRIL906*, and ρ_p_ = −0.59, p = 0.0046 for *SynOpDH92*). Recombination rates were plotted onto the RefSeq v1.0 sequence for each individual chromosome (Fig. 4) and trends in recombination along the wheat genome were examined in the *SynOpRIL906* and *SynOpDH92* maps. Chromosome physical distances were normalized either by the relative length of each chromosome (Fig. 5A and Supplementary Fig. S5A), or by the relative distance from the centromere (Fig. 5B and Supplementary Fig. S5B). Recombination rates showed a clear trend, with the highest rates towards the end of chromosomes and rates near zero around the centromere region.

In a number of populations, recombination rates have been found to be influenced by loci distributed throughout the genome^16,17^. We took advantage of the high number of individuals and the increased number of recombination events in SynOpRIL to ascertain whether there are loci that have an effect on recombination in this population. The number of crossovers (CO) per inbred line followed a normal distribution of mean 54.2, with values ranging from 26 to 101 (Supplementary Fig. S6). We used a multiple interval mapping (MIM) approach to detect QTLs. MIM fits several markers and their epistatic interactions simultaneously in a mixed model (see Materials & Methods). The observed phenotypic variation was initially fitted into a MIM model with 10 QTLs (LOD ≥ 2) and four epistatic interactions. Synthetic W7984 carried the positive alleles for eight out of 10 QTL in the model. After several iterative rounds, a stable model was found with two significant (LOD > 5.5) additive QTLs, located in chromosomes 6A (LOD = 15.1, R2 = 6.45) and 6D (LOD = 7.1, R2 = 2.97), and three significant (LOD > 12.8) epistatic interactions (Supplementary Tables S4 and S5). The QTLs in 6A and 6D were not in syntenous locations.

### Genome-wide read depth variation (RDV)

**Figure 6:**
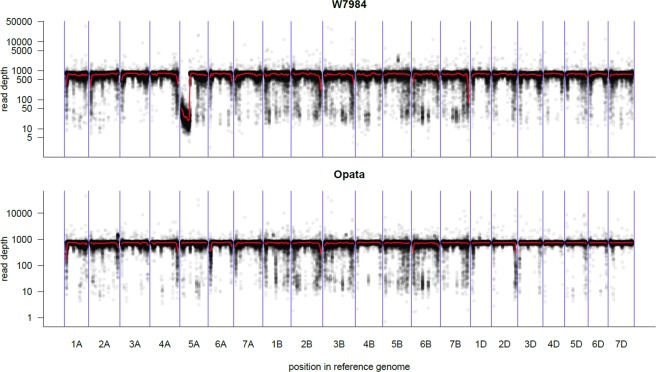
Normalized read count coverage in the W7984 and Opata along the genome. Sequenced reads were aligned to RefSeq v1.0. Mapped reads were counted in 100 kb windows and normalized (counts per 100 million reads mapped). Red line shows *Loess* regression, a non-parametric approach that fits multiple regressions in local neighborhood. Of note is the lack of reads in the short arm of chromosome 5A in W7884. The background is likely off-target mapping. The y-axis in log scale.

We benefited from the recent low depth shotgun sequencing (1.37 × genome coverage) of a subset of 90 individuals of the SynOpDH population^18^ and explored the genomes of W7984 and Opata for structural variations. Sequenced reads were aligned to RefSeq v1.0, and the mapped reads were counted in 100 kb windows and normalized (counts per 100 million reads mapped) (Fig. 6). We again noted that the whole short arm of chromosome 5A appears to be missing in the W7984 parent. To assess which of the 90 segregating DH lines also lack 5AS, first, the median normalized read counts in 100 kb bins was computed for the missing 5AS interval (Supplementary Data S8). Second, the read depth was plotted along chromosome 5A for all DH lines (Supplementary Data S9). The 5AS appears to be absent in 40 (45%) of the DHs, while it is present in 49 (55%). There is a line (SynOpDH0030) that displays intermediate values, which suggests that it could be hemizygous (5AS present in single copy).

To identify additional structural variants, we then computed non-overlapping 100 kb bins with at least two-fold differences in the counts of mapped reads between the parents (Supplementary Data S10). After removing the bins corresponding to chromosome 5AS, a total of 9,259 bins 100 kb long were identified. Adjacent bins were merged into a single region if they were within 200 kb apart. These structural variations are not simple presence/absence variation because in some cases the parent with the lower read count does have mapped reads. We consider that a better term would be read depth variation (RDV). After merging contiguous bins, 2,332 RDVs equal or larger than 100 kb were identified (Supplementary Data S11). Nearly double the number of RDVs showed a bias towards Opata counts (1,534 vs 798), which suggests a higher similarity between Opata and CS than between W7984 and CS.

**Figure 7:**
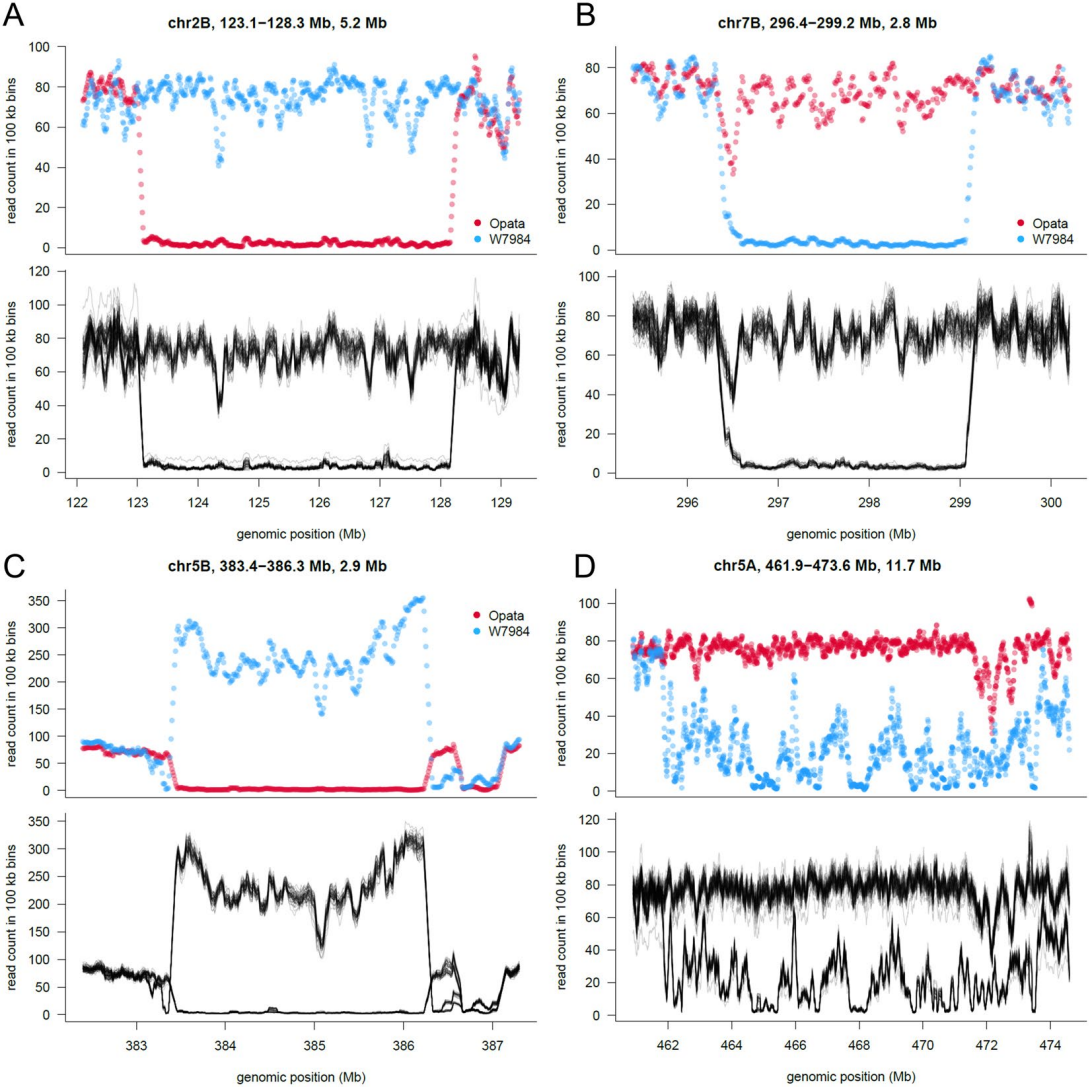
(**A**–**D**) Examples of RDVs. The upper plot of each panel shows the normalized read counts in 100 kb bins for the parental lines W7984 (blue) and Opata (red). Bottom panels show the normalized read counts for the 90 segregating DH lines in the same genomic window. (**A**–**C**) highlights putative PAVs, (**D**) likely underlies a region of sequence divergence between W7984 and CS/Opata. The x-axis shows genomic position according to RefSeq v1.0 in Mb. Titles on each plot indicate the chromosome name, exact genomic positions and size of the particular RDV.

There were nearly double the presence of RDVs in the B genome (1,103) than in the A (633) or D (596) genomes. Chromosome 3B had the largest number of RDVs (185), and chromosome 4D the smallest (33). Some examples of RDVs are shown in Fig. 7. Additionally, Supplementary Data S12 plots show read counts in the parents and the DH segregating lines for the 50 largest RDVs. The largest RDV, after disregarding the missing chromosome 5AS arm, was another region in chromosome 5A between 461.9–473.6 Mb of 11.7 Mb long (Fig. 7D). The most frequent RDV size was 100 kb, with 1,055 occurrences across the genome, followed by 200 kb (355) and 300 kb (211). Supplementary Fig. S7 shows the RDV frequency distribution across normalized chromosome lengths. Although RDVs are widespread, they appear to be more frequent in the distal regions of chromosomes. In fact, the number of 100-kb long RDVs was moderately correlated (ρ_s_ = 0.29 p = 0.036) with the distance to the centromere. To assess the extent of genes that are present in the regions delimited by RDVs, genes that have at least 85% of their length within a RDV were counted. Without considering the chromosome 5AS arm, the 2,332 RDV regions contained a total of 9,196 genes.

To validate the RDVs found, reads were also counted in the same non-overlapping 100 kb genomic windows for the 90 segregating DH lines (Fig. 7, bottom panels). A true RDV must, in addition to having two-fold differences in the counts between the parents, show segregation among the DH lines for the read counts in the approximate expected ratio of 1:1 for high and low counts. We then genetically mapped 100 kb bins of RDV onto the POPSEQ map constructed be Chapman *et al*.^18^. The segregating DHs were assigned to parental haplotypes based on similarity of their normalized read counts to the respective parent. The haplotype vectors of the bins here placed into the POPSEQ map^18^. Bins with a Hamming distance larger than 3 to the nearest POPSEQ marker were considered unmapped. A total of 96.7% of RDV bins could be mapped genetically (Supplementary Data S13). For those that are mapped, the concordance between physical and genetic chromosome assignment is 99.2% with very high colinearity (Supplementary Fig. S8).

## Discussion

The reduced diversity in the elite wheat breeding material, and particularly in the D genome, has hampered marker discovery, linkage map construction, QTL detection, and marker-assisted breeding. Although available since the 1940s, synthetic wheats were not incorporated into breeding programs until the 1980s^19^. Since then, synthetic wheats have introduced novel genetic diversity for important traits. In this study, we mapped two reference populations created by crossing the synthetic wheat W7984 and the variety Opata, and used the maps to study segregation distortion, recombination rates, recombination QTLs, and large structural variations (RDVs). In addition, these high-density maps will be a rich resource for future genetics studies.

### *SynOpRIL* and *SynOpDH* high quality genetic maps

The *SynOpRIL906* and *SynOpDH92* maps have 21 linkage groups, corresponding to the bread wheat haploid set chromosome number, with the B genome having the largest number of markers, followed by the A and D genomes. Strict filtering criteria during marker selection, grouping and ordering, resulted in generating two reliable maps that were validated in the comparisons with the reference genome. The two maps were similar in genetic length, but differed in other features. The *SynOpDH92* map had more framework and imputed markers than *SynOpRIL906*, likely reflecting an increased sequencing depth (three times higher for the SynOpDH population). However, the RIL population design and larger number of individuals in the RIL population increased the overall number of recombination events. In both *SynOp* maps, chromosome 7B had the highest number of markers and chromosome 4D the least. In other published genetic maps, the B genome has been consistently found to be the most polymorphic^20–25^. Other studies have also found the lowest number of markers in the D-genome, compared to the A and B genomes^20–23^. Although the B genome is physically larger than the A and D genomes (518.0 vs 493.4 and 395.1 Mb)^10^, these differences alone cannot account for the differences in polymorphism levels, and suggest that there was a more extensive gene flow from tetraploid (AB) than diploid (D) ancestors to hexaploid wheat, as previously proposed^24,26^, most likely as a result of the more ancient formation of tetraploid wheat. Because of its recent formation and little time for diploidization, a majority of polymorphisms in hexaploid wheat are expected to have arisen from the more ancient AB hybridization event. Nevertheless, the percentage of total markers in the D-genome in our study, ranging from 25.8 to 26.6%, is considerably higher than the 10.8–18% previously found in non-synthetic wheat crosses^10,21,23–25,27^ due to the wild D genome introduced through W7984. In fact, the percentage of markers in the D-genome in other studies that also used W7984 × Opata crosses^8,18,22,25,28,29^ were consistent with the values we obtained (17.8–28.4%).

The density of GBS-SNPs was found to vary greatly among homoelogous groups. Whereas for most groups, the number of SNPs represented about 15% of the total number, the homoelogous group 4 (4A, 4B and 4D) had only 9.3% and 10.5% for SynOpRIL and SynOpDH, respectively. This trend is maintained (4–13.6%) in other hexaploid wheat maps^8,18,20–23,25,29^. Again, these differences cannot be accounted for by the differences in genome size alone since, for instance, the accumulated size of homeologous groups 1 and 6 is smaller than group 4, demonstrating that homeologous group 4 is recombination-poor.

### Recombination rates vary between and within chromosomes

Recombination is necessary to introduce new combinations of genetic variants. We found recombination rates vary within and between wheat chromosomes, and to be negatively correlated with chromosome size. A negative correlation between genome size and recombination rate is common in plants and is likely to be related to the presence of LTR retrotransposons^30^. The negative correlation between recombination rate and LTR retrotransposon content, together with the positive correlation between recombination rate and gene density^10^ supports this hypothesis. The D genome exhibited the largest values (0.26–0.27 cM/Mb), and B the lowest (0.17–0.18 cM/Mb), and confirms previous observations that the D genome recombines more than the other two^10^. Using a cross between CS and Renan, higher recombination rates were found in the A, B and D genomes (0.23, 0.20 and 0.36 cM/Mb, respectively)^10^. Large variation in recombination rates have been observed across populations and individuals^16,31^. The level of polymorphisms is higher in the B genome, followed by the A and D genomes, suggesting that in wheat the recombination rate is negatively correlated with polymorphism level.

Recombination rates also fluctuate considerably among regions within chromosomes, with the lowest values around the centromere region, and the largest towards the end of chromosomes. Correlations between chromosome structure and CO events have been extensively described^30,31^. Although their frequency and magnitude vary greatly from one species to another, there is a common trend for reduced recombination in peri-centric regions. We found that in wheat, COs are mostly limited to subtelomeric regions of chromosomes, thus COs are positively correlated with gene density (ρ_s_ = 0.723 and ρ_s_ = 0.698, p < 2.2E-16, for SynOpRIL and SynOpDH, respectively), in agreement with other whole genome surveys^10,32,33^. Other correlations have been found between recombination rates and physical structure and genome features of chromosomes. Variables related to physical structure are chromosome size, arm size, and distance from the centromere or telomere^10,34^. Genomic features that correlate with recombination events include GC content, CpG, simple repeats and transposable elements^10^. The lack of recombination in the centromeric region results in a significant loss of breeding efficacy for genes located in these regions. Yet, centromeric regions contain 7% of the predicted genes in wheat^10^. Genes within the centromeric and pericentromeric regions will thus reduce the long-term prospects for effective molecular assisted breeding.

The *SynOpRIL906* and *SynOpDH92* maps exhibited a relatively low number of unique recombination breakpoints (bins) (Table 1). Previous studies on single biparental wheat crosses have shown similar results, even when large populations were used^18,21,27,28,35,36^. Two possible factors can contribute to this. First, only high-quality markers with low-missing data were used to construct the framework maps. This reduced the likelihood of tagging all CO events in a large genome. In fact, a positive correlation between the number of markers and bins, which only becomes saturated at numbers in the order of tens of thousands, has been observed in wheat^25^. Wingen *et al*.^16^ found a significant effect of marker number, map size and population size on the detected COs and were able to explain 98% of the variance in the number of COs using a linear model that included those three covariates. Second, we used the RefSeq v1.0 as the anchor genome to identify SNPs. It is estimated that a total of 1.3–1.7 Gb is either missing or assembled in unanchored scaffolds. Most of this non-assembled sequence is expected to be located in the centromeric regions of chromosomes^10^, which avoids detection of GBS-SNPs in these regions when the RefSeq v1.0 is used as the reference genome. This is consistent with the observed bias of GBS-SNP markers towards the distal parts of chromosomes (Fig. 5A and Supplemental Fig. S5A).

### Additive QTLs and epistatic interactions control the number of crossovers

The number of COs accumulated in a single line followed the typical distribution of a quantitative trait. However, despite the large population size, only 2 QTLs passed the test for significance, suggesting that COs are largely influenced by numerous loci with a small individual effect and reduced power from mapping in RILs rather than F_2_ lines. Nonetheless, two QTLs were detected, which accounted for 6.4% (6A) and 3.0% (6D) of the observed variance. The QTL in chromosome 6A was previously reported^16^, while the locus in 6D is a novel QTL. In a given cross, to increase statistical power to detect minor-effect QTLs requires including as many sources of variance as possible^37^. Because effective epistasis detection requires a large sample size, it is not commonly addressed in QTL studies. Our multiple interval mapping models incorporated two-locus interactions, and successfully detected significant epistatic loci in the large SynOpRIL population. Altogether, our results suggest that CO events are largely influenced by minor effect QTLs and epistatic interactions, in agreement with other previous findings^16,17^. Mapping QTL for recombination is notoriously difficult due to the majority of crossovers occurring in heterozygous backgrounds in the early generations of population development, resulting in alleles that restrict crossovers becoming fixed in high-crossover lines or, alternatively, alleles that promote crossing over becoming fixed in low-crossover lines.

### Segregation distortion is a widely distributed phenomenon

Deviation of locus segregation from the expected 1:1 ratio was observed in both SynOpDH and SynOpRIL populations. However, while in the *SynOpDH92* map only 0.44% of markers exhibited significant (p < 0.01) segregation distortion, in the *SynOpRIL906* map the number increased to 15.7%. There were 5 long (>20 cM) SDR blocks with more than 40 markers, all in the SynOpRIL. The fewer significant distorted markers and SDRs in SynOpDH compared to SynOpRIL is likely due to the increased generations of inbreeding and opportunity for inadvertent selection of individuals with higher fitness. The only segregating distorted region found in SynOpDH, in chromosome 4D, was also present in SynOpRIL, both showing deviation towards W7984 alleles. This region was previously shown to have a bias towards W7984 alleles^18^, and suggests that a general mechanism, not linked to the development of DH or RIL populations, is affecting segregation patterns.

A larger number of SDRs were found in SynOpRIL. Most of the SDRs and markers that exhibited segregation distortion in *SynOpRIL906* were located in the B genome. The distorted regions on the short arm of 2B and long arm of 5B are collocated with the hybrid necrosis genes *Ne2* and *Ne1*, respectively. Certain epistatic combinations of *Ne2* and *Ne1* alleles are known to result in segregation distortion on those two regions, and to cause necrotic cell death at the seedling stage in intraspecific crosses in wheat^38^. In fact, markers near to the *Ne2* and *Ne1* loci are in linkage disequilibrium in the SynOpRIL population (χ^2^, p = 5.32E-12). Hybrid progenies from synthetic wheat and common wheat crosses carrying deleterious allele combinations of these two loci have been shown often to be affected and die at the early seedling stage^19^.

Cytoplasmic male sterility is characterized by the inability to produce viable pollen and results from incompatibility between nuclear and cytoplasmic genomes. The restoration of pollen fertility is under the control of restorers of fertility (*Rf*) genes. Restorer of fertility-like genes (*RfL*) tend to cluster in regions in the genome. *RfL* clusters have been found on the short arm of chromosomes 1A, 2D, 6A and 6B^10,39^, which co-localize with segregating distorted areas in the *SynOpRIL906* map. Other mechanisms like preferential pollination, abortion of male/female gametes, variation in seed dormancy, and lethal epistatic interactions are causes of unbalanced allele frequencies^40^, and may be also involved in the segregation distortion of the other SDR.

### Genome-wide RDVs are numerous and correlate with distance to the centromere

The most noticeable finding of the genome-wide RDV analysis is that the whole short arm of chromosome 5A is missing in the W7984 parent. In support of this assertion, first, there is a lack of markers in the initial distal region on both maps. The first marker in the *SynOpRIL906* and *SynOpDH92* maps corresponds to the genomic position 258,789,917 and 316,659,742 bp, respectively. Thus, there is a large gap of at least 258.7 Mb in both maps where no GBS markers were found. Second, a sharp drop of read counts that spans 258.3 Mb was identified when the W7984 sequenced reads were mapped to the RefSeqv1.0. Despite that the W7984 × Opata reference population has been used extensively, to our knowledge this deletion has not been reported before, probably because of the previous lack of an anchoring reference genome. Because of this whole arm deletion, the W7984 × Opata population could be useful to study genes present in the short arm of chromosome 5A.

The fact that a considerably higher number of RDVs showed a bias towards Opata counts implies that Opata is more similar to CS than what W7984 is, as expected by the synthetic origin of W7984. Our method can reveal the following structural variations: (i) deletions present in one of the parents (W7984 or Opata), and not in the other, as long as the same deletion is not present in the CS reference; (ii) insertions in one parent and not in the other, as long as the CS reference shares the same insertion; (iii) copy number variations (CNVs) between W7984 and Opata, regardless of the copy number in the reference; and (iv) lastly, sequence divergence between the parents and CS. For some of the putative large CNV/PAV, there are a considerable number of reads mapped per bin in both parents, but still a large log2 ratio, suggesting sequence differences from CS so that reads cannot be mapped. Our 2-way validation suggests that most RDVs reflect true genomic structural differences. However, in some instances, the underlying particular structural element might be difficult to assess (ex. Figure 7D). In general, insertions/deletions will be less problematic to identify than CNV or sequence divergence.

Despite that our approach detected a limited number of structural variants, the elevated number of RDVs found suggests that underlying structural variation is very common in wheat. Structural genomic variation such as PAVs and CNVs have been recognized to have the potential to generate phenotypic variation in maize^41^, and barley^42^. The presence of 9,196 high confidence genes within RDVs suggests that they can also be a significant source of diversity between wheat accessions, consistent with other studies^43^. There were nearly twice as many RDVs of at least 100 kb long in the B genome than in the A and D genomes, which confirms the findings on GBS-SNP marker distribution pointing at the B genome as the most polymorphic. This is also in agreement with the lower synteny observed between the B genome chromosomes and the A and D homeologues as compared to the synteny between the A and D genome homeologues^44^.

We found RDVs to be more frequent in the distal parts of chromosomes. This is in agreement with a higher occurrence of SNPs and other polymorphisms in these areas, and with the decreased level of synteny observed between homeologous chromosomes as the distance of a chromosome region to the centromere increases^43,44^. In fact, the higher recombination rates in subtelomeric regions indicates that they are fast-evolving and thus more prone to changes. Rimbert *et al*.^23^ also found high presence of PAVs in the distal regions of wheat chromosomes. Akhunov *et al*.^34^ found that duplicated loci were most often located in these distal regions, and their distribution was positively correlated with the recombination rate. Alternatively, as mentioned earlier, the part of the reference sequence that is still not assembled is likely located in the centromeric regions, and could also be a contributing factor explaining the less abundant PAVs in these regions. Nevertheless, there is also an important number of RDVs in the proximal regions. Retrotransposons constitute a substantial part of the wheat genome and contributes to structural variation^10^. It has been suggested that the reduction in recombination in regions with a high density of retroelements can be due to structural variations created by retrotransposon insertions or deletions^33^.

In summary, because of its recent formation, hexaploid wheat suffers from a narrow genetic diversity, further aggravated by the process of domestication. We have constructed two reliable linkage maps using the synthetic W7984 × Opata RIL and DH populations. Synthetic crosses have the potential to increase wheat genetic diversity, thus the maps will have major value not only in breeding programs but also in genetic and genomic studies. The maps, together with the previously reported low-depth sequencing coverage of the DH population^18^, allowed us to examine recombination rates and segregation distorted regions and QTLs associated with genetic control of recombination, as well as investigate the genomes of W7984 and Opata for the presence large genomic variants.

## Materials and Methods

### Plant material and DNA extraction

Two bi-parental mapping populations from crosses between the parental lines Synthetic W7984 (also known as M6) and Opata M85 (abbreviated W7984 × Opata) are described in detail in Sorrells *et al*.^8^. W7984 is a synthetic line derived from a cross between the durum (*T*. *turgidum* L.) genotype Altar 84 and *Ae*. *tauschii* Coss., the progenitor of the bread wheat D genome. We used a random subset of the larger populations initially created. These populations are: 1) an F_1_-derived population of double haploids (DH) of 92 individuals, a subset of the original SynOpDH population of 215 lines, and 2) a recombinant inbred population (RIL) of 1,100 F_6_ lines derived by single seed descent, a subset of the original SynOpRIL population of 2,039 lines^8^. Plants were grown in a greenhouse and genomic DNA was extracted from seedlings of each line.

### Genotype-by-sequencing and SNP calling

For the SynOpDH population, we followed a two-enzyme restriction digestion GBS protocol first described in Poland *et al*.^22^. It uses one rare-cutter and one common-cutter to generate a uniform complexity reduction of the genome. Briefly, the GBS libraries were constructed in 48-plex and genomic DNA was digested with the restriction enzymes *PstI* (CTGCAG) and *MspI* (CCGG). Barcoded adapters were ligated to individual samples and pooled. For the SynOpRIL population, the DArTseq library construction was used^22^, which involves only *PstI* fragments (e.g. *PstI* on both ends) followed by further complexity reduction with *HpaI* (GTTAAC). Libraries were constructed in 96-plex. This DArT approach produces much more complexity reduction than the two enzyme GBS and is also expected to produce fewer markers, though with better coverage. Because *PstI* was used as the primary restriction site for libraries in both populations, the sequencing sites have significant overlap.

Libraries were single-read ultra-light sequenced on the Illumina GAII or HiSeq2000 platforms, at a sequence depth of 0.03× and 0.01× , respectively. From the raw sequence data, reads were demultiplexed according to their barcodes using a script for demultiplexing^45^. Reads were assigned to samples based on their initial barcode followed by the TGCA overhang sequence. Only exact matches were considered. The barcode + TGCA were trimmed off the reads. Reads from different libraries that belonged to the same individual were combined in the same file. Only reads above 30 bp long were kept (tags).

Sequence tags were aligned to the RefSeq v1.0 CS reference genome using the Burrows-Wheeler Aligner (BWA)^46^ with the *bwa-mem* algorithm v0.7.15, the –T parameter set to 40 to output alignments with a quality score equal or above 40, and otherwise default parameters. The alignment output files in ‘*sam’* format were converted to binary versions ‘*bam’* with the *view* algorithm and sorted with *sort* algorithm within samtools v1.6^47^. A *bcf* file was generated with samtools *mpileup* with –uv –t INFO/AD,DP,AD parameters to output number of high-quality bases and allelic depth. SNP calling was done using bcftools v1.6 *call*^48^ with the parameters –c –v -f GQ. The output *vcf* file was filtered with the following constraints: a homozygous genotype call was retained if at least one read supported it; two reads were needed to support a heterozygous call. The SNP calls with quality scores below 40, more than 25% missing data, more that 10% heterozygous calls, or a minor allele frequency below 20% were discarded. Only high quality biallelic SNP positions with successful genotype calls in both parents and homozygous for opposite alleles were retained for the SynOpDH population. Heterozygote calls were allowed in the SynOpRIL population, other than in the parents, and used to compute statistics. Individual’s heterozygous calls were set to missing previous to linkage grouping. GBS-SNP markers were given the following format: “chr1A_13829065”. The first part corresponded to the CS chromosome, and the second, after “_”, corresponded to the physical position on the RefSeq v1.0 assembly. In house *bash* and *perl* scripts were developed to perform the preceding tasks.

### Linkage map construction and genetic analyses

MSTMap^49^ on the R/ASMap^50^, and the R/qtl^51^ packages for R were used for linkage group construction, genetic mapping and computing of linkage statistics. During map construction, lines that were sequenced at insufficient depth, had a high rate of missing or heterozygous observations, a high number of double crossovers, or shared a proportion of their alleles greater than 0.95 were removed. GBS-SNP markers that aligned to the RefSeq v1.0 “Unanchored” scaffolds pseudochromosome (ChrUn) were removed for subsequent analysis. Initial group assignment was established using a p-value of 1E-30 and 1E-6 for the SynOpRIL and SynOpDH population, respectively, and the maximum likelihood (ML) objective function. Population types were set to RIL6 and DH, respectively. Recombination frequencies were converted to centiMorgans (cM) using the Kosambi function. Markers with minor allele frequencies of 0.20 or less for either parental allele were removed prior to map construction. For the framework map construction, the maximum rate of missing observations was set to 0.08. Default settings were allowed for other parameters. Stringent filtering removed low-quality markers, with excess of missing or heterozygous sites, prior to map construction (see previous section). Markers with excess of double recombination or suspected to be wrongly positioned were also removed during each iteration of linkage map construction. Recombination rates (cM/Mb) were computed by dividing genetic into physical distances in 25 Mb windows using a sliding window of 2.5 Mb. Recombination plots were drawn using R and standard functions. Tests for segregation distortion were performed by checking departure of allele proportions from the expected 1:1 ratio, after Bonferroni correction for multiple testing.

QTL mapping was achieved with the R/qtl package. The functions *cim* and *scantwo* were first used to highlight candidate loci (LOD > 2.0) and interactions genome-wide. The *cim* function performs a composite interval mapping with a forward selection at the markers to a fixed number, followed by interval mapping with the selected markers as covariates, dropping marker covariates if they are within a fixed window size of the location under test. Tests for significant locus-trait associations were performed by means of a Haley-Knott regression with a window of 20 cM, 1 cM steps, and 7 marker covariates to account for variance genome-wide. The function *scantwo* tests for epistatic interactions, also using a Haley-Knott regression. Highlighted candidate loci and interactions were fitted in a multiple interval mapping (MIM) model and tested iteratively using the *fitqtl* function. A stable model was reached with 10 loci and 3 interactions: y ~ Q1 + Q2 + Q3 + Q4 + Q5 + Q6 + Q7 + Q8 + Q9 + Q10 + Q3:Q11 + Q3:Q9 + Q5:Q6 (Tables S2 and S3). Permutation tests with 1,000 replicates were used to test for significance (p = 0.05), and established thresholds of LOD 5.5 and 12.8 for additive and epistatic QTLs, respectively.

### Marker imputation

Error correction and imputation of missing genotypes were completed with the R package LaByRInth (https://github.com/Dordt-Statistics-Research/LaByRInth), a version for R of the original LB-Impute algorithm^15^ using default settings. LB-Impute uses a hidden Markov model to impute the missing sites in a low-coverage biallelic population where the parental genotypes are known, even when per-marker coverage is extremely low, as for GBS datasets. LB-Impute also minimizes false homozygous calls at heterozygous sites by incorporating depth-of-sequencing read coverage into the imputation algorithm. The GBS-SNP markers were considered candidates for imputation if they had no more than 25% missing rate, no more than 10% heterozygous calls and a minimum of 20% minor allele frequency. Imputed markers were subsequently filtered to eliminate markers whose maximum fraction of heterozygous calls was higher than 0.05, minimum allele frequency lower than 0.20 and maximum missing rate of 0.10, using in house scripts. Heterozygous calls were set to missing before incorporation to the framework map. Imputed markers were assigned to bins based on their Hamming distances, the number of different, non-missing, genotypes. Hamming distances were computed as in Mascher *et al*.^52^ to find the imputed markers with the least distance to any framework marker. An imputed marker was incorporated to its most likely position in the map if all the markers in the framework map with the least Hamming distance to the imputed marker were located in the same bin. A *perl* script was developed for this step (Supplementary Data S14).

### Read depth variation analysis

For the RDV analysis, we downloaded the low-depth (1.37 ± 0.20×, mean ± SD) sequencing data for the 90 individuals of the SynOpDH population^18^ sequenced with 2 × 150 paired end Illumina reads on the HiSeq2000. Parental lines W7984 and Opata were sequenced at higher depth, approx. 34×and 19× coverage, respectively (see Chapman *et al*.^18^ for more details). Here, downloaded sequenced reads were mapped against the RefSeqv1.0 using *bwa-mem* v0.7.15 –T parameter set to 20. The ‘*sam’* files were converted to ‘*bam’* with the *view* algorithm, and sorted with the *sort* algorithm within samtools v1.6^47^. Duplicates were removed using Picard tool’s Markduplicates (http://broadinstitute.github.io/picard). Mapped reads were counted with samtools *view* in 100 kb windows with the parameters -c -F260 -q 10 to exclude non-uniquely mapped reads. Read counts were imported to R for further processing. Regression curves were drawn with the Lowess function in R, a non-parametric regression method that combines multiple regression models in a k-nearest-neighbor-based meta-model^53^.

## Supplementary information

Supplementary Information

Dataset S1

Dataset S2

Dataset S3

Dataset S4

Dataset S5

Dataset S6

Dataset S7

Dataset S8

Dataset S9

Dataset S10

Dataset S11

Dataset S12

Dataset S13

Dataset S14

## Data Availability

Raw reads have been deposited at SRA (accession number SRP134280).
