# Supplementary material for: Dense genotyping-by-sequencing linkage maps of two Synthetic W7984×Opata reference populations provide insights into wheat structural diversity
Source: Sci Rep. 2019 Feb 11;9:1793. doi: 10.1038/s41598-018-38111-3 (PMC6370774; doi:10.1038/s41598-018-38111-3)
Supplement: Supplementary file 1 — Supplementary Information [file 41598_2018_38111_MOESM1_ESM.pdf]

Dense genotyping-by-sequencing linkage maps of two Synthetic W7984×Opata reference populations provide insights into wheat structural diversity

Juan J. Gutierrez-Gonzalez[1], Martin Mascher[2,3], Jesse Poland[4] and Gary Muehlbauer[1,5]

[1]Department of Agronomy and Plant Genetics, University of Minnesota, St. Paul, MN 55108, USA

[2]Leibniz Institute of Plant Genetics and Crop Plant Research (IPK), D-06466 Seeland OT Gatersleben, Germany

[3]German Centre for Integrative Biodiversity Research (iDiv) Halle-Jena-Leipzig, Deutscher Platz 5e, 04103 Leipzig, Germany

[4]Wheat Genetics Resource Center, Department of Plant Pathology, Kansas State University, 4024 Throckmorton Plant Sciences Center, Manhattan, KS 66506, USA

[5]Department of Plant and Microbial Biology, University of Minnesota, Saint Paul, MN 55108, USA

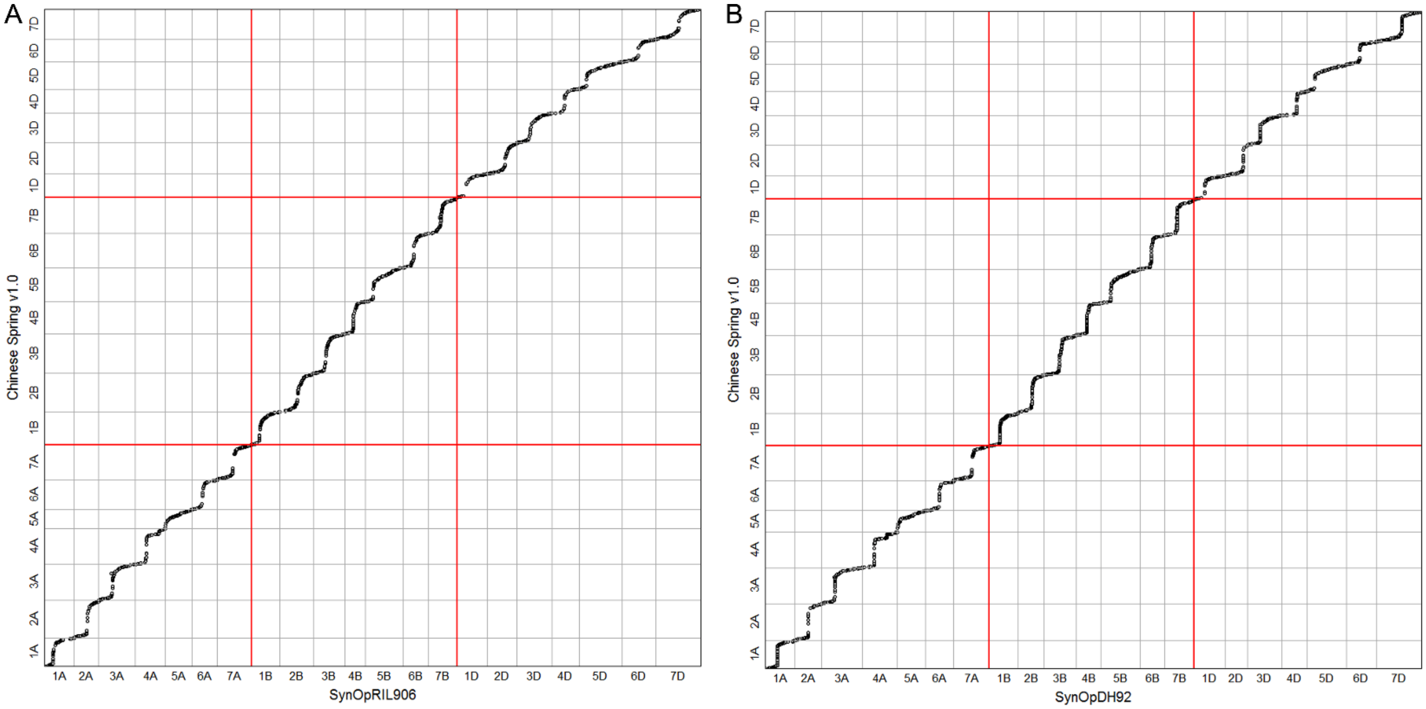

Fig. S1. Dot plot showing the alignments of physical (RefSeq v1.0) vs. genetic position for (A) SynOpRIL906, and (B) SynOpDH92

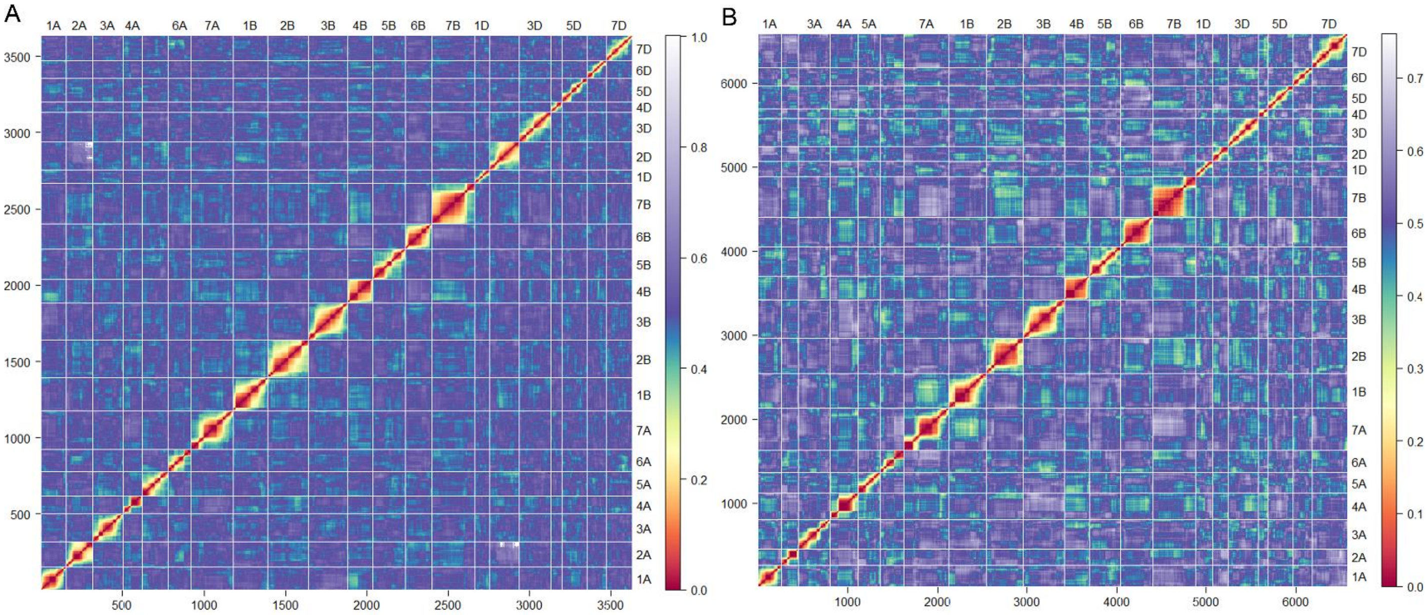

Fig. S2. Recombination fraction heat plots reflecting the strength of linkage between markers for (A) SynOpRIL906, and (B) SynOpDH92 linkage maps. Labels on bottom and left shows the marker number. Labels on top and right display chromosome names. Bars on the right of each plot show the legends for the recombination fractions, from strong linkage (red) to unlinked markers (blue).

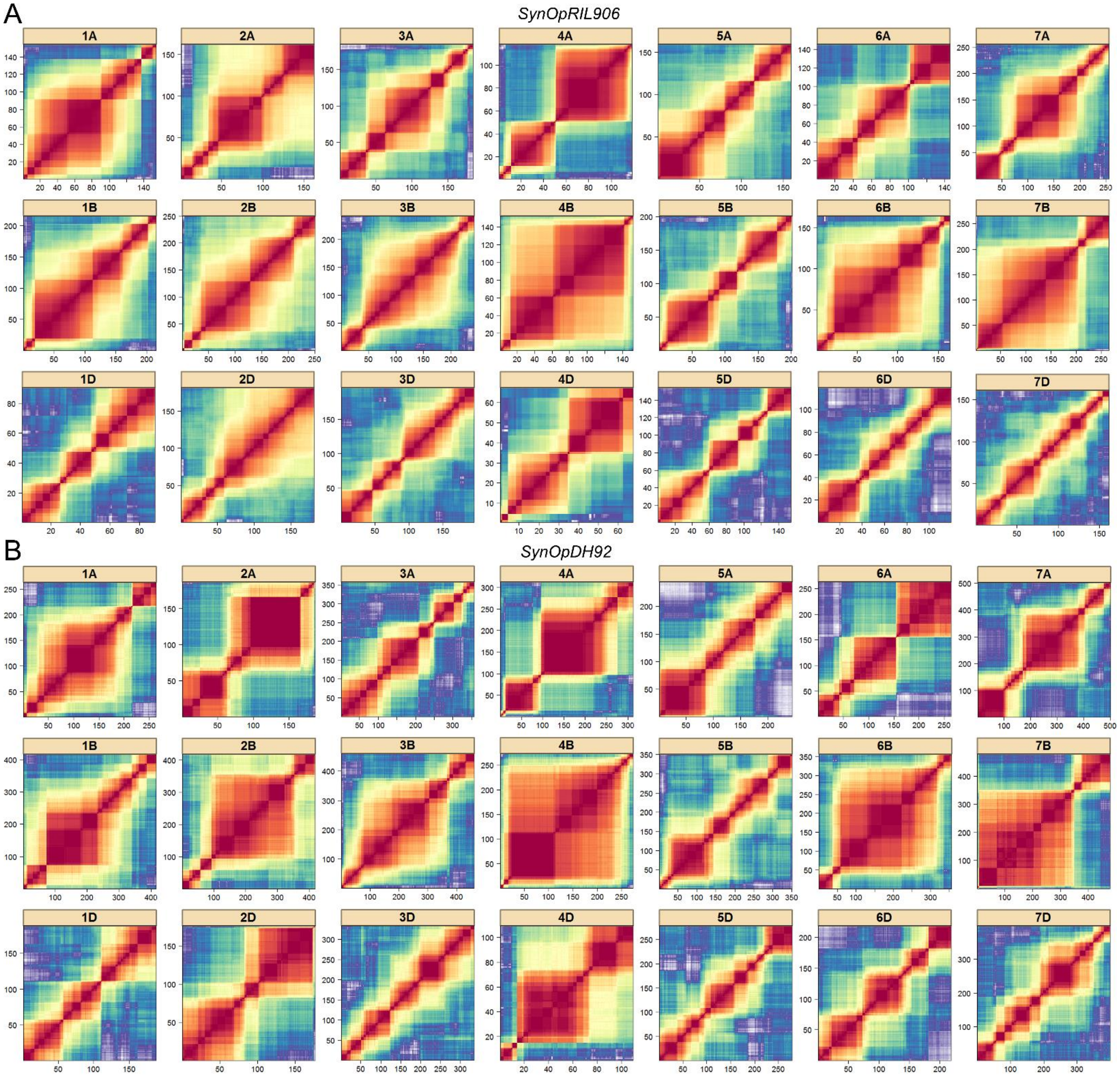

Fig. S3. Recombination fraction heat plots for each individual linkage group for (A) SynOpRIL906, and (B) SynOpDH92 linkage maps. Labels on bottom and left shows the marker number. The color code for recombination fractions is the same than for Fig. S2, and ranges from strong linkage (red) to unlinked markers (blue).

Table S1. Summary statistics including imputed markers

Chr	SynOpRIL906						SynOpDH92					
	n^{o} of imp mkrs	Total n^{o} of mkrs	Markers (%)	Bins	Length (cM)	Marker/cM[a]	n^{o} of imp mkrs	Total n^{o} of mkrs	Markers (%)	Bins	Length (cM)	Marker/cM[a]
1A	2247	2401	5.22	81	126.2	17.81	4990	5253	5.80	41	132.8	37.57
1B	3136	3351	7.29	113	123.6	25.37	5066	5482	5.89	46	128.4	39.46
1D	900	990	2.09	63	131.7	6.83	1778	1966	2.07	52	140.1	12.69
2A	1478	1640	3.44	74	106.2	13.92	2943	3129	3.42	30	119.3	24.67
2B	2868	3118	6.67	134	143.8	19.95	6068	6486	7.05	42	117.6	51.60
2D	1619	1800	3.76	113	127.7	12.68	3317	3493	3.86	36	98.6	33.63
3A	1811	1995	4.21	99	160.1	11.31	3673	4030	4.27	52	183.6	20.00
3B	3184	3426	7.40	125	138.3	23.03	5883	6343	6.84	56	144.4	40.75
3D	2257	2453	5.25	108	152.6	14.79	4696	5030	5.46	58	155.2	30.25
4A	1698	1816	3.95	63	130.9	12.97	3334	3646	3.87	43	158.6	21.02
4B	2868	3020	6.67	67	88.8	32.30	7578	7851	8.81	29	112.7	67.24
4D	711	778	1.65	44	116.8	6.09	1446	1555	1.68	24	114.4	12.64
5A	1426	1585	3.32	87	114.9	12.41	2602	2846	3.02	43	125.8	20.68
5B	2134	2335	4.96	121	162.1	13.17	4493	4844	5.22	63	176.6	25.44
5D	1435	1589	3.34	89	186.2	7.71	2734	3012	3.18	62	177.8	15.38
6A	1534	1679	3.57	79	107.5	14.27	3095	3358	3.60	32	128.8	24.03
6B	2077	2241	4.83	81	109.5	18.97	5003	5359	5.81	46	111.7	44.81
6D	1291	1410	3.00	82	147.4	8.76	2464	2682	2.86	48	135.4	18.20
7A	2740	2995	6.37	115	149.5	18.33	4525	5027	5.26	60	162.0	27.94
7B	3282	3547	7.63	113	124.4	26.39	5571	6049	6.47	38	132.5	42.05
7D	2317	2478	5.39	105	194.0	11.94	4783	5181	5.56	72	204.6	23.38
A genome	12934	14111	30.07	598	895.3	14.45	25162	27289	29.24	301	1011.0	24.89
B genome	19549	21038	45.45	754	890.4	21.96	39662	42414	46.10	320	923.8	42.93
D genome	10530	11498	24.48	604	1056.3	9.97	21218	22919	24.66	352	1026.1	20.68
Total	43013	46647		1956	2842.1	15.13	86042	92622		973	2961.0	29.06

[a]Marker density: number of markers per cM

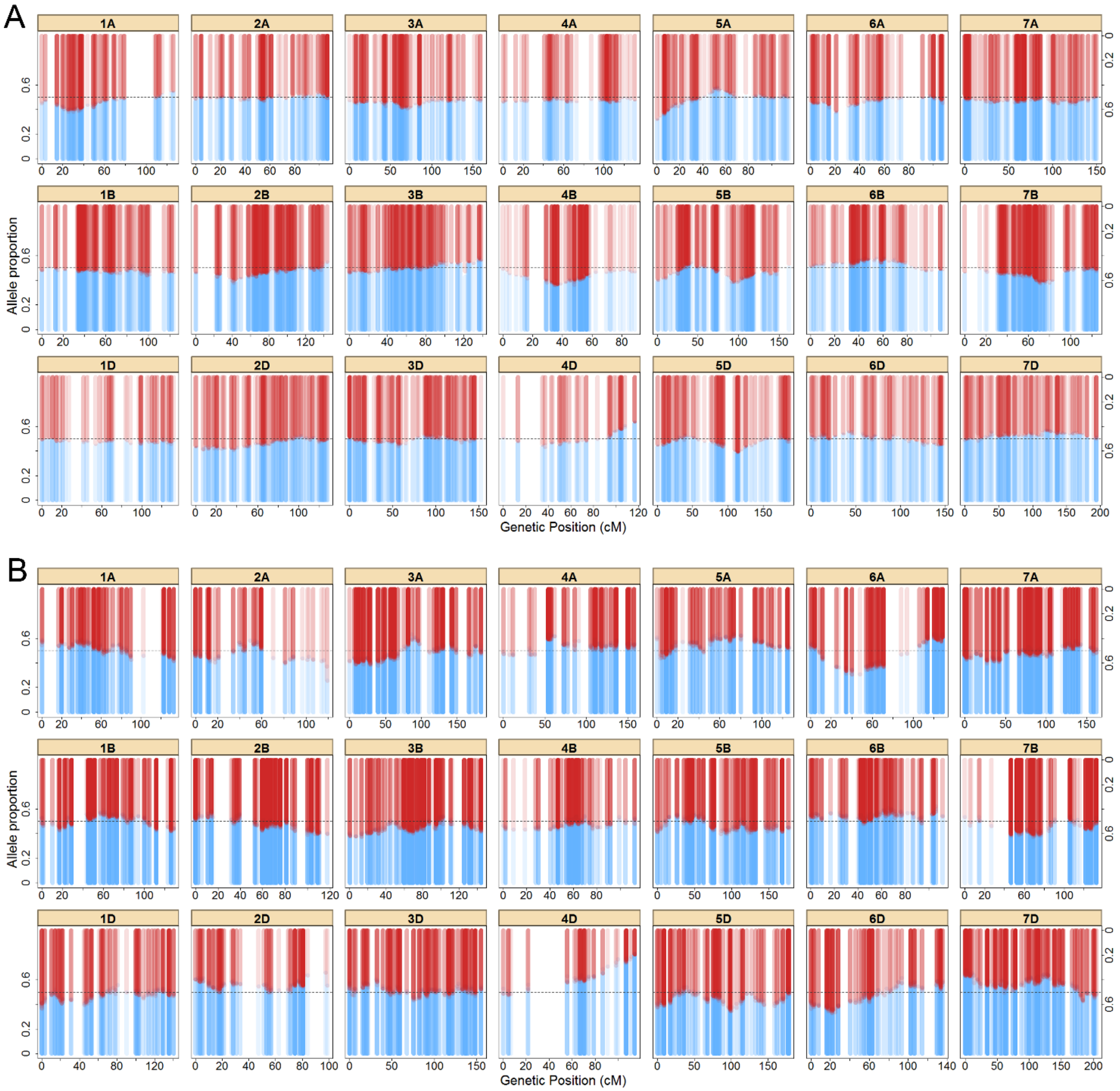

Fig. S4. Parental allele frequencies for the (A) SynOpRIL906, and (B) SynOpDH92 linkage maps. X-axis show the marker number position in the maps for each linkage group. Y-axis shows the allele frequency for the W7984 alleles (blue) on the left, and for the Opata alleles (red) on the right.

Table S2. Recombination frequencies.

Chr	SynOpRIL906		SynOpDH92	
	cM/Mb	1cM = xMb[a]	cM/Mb	1cM = xMb
1A	0.21	4.67	0.23	4.43
1B	0.18	5.53	0.19	5.34
1D	0.27	3.74	0.28	3.51
2A	0.14	7.34	0.16	6.41
2B	0.18	5.52	0.15	6.81
2D	0.20	5.01	0.15	6.50
3A	0.21	4.67	0.24	4.09
3B	0.17	5.99	0.17	5.74
3D	0.25	4.02	0.25	3.96
4A	0.18	5.66	0.21	4.68
4B	0.13	7.44	0.17	5.94
4D	0.23	4.33	0.23	4.39
5A	0.29	3.41	0.28	3.57
5B	0.23	4.34	0.25	4.00
5D	0.33	3.01	0.31	3.18
6A	0.17	5.72	0.21	4.79
6B	0.15	6.49	0.16	6.41
6D	0.31	3.21	0.29	3.49
7A	0.20	4.90	0.22	4.54
7B	0.17	6.02	0.18	5.66
7D	0.31	3.23	0.32	3.11
A genome	0.19	5.26	0.22	4.60
B genome	0.17	5.88	0.18	5.58
D genome	0.27	3.70	0.26	3.82
**Total**	**0.21**	**4.76**	**0.22**	**4.64**

[a]number of Mb to which 1 cM corresponds

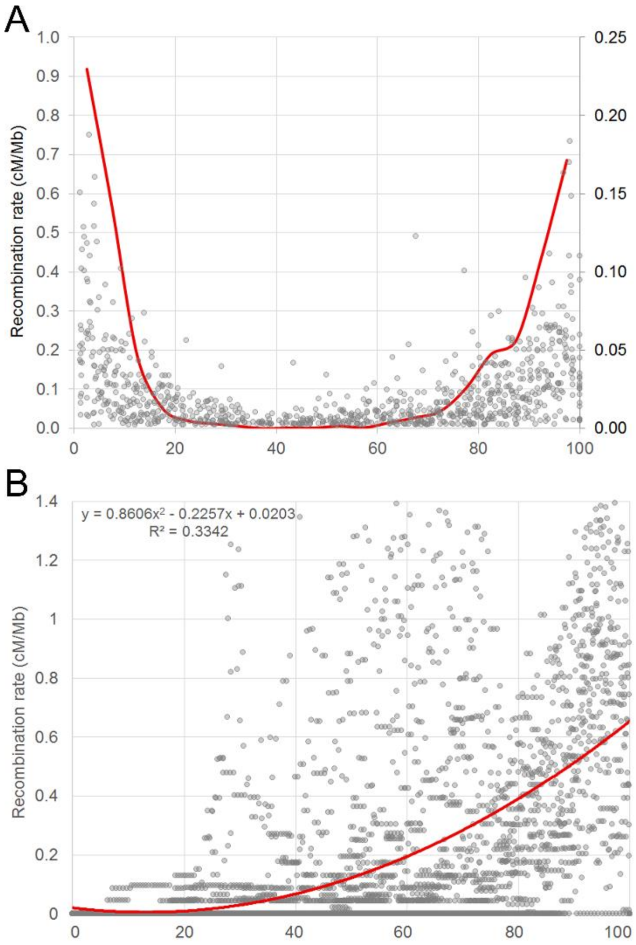

Fig. S5. Recombination trend (cM/Mb) along wheat genome was calculated for the *SynOpDH92* map. A) Physical distances were normalized either by the relative length of each chromosome (A) or by the relative distance from the centromere (B). Dots in (A) represent the relative frequency of GBS-SNP markers across normalized chromosomes; while in (B) represent each recombination rate datapoint, with the curve and the equation that best fits the data. Centromere positions were obtained from IWGSC, 2018.

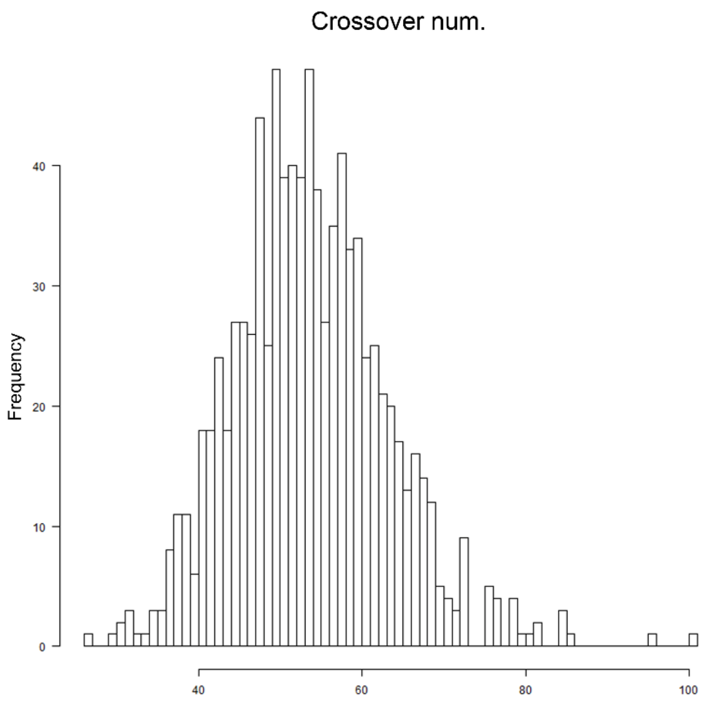

Fig. S6. Histogram phenotypic data (number of crossovers per individual) of the recombinant inbred individuals from the W7984×Opata cross. Numeric phenotypes are displayed as histograms with approximately 2 sqrt(n) bins. X-axis shows the crossover counts.

Table S3. Main additive QTLs for recombination

QTL	closest marker	Chr	Position	LOD	Effect	R2 (%)	pvalue
Q1	chr1A_22047070	1A	21.0	2.5	-0.98	1.0	0.003
Q2	chr5A_545129828	5A	31.1	3.1	0.71	1.3	0.001
Q3	chr6A_23415012	6A	16.0	15.1	0.12	6.4	0.000
Q4	chr1D_406977757	1D	64.4	2.3	0.77	0.9	0.005
Q5	chr2D_33322973	2D	36.0	3.7	0.66	1.5	0.009
Q6	chr2D_473987794	2D	86.0	4.6	0.45	1.9	0.002
Q7	chr4D_336833335	4D	54.1	3.7	0.99	1.5	0.000
Q8	chr6D_102473350	6D	67.1	3.5	1.13	1.5	0.000
Q9	chr6D_445773103	6D	118.0	7.1	-0.39	3.0	0.000
Q10	chr7D_12549862	7D	14.0	2.2	0.00	0.9	0.006

Table S4. Epistatic interactons affecting recombination

QTL	Interacting loci	pos1	pos2	LOD	pval	R2 (%)
Q3-Q11	c6A:c3D	16.0	122.0	14.62571	0.004	2.46
Q3-Q9	c6A:c6D	16.0	118.0	13.89673	0.021	2.31
Q5-Q6	c2D:c2D	36.0	86.0	13.83496	0.021	1.22

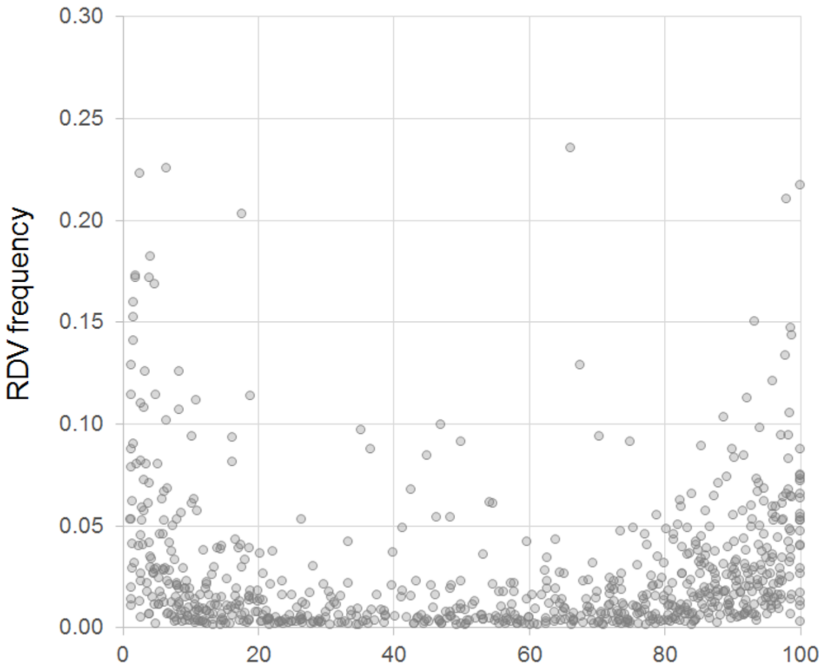

Fig. S7. RDV frequency distribution across normalized chromosome lengths.

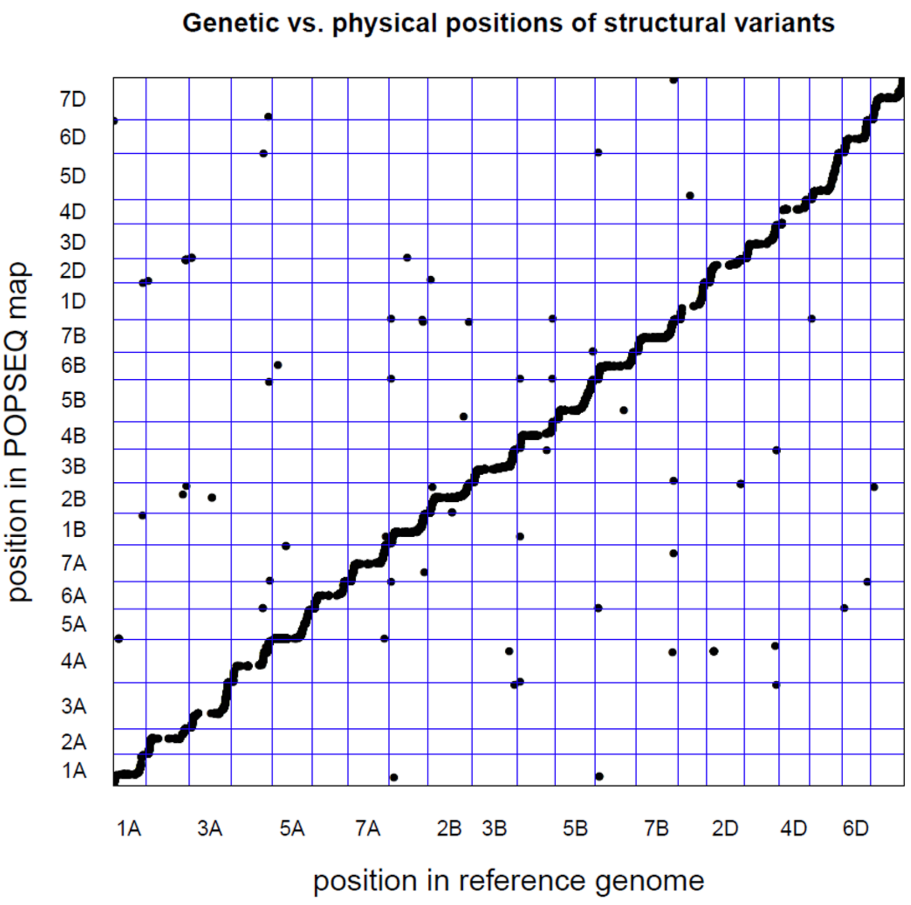

Fig. S8. Collinearity plot showing the comparison of physical RDV positions vs. POPSEQ genetic positions in Chapman et al., 2015.
