# Supplementary figures and images for: Dense genotyping-by-sequencing linkage maps of two Synthetic W7984×Opata reference populations provide insights into wheat structural diversity
Source: Sci Rep. 2019 Feb 11;9:1793. doi: 10.1038/s41598-018-38111-3 (PMC6370774; doi:10.1038/s41598-018-38111-3)

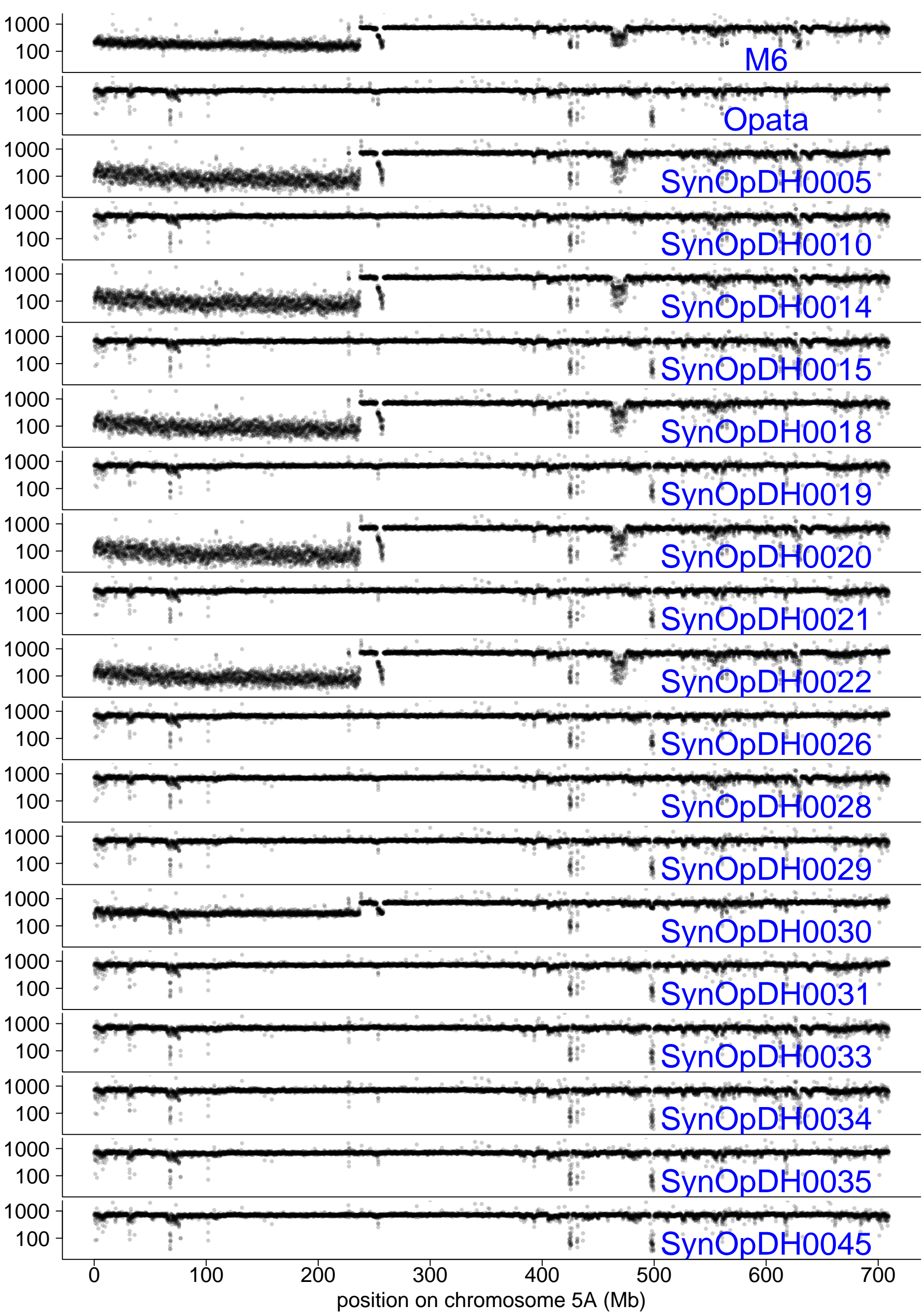

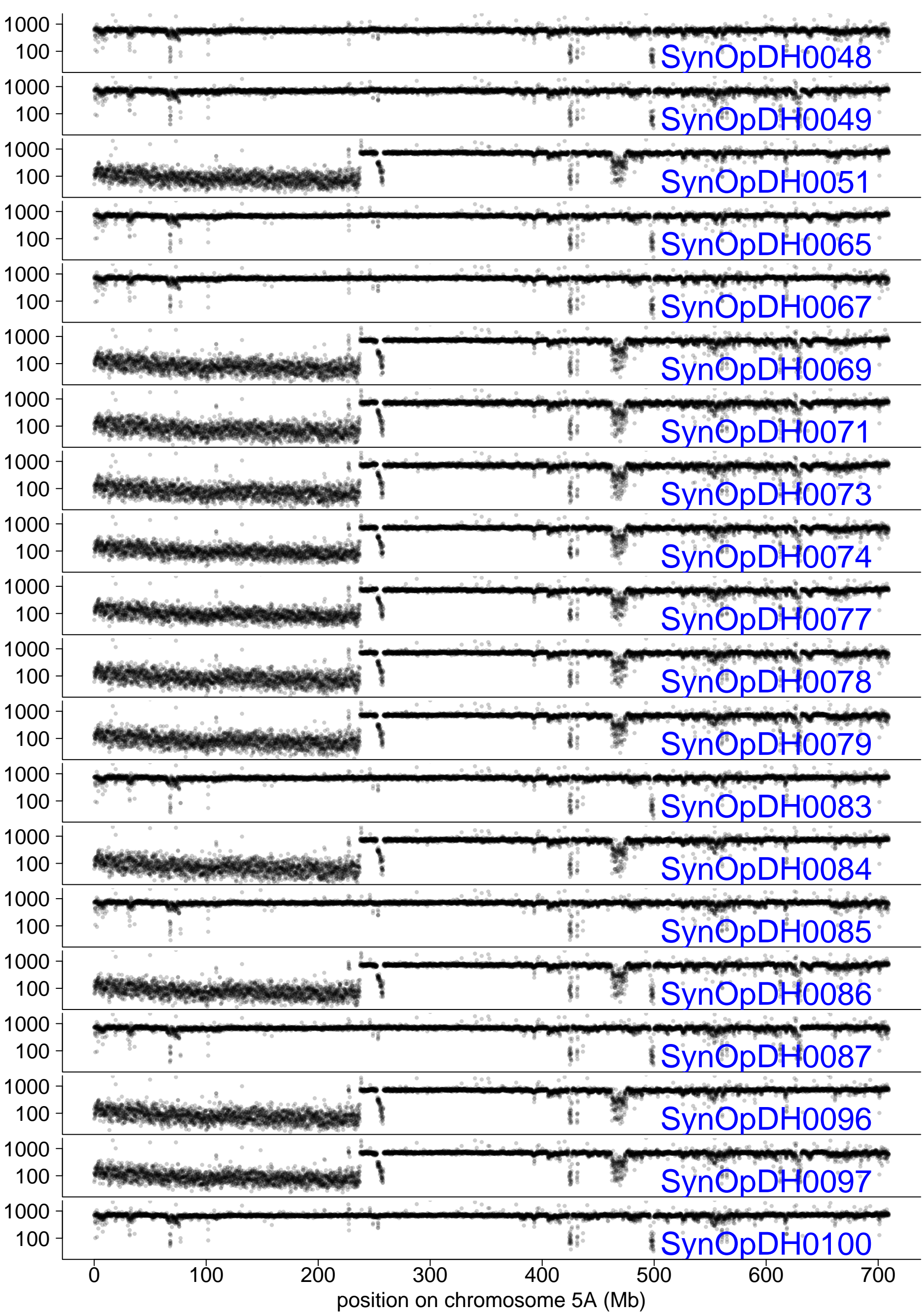

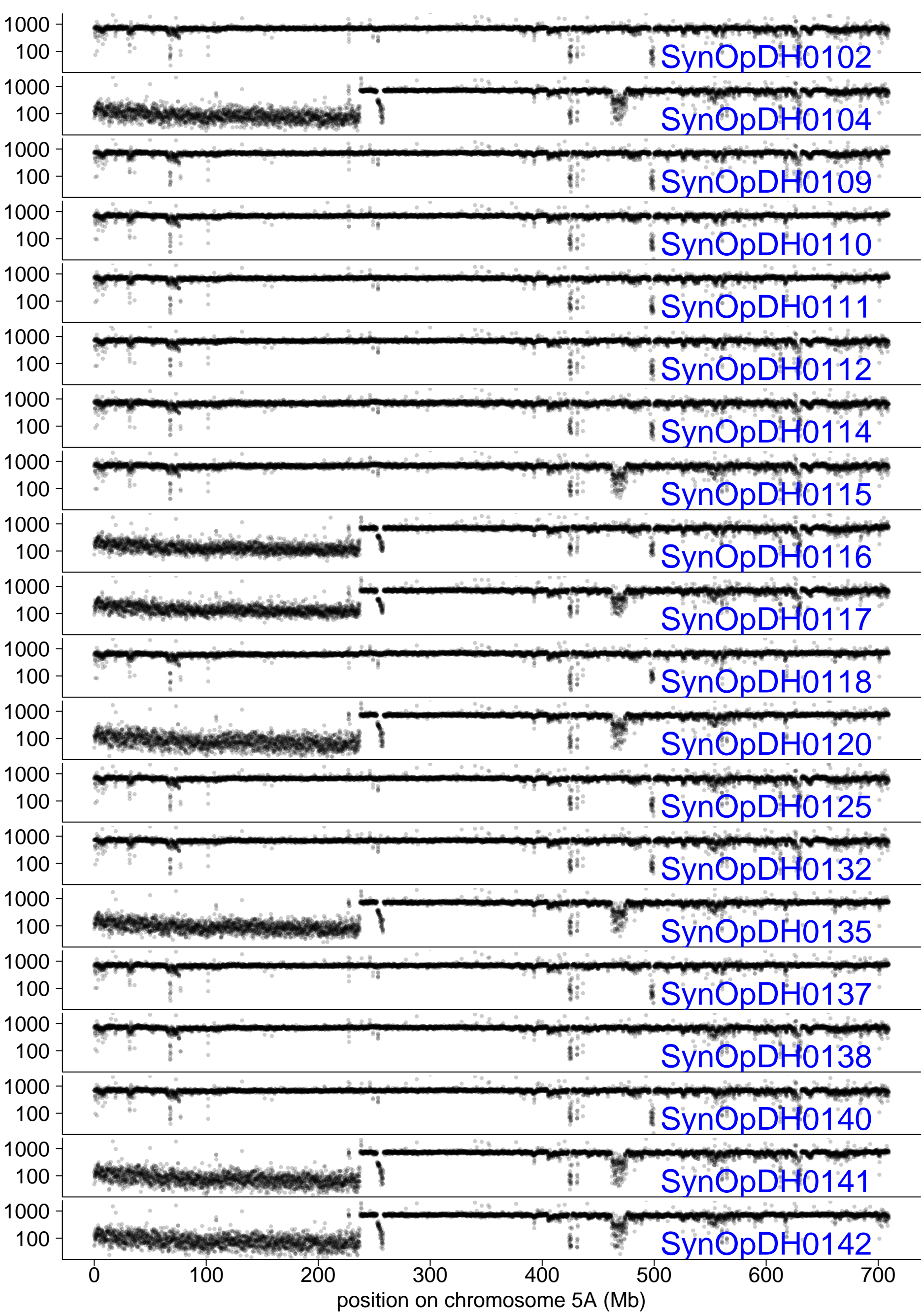

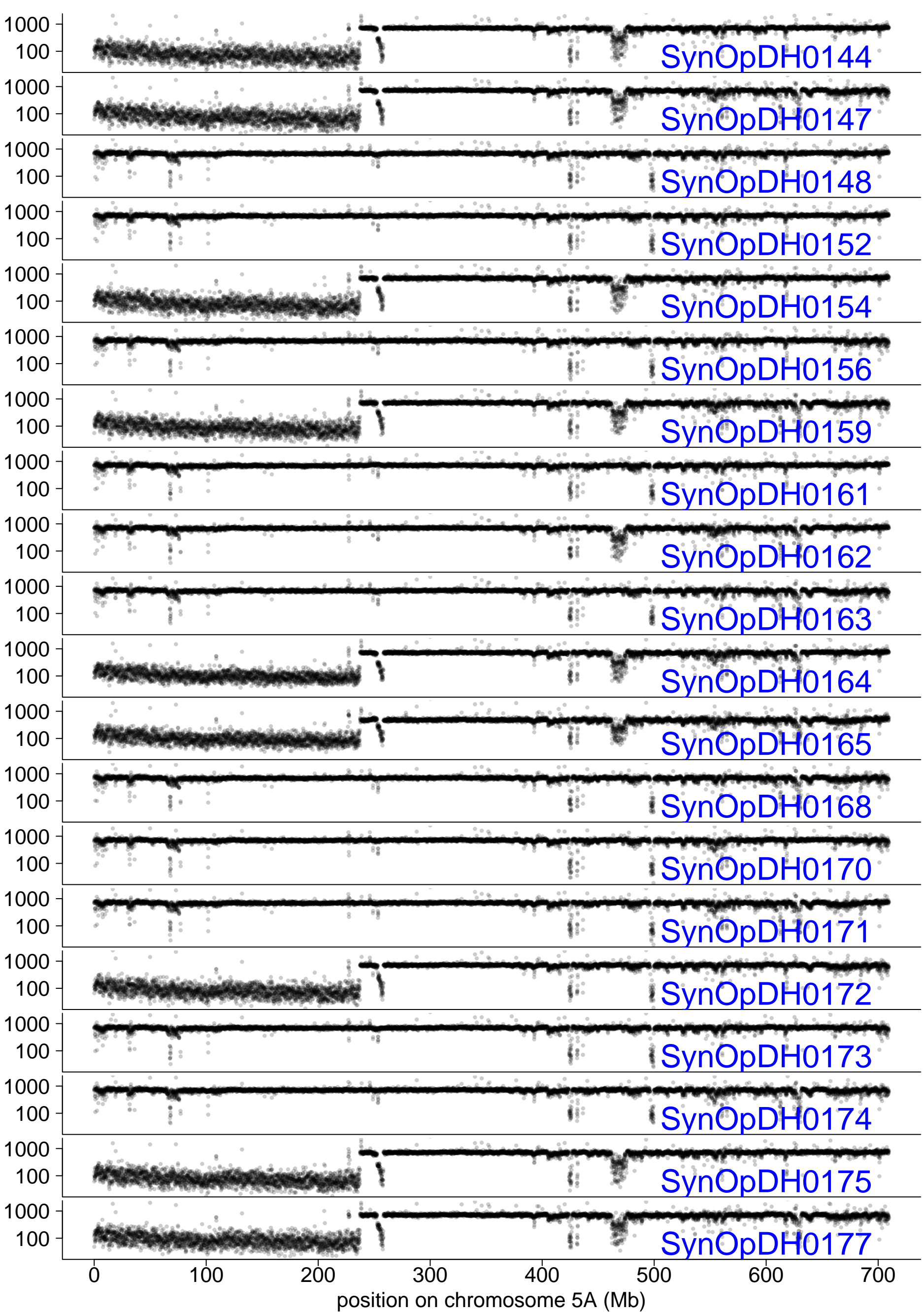

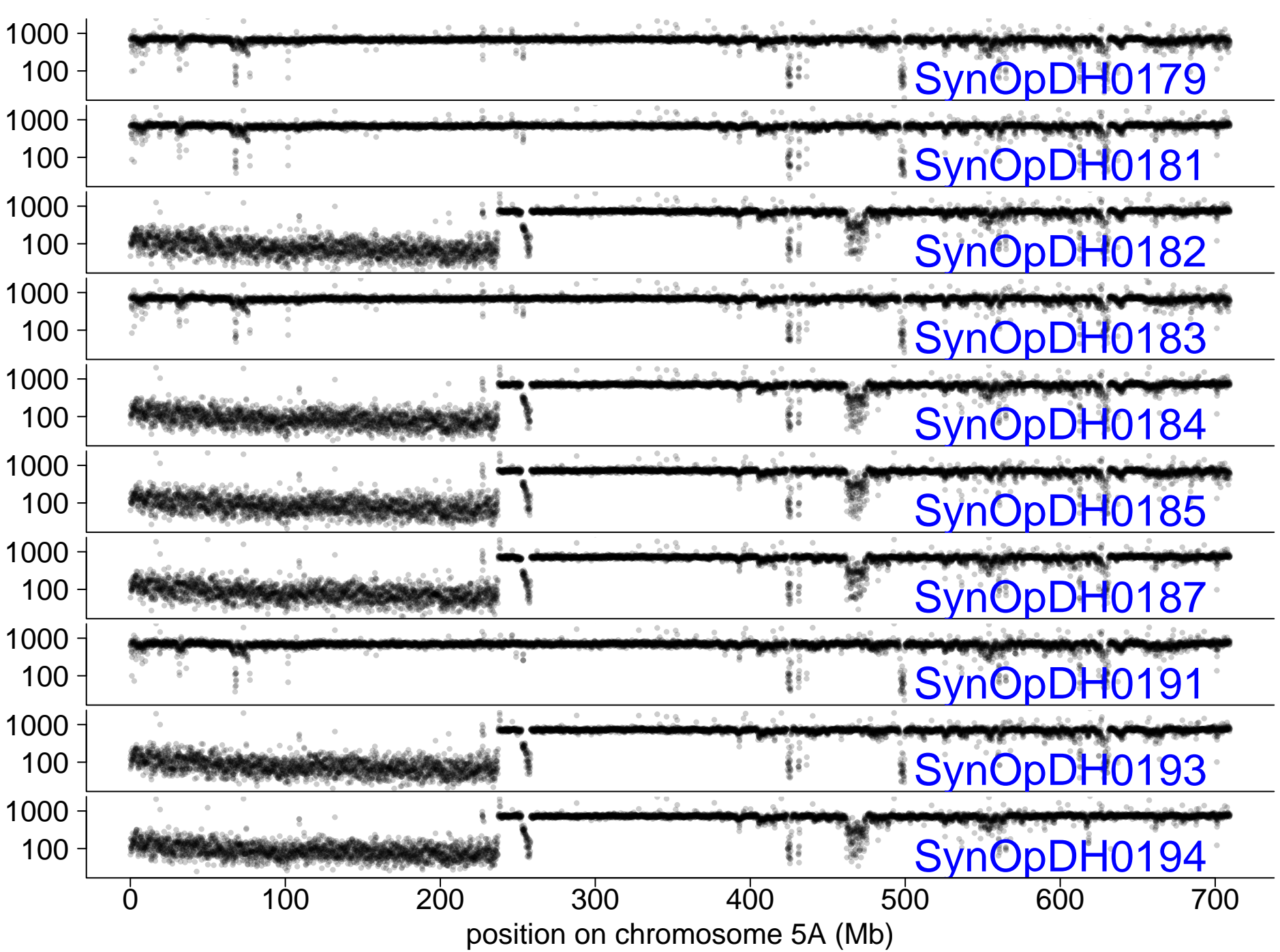

Supplement: Supplementary file 10 — Dataset S9 [file 41598_2018_38111_MOESM10_ESM.pdf]
