# Supplementary material for: Dense genotyping-by-sequencing linkage maps of two Synthetic W7984×Opata reference populations provide insights into wheat structural diversity
Source: Sci Rep. 2019 Feb 11;9:1793. doi: 10.1038/s41598-018-38111-3 (PMC6370774; doi:10.1038/s41598-018-38111-3)
Supplement: Supplementary file 13 — Dataset S12 [file 41598_2018_38111_MOESM13_ESM.pdf]

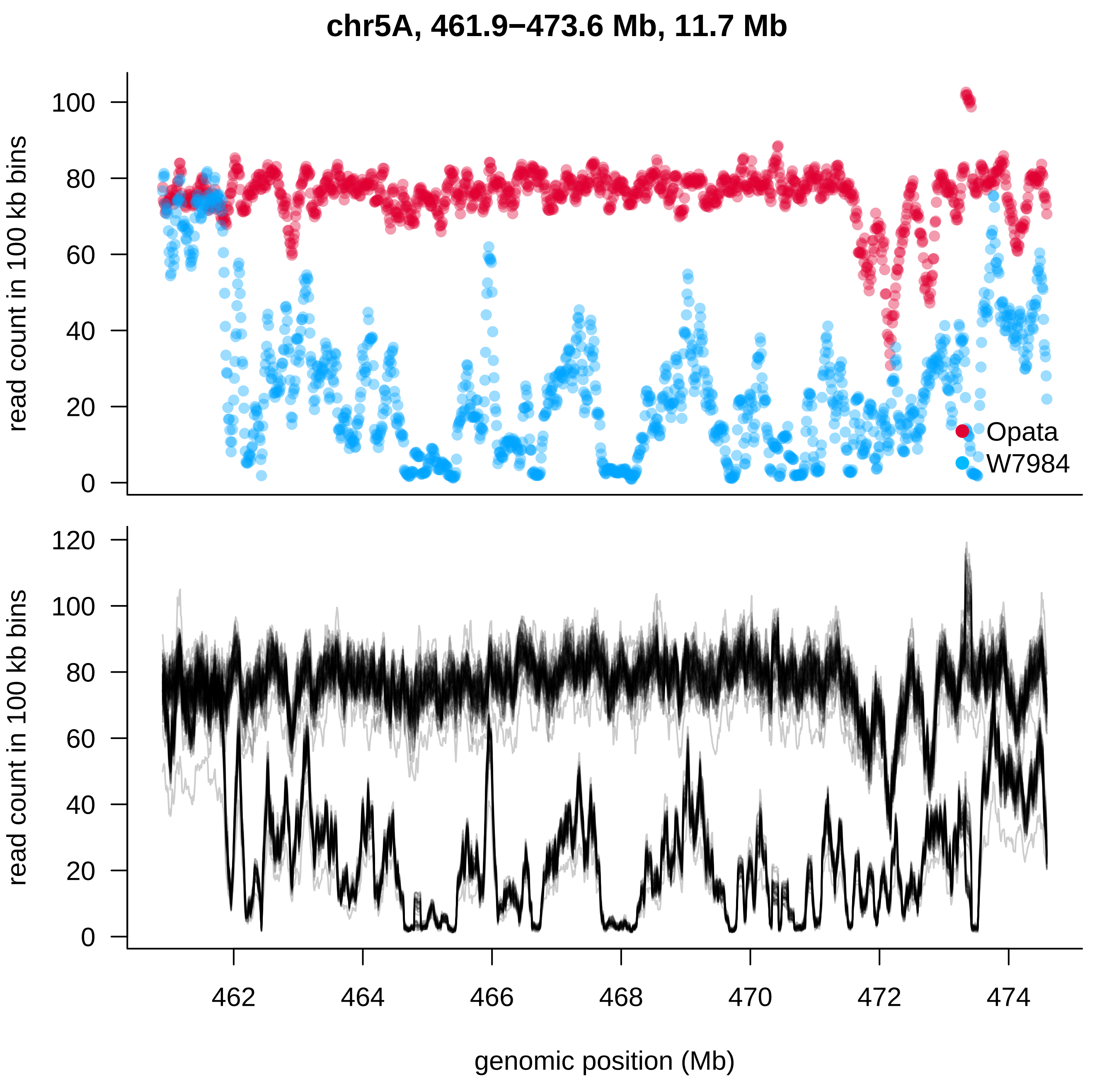
chr5A, 461.9−473.6 Mb, 11.7 Mb
read count in 100 kb bins
100
80
60
40
20
0
Opata
W7984
read count in 100 kb bins
120
100
80
60
40
20
0
462
464
466
468
470
472
474
genomic position (Mb)

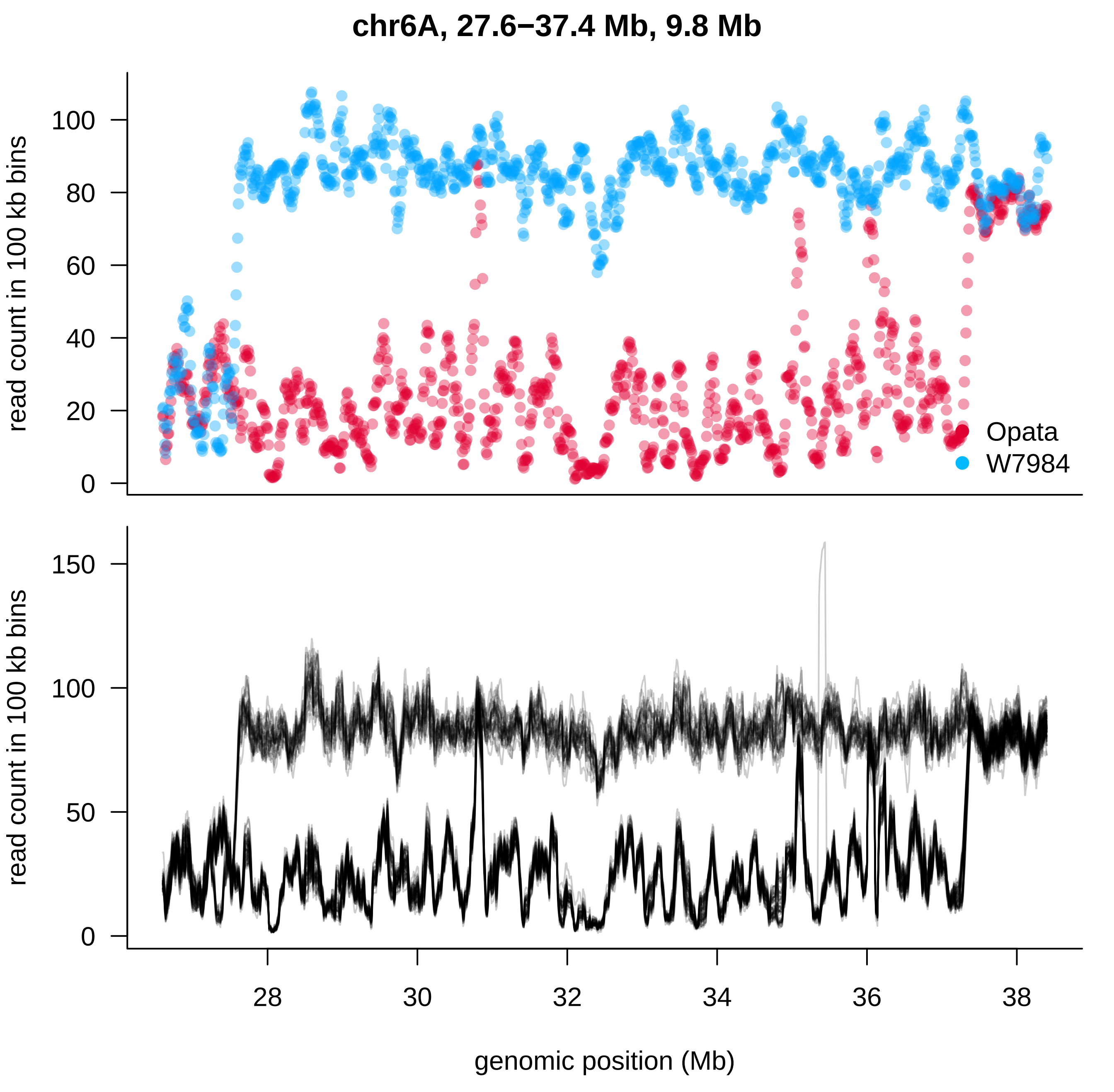

chr6A, 27.6−37.4 Mb, 9.8 Mb
read count in 100 kb bins
100
80
60
40
20
0
Opata
W7984
read count in 100 kb bins
150
100
50
0
28
30
32
34
36
38
genomic position (Mb)

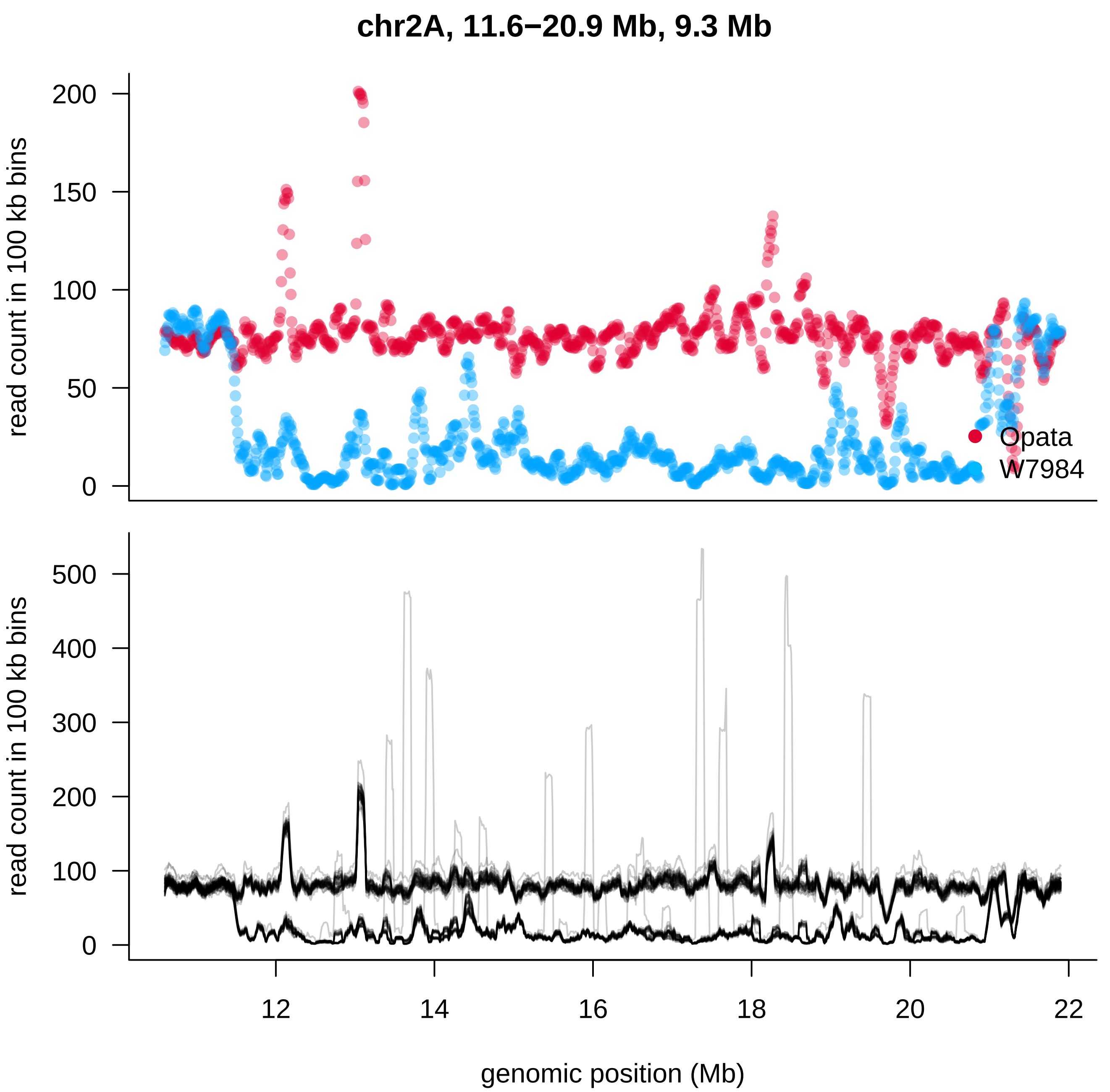

chr2A, 11.6−20.9 Mb, 9.3 Mb
read count in 100 kb bins
200
150
100
50
0
Opata
W7984
read count in 100 kb bins
500
400
300
200
100
0
12
14
16
18
20
22
genomic position (Mb)

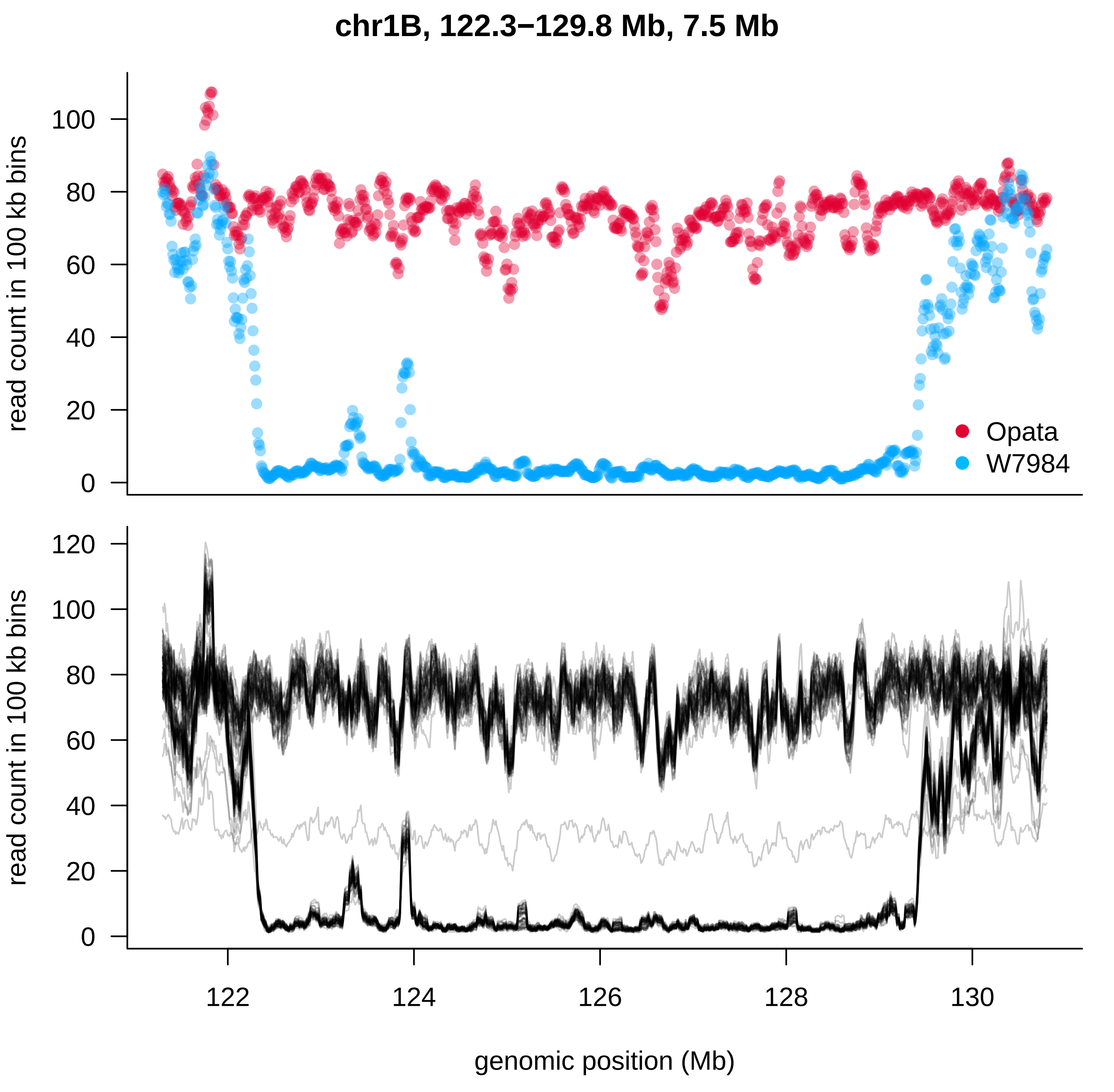

chr1B, 122.3−129.8 Mb, 7.5 Mb
read count in 100 kb bins
Opata
W7984
read count in 100 kb bins
genomic position (Mb)

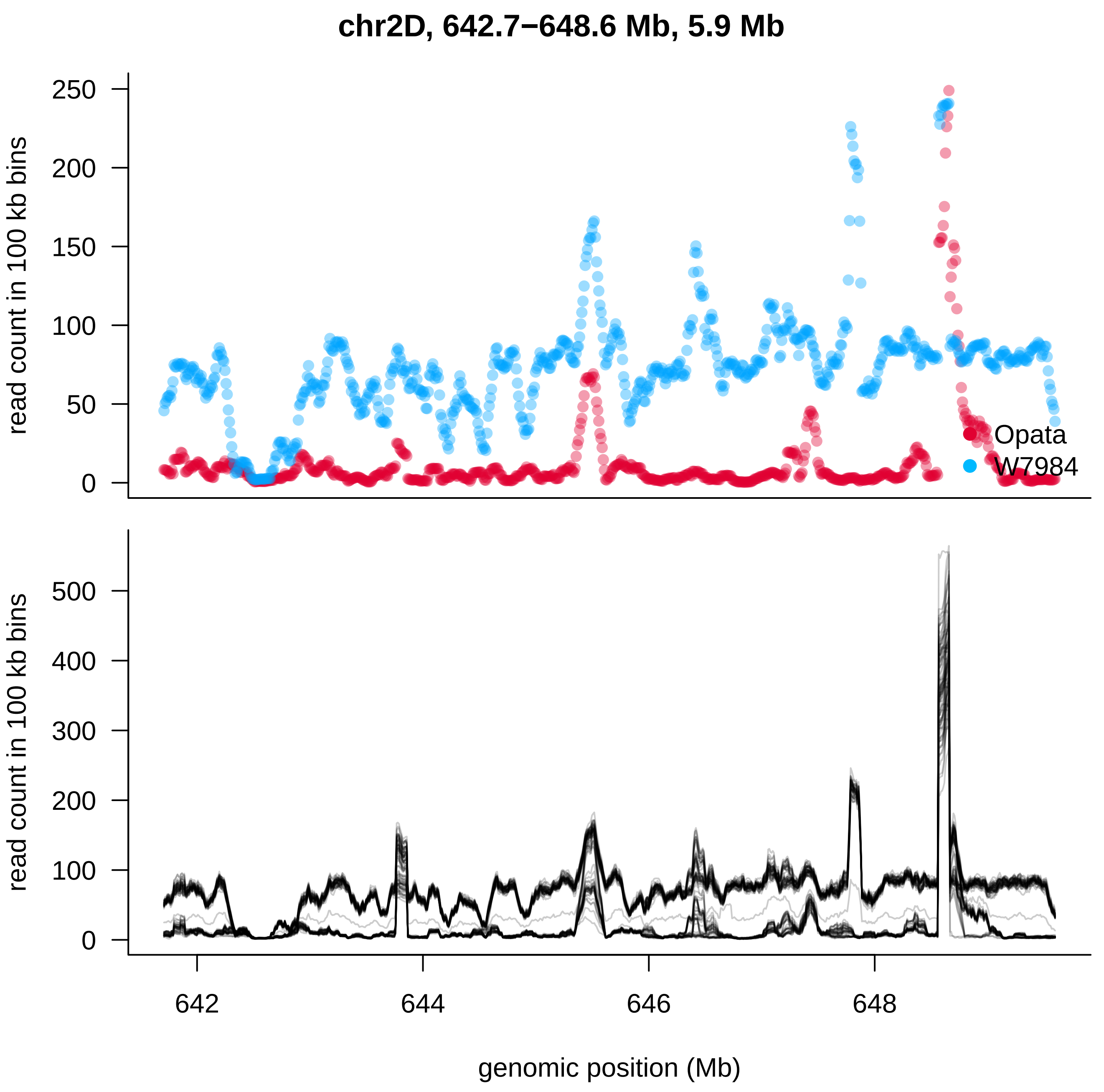
chr2D, 642.7−648.6 Mb, 5.9 Mb
read count in 100 kb bins
250
200
150
100
50
0
Opata
W7984
read count in 100 kb bins
500
400
300
200
100
0
642
644
646
648
genomic position (Mb)

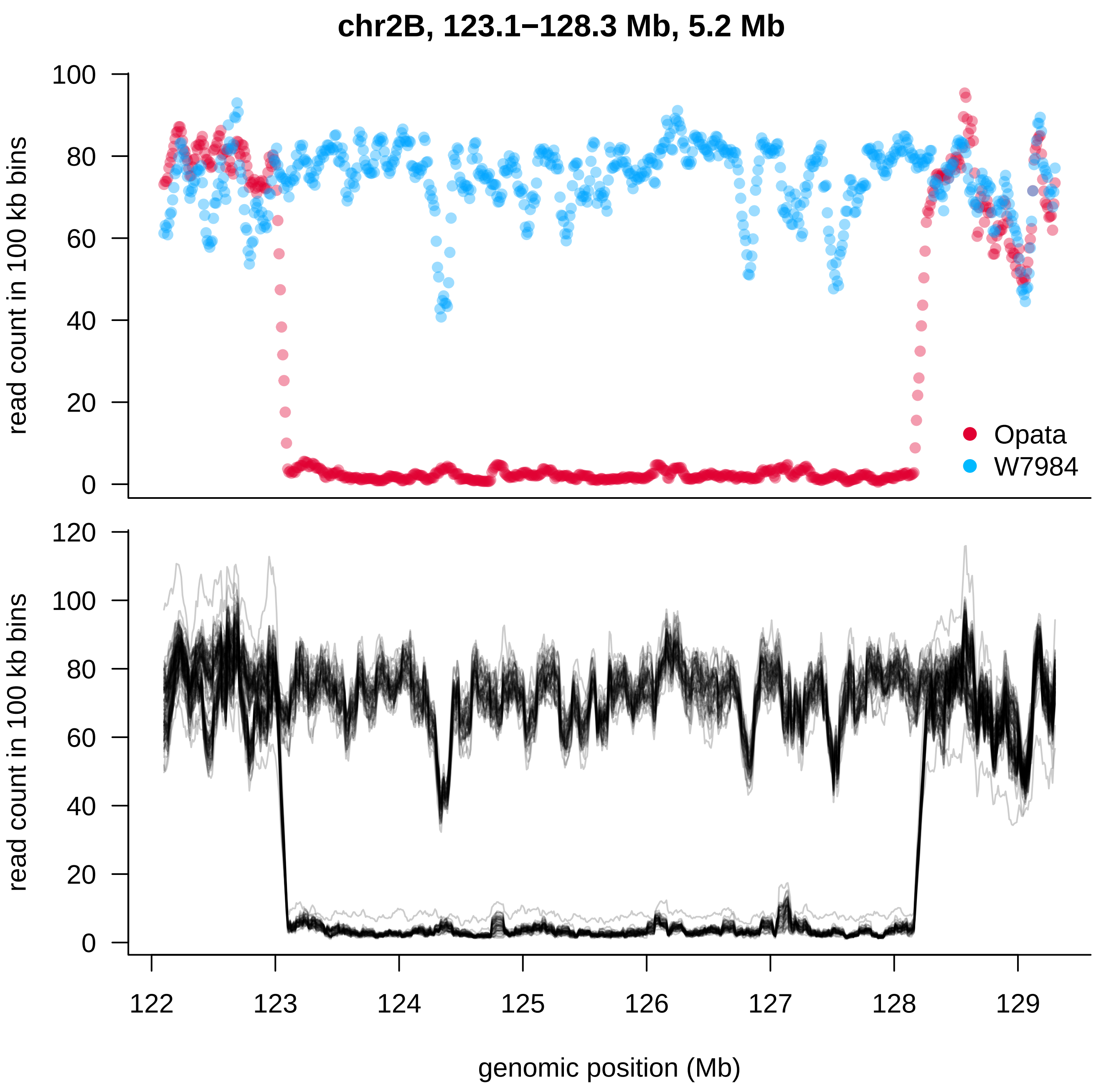
chr2B, 123.1−128.3 Mb, 5.2 Mb
read count in 100 kb bins
100
80
60
40
20
0
Opata
W7984
read count in 100 kb bins
120
100
80
60
40
20
0
122
123
124
125
126
127
128
129
genomic position (Mb)

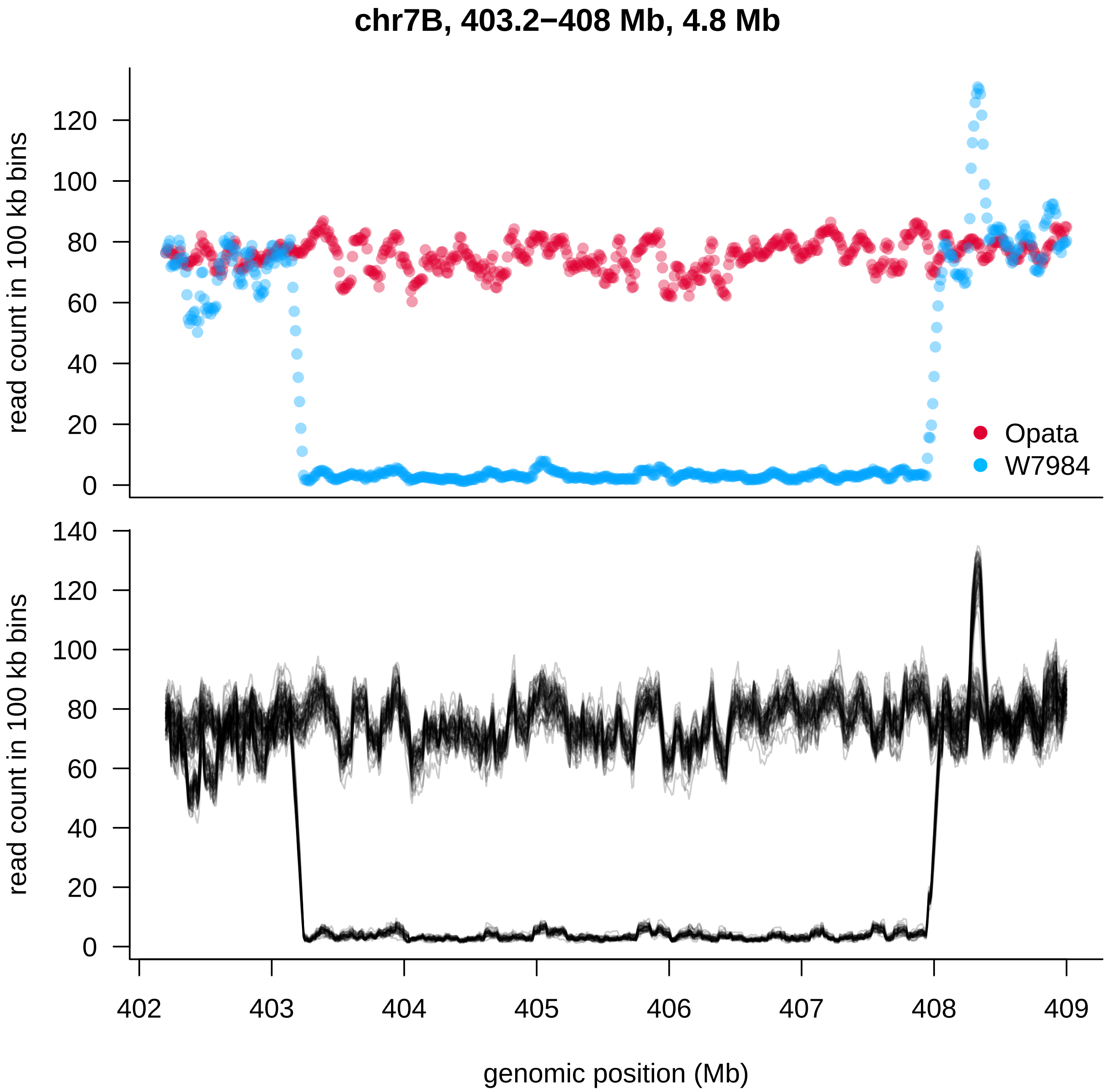

chr7B, 403.2−408 Mb, 4.8 Mb
read count in 100 kb bins
Opata
W7984
read count in 100 kb bins
genomic position (Mb)

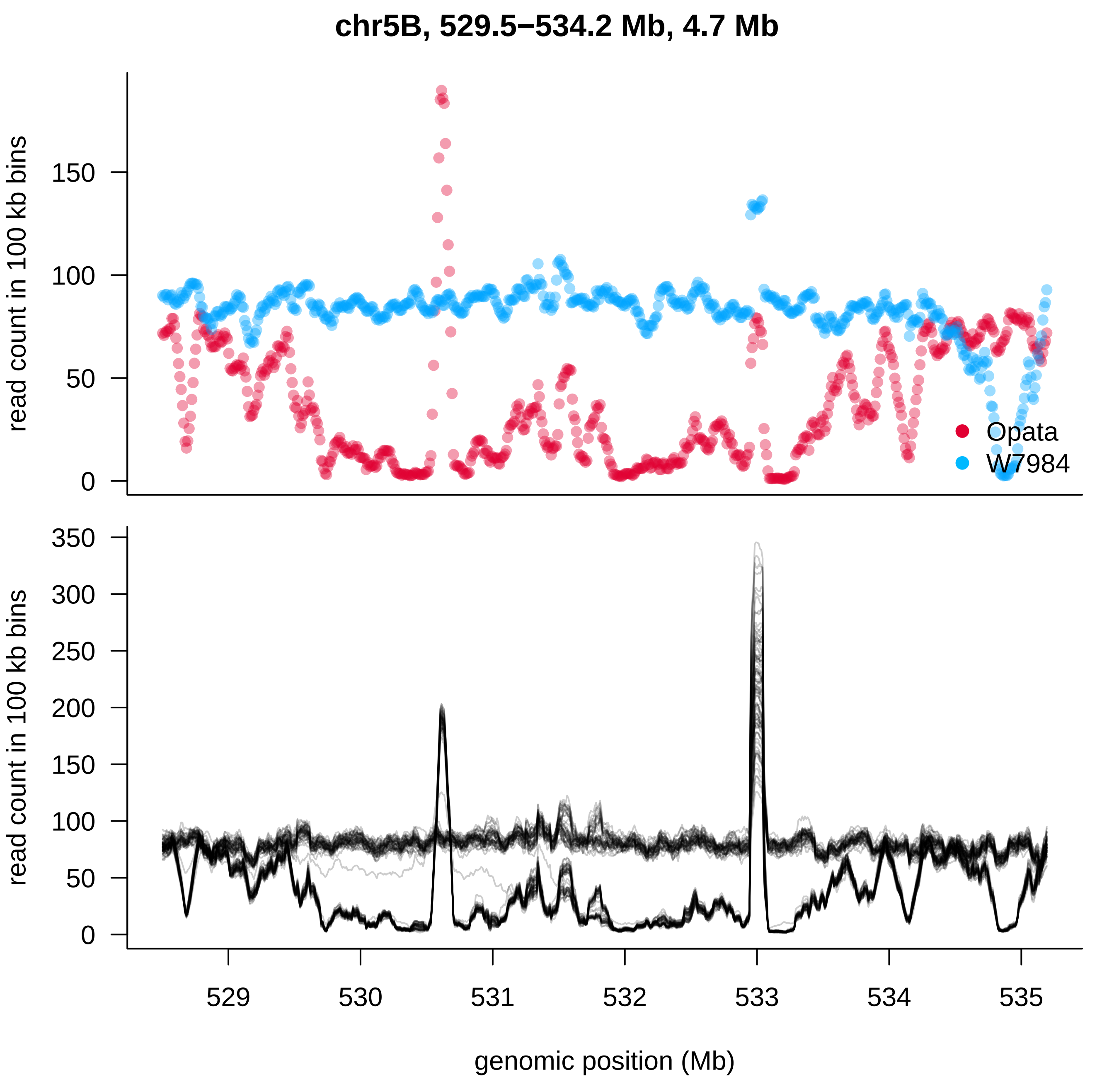
chr5B, 529.5–534.2 Mb, 4.7 Mb
read count in 100 kb bins
150
100
50
0
Opata
W7984
read count in 100 kb bins
350
300
250
200
150
100
50
0
529
530
531
532
533
534
535
genomic position (Mb)

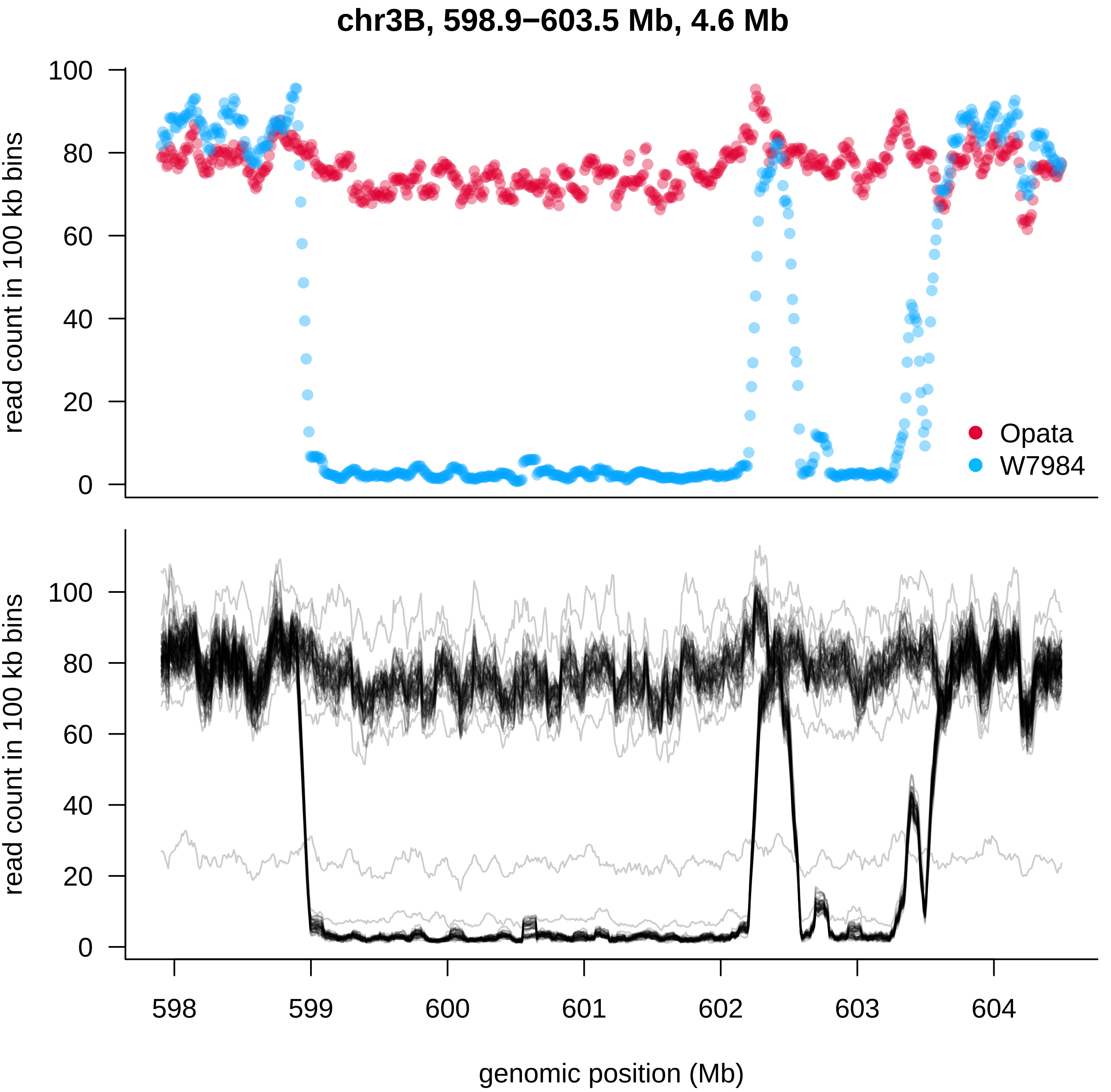

chr3B, 598.9−603.5 Mb, 4.6 Mb
read count in 100 kb bins
100
80
60
40
20
0
Opata
W7984
read count in 100 kb bins
100
80
60
40
20
0
598
599
600
601
602
603
604
genomic position (Mb)

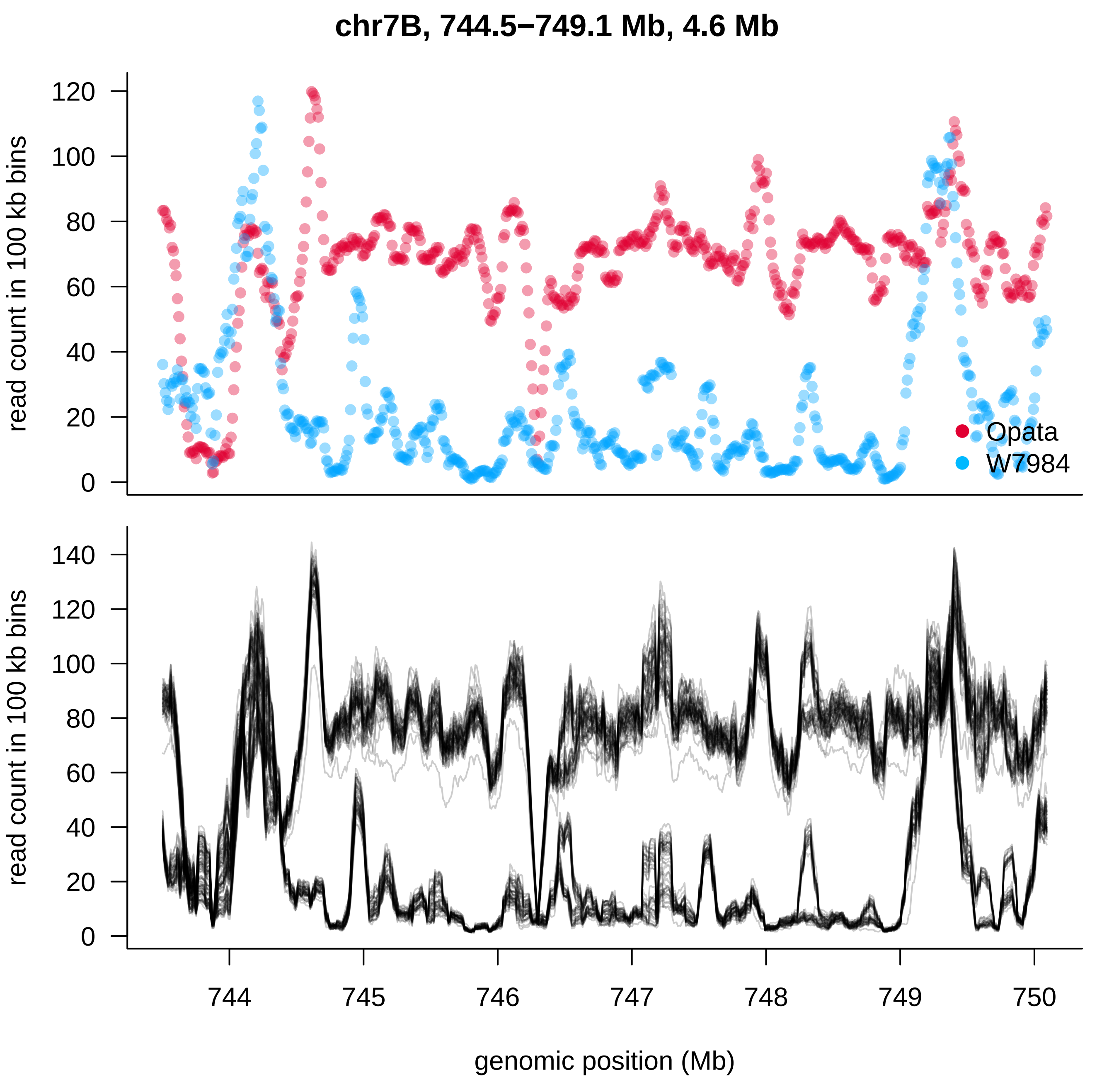

chr7B, 744.5−749.1 Mb, 4.6 Mb
read count in 100 kb bins
Opata
W7984
read count in 100 kb bins
genomic position (Mb)

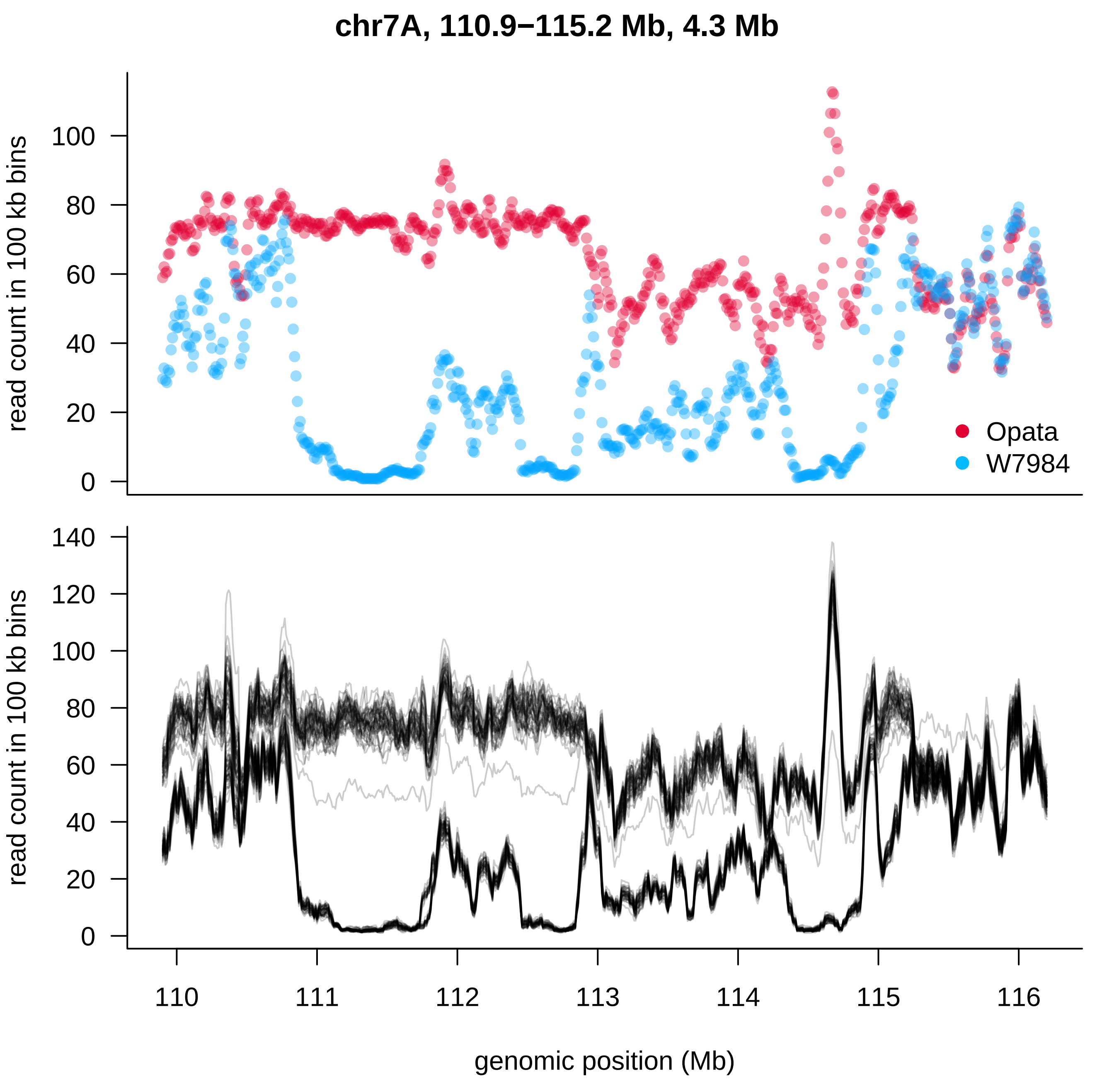

chr7A, 110.9−115.2 Mb, 4.3 Mb
read count in 100 kb bins
0
20
40
60
80
100
Opata
W7984
read count in 100 kb bins
0
20
40
60
80
100
120
140
110
111
112
113
114
115
116
genomic position (Mb)

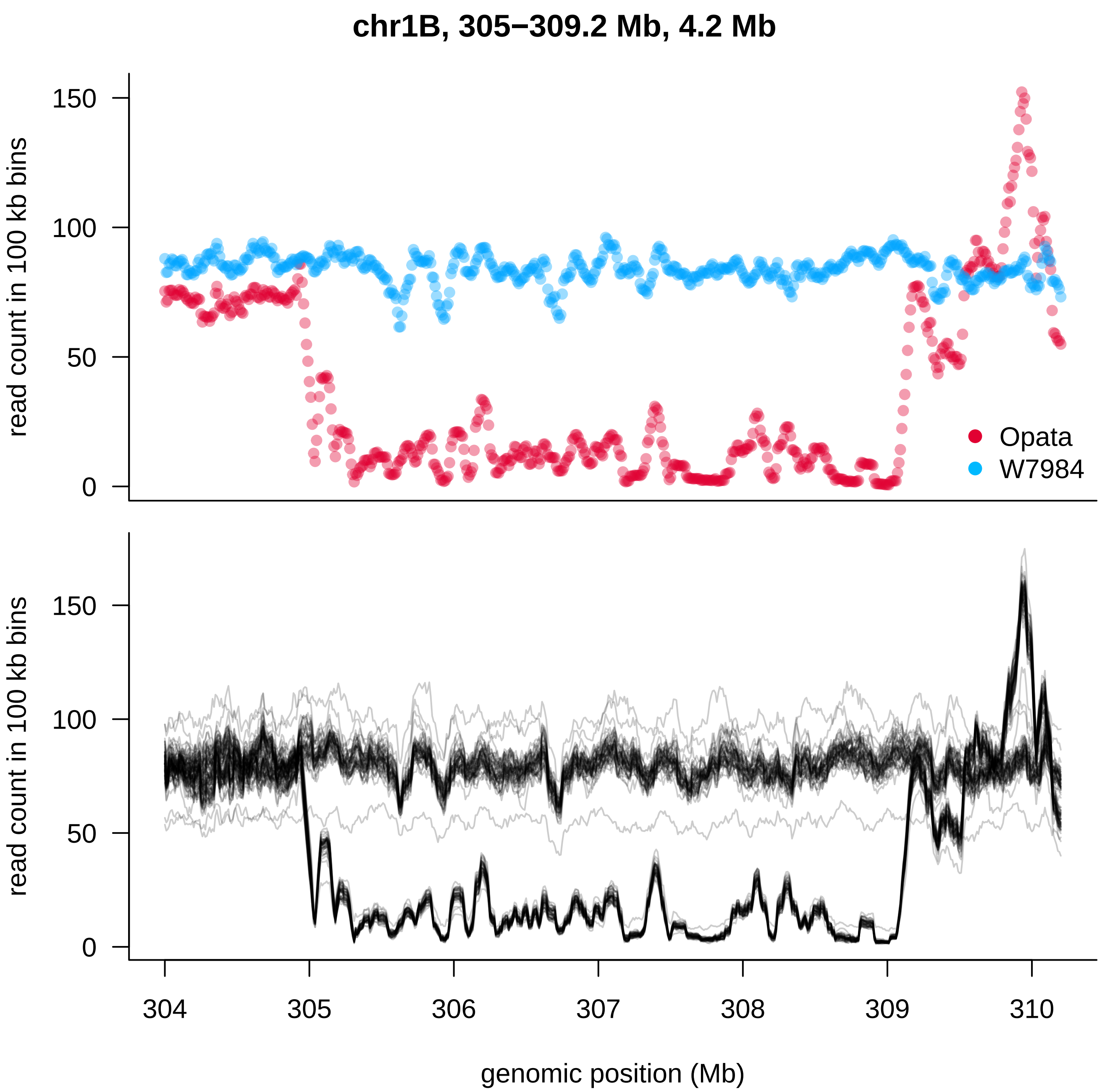
chr1B, 305−309.2 Mb, 4.2 Mb
read count in 100 kb bins
150
100
50
0
Opata
W7984
read count in 100 kb bins
150
100
50
0
304
305
306
307
308
309
310
genomic position (Mb)

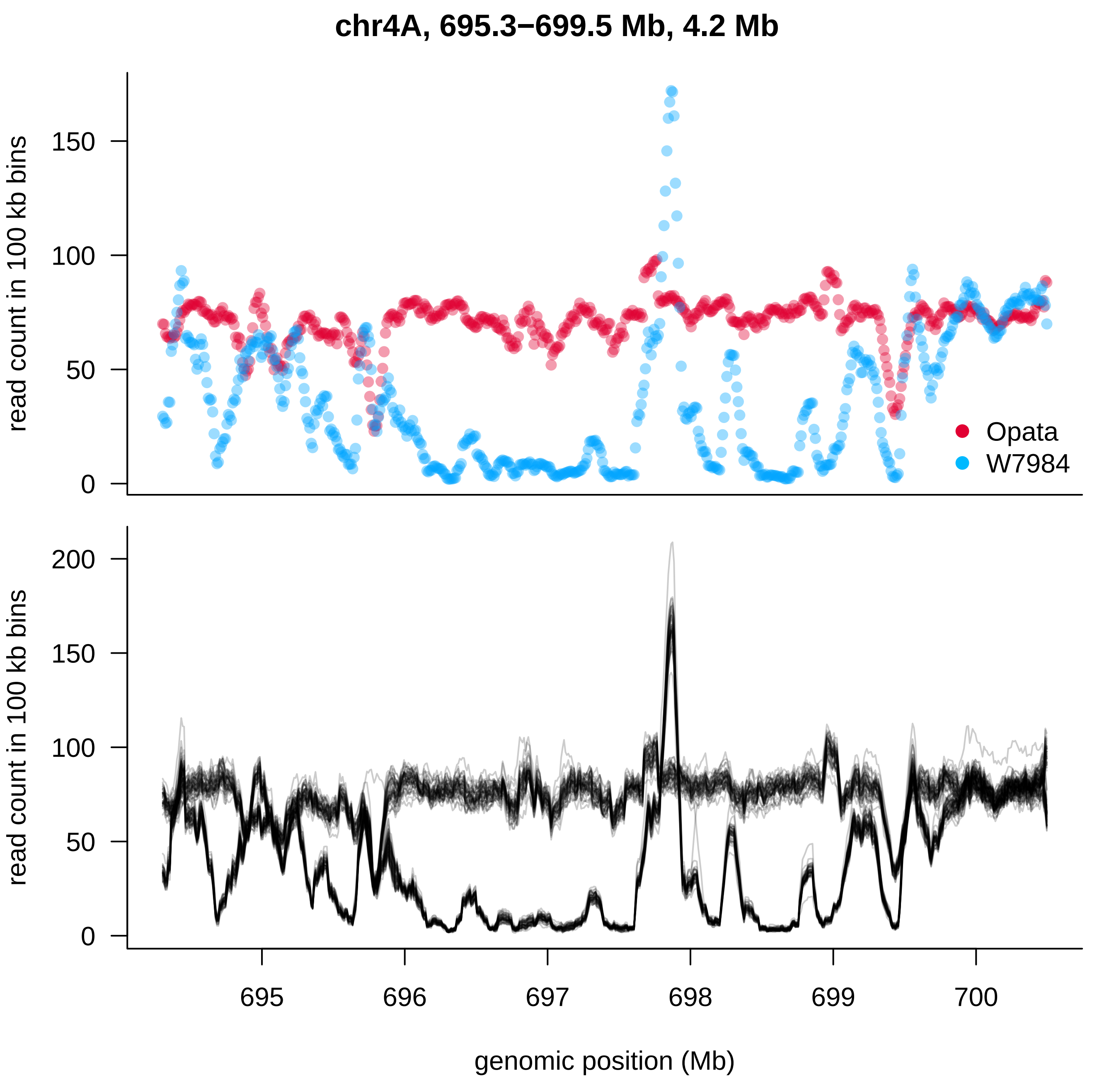
chr4A, 695.3−699.5 Mb, 4.2 Mb
read count in 100 kb bins
0
50
100
150
Opata
W7984
read count in 100 kb bins
0
50
100
150
200
695
696
697
698
699
700
genomic position (Mb)

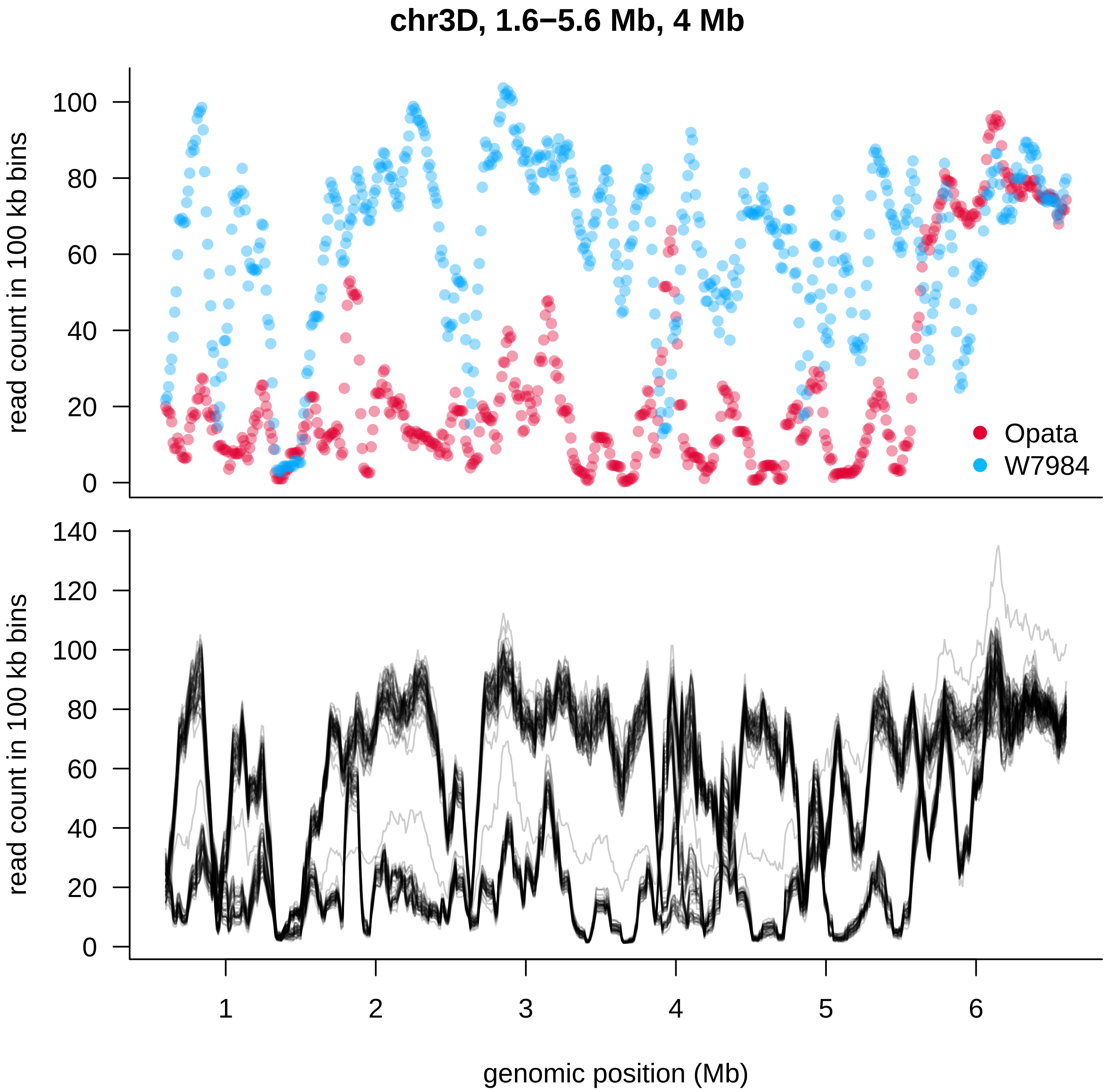
chr3D, 1.6−5.6 Mb, 4 Mb
read count in 100 kb bins
0
20
40
60
80
100
Opata
W7984
read count in 100 kb bins
0
20
40
60
80
100
120
140
1
2
3
4
5
6
genomic position (Mb)

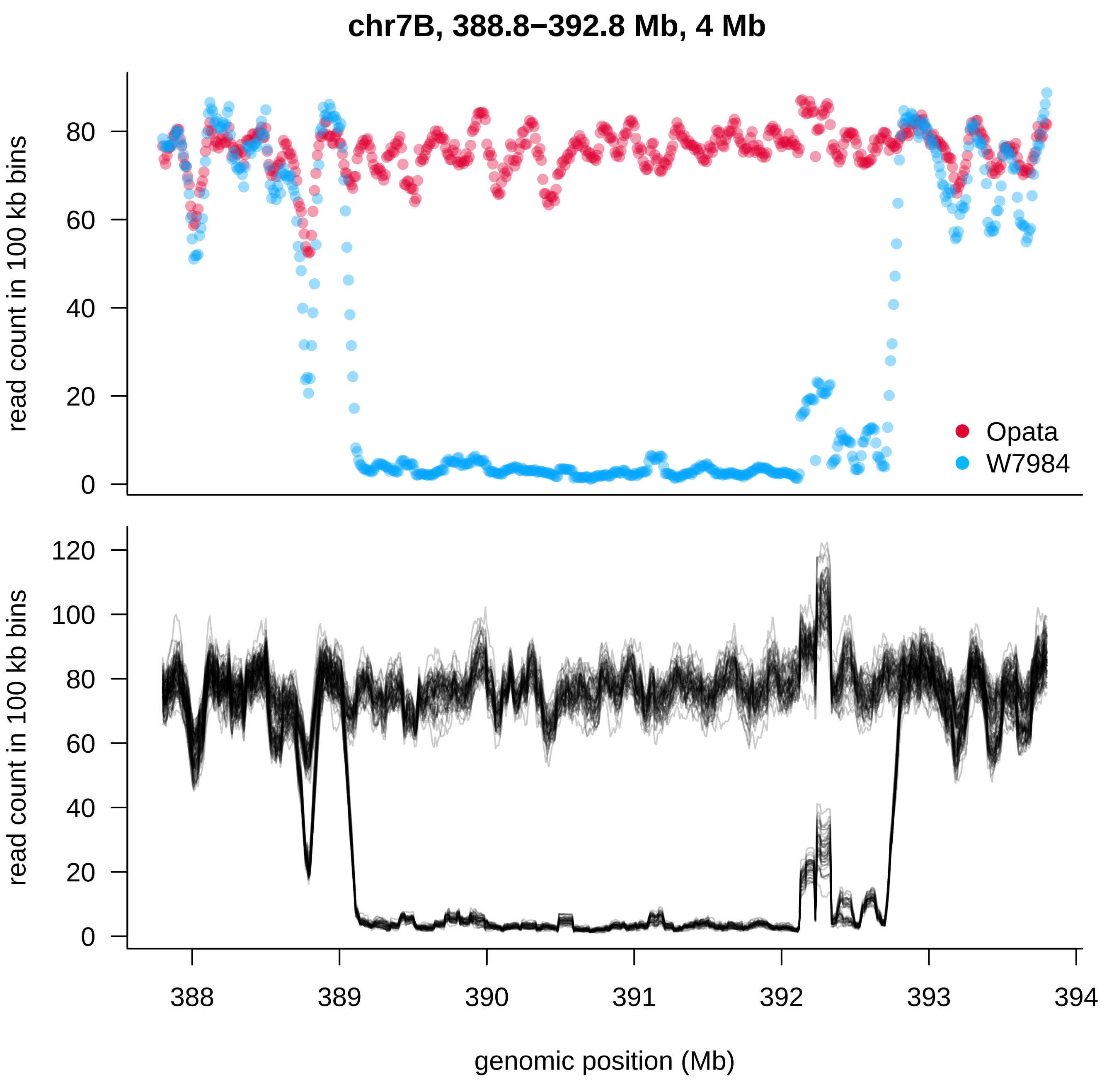
chr7B, 388.8−392.8 Mb, 4 Mb
read count in 100 kb bins
0
20
40
60
80
Opata
W7984
read count in 100 kb bins
0
20
40
60
80
100
120
388
389
390
391
392
393
394
genomic position (Mb)

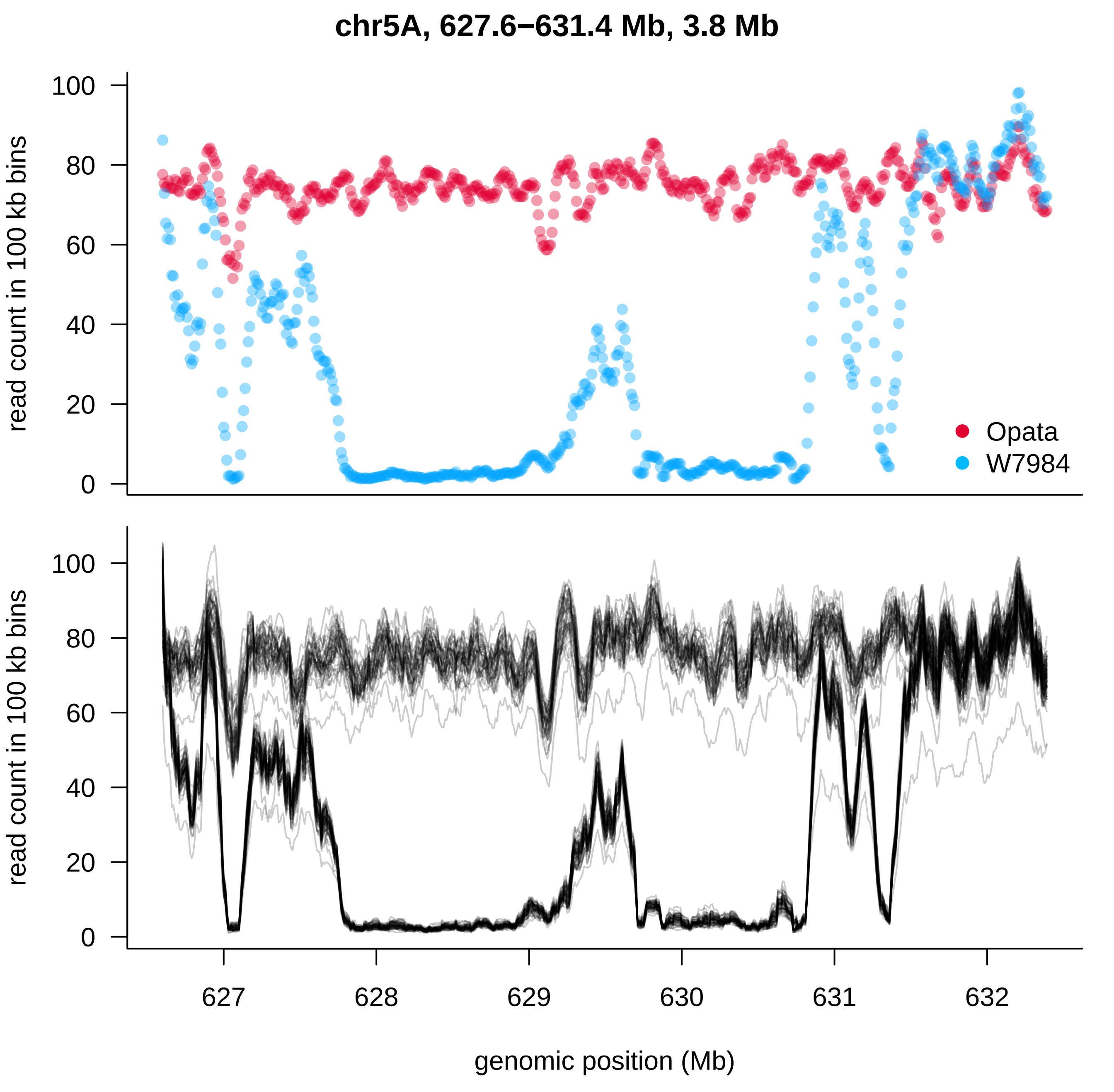
chr5A, 627.6−631.4 Mb, 3.8 Mb
read count in 100 kb bins
Opata
W7984
read count in 100 kb bins
genomic position (Mb)

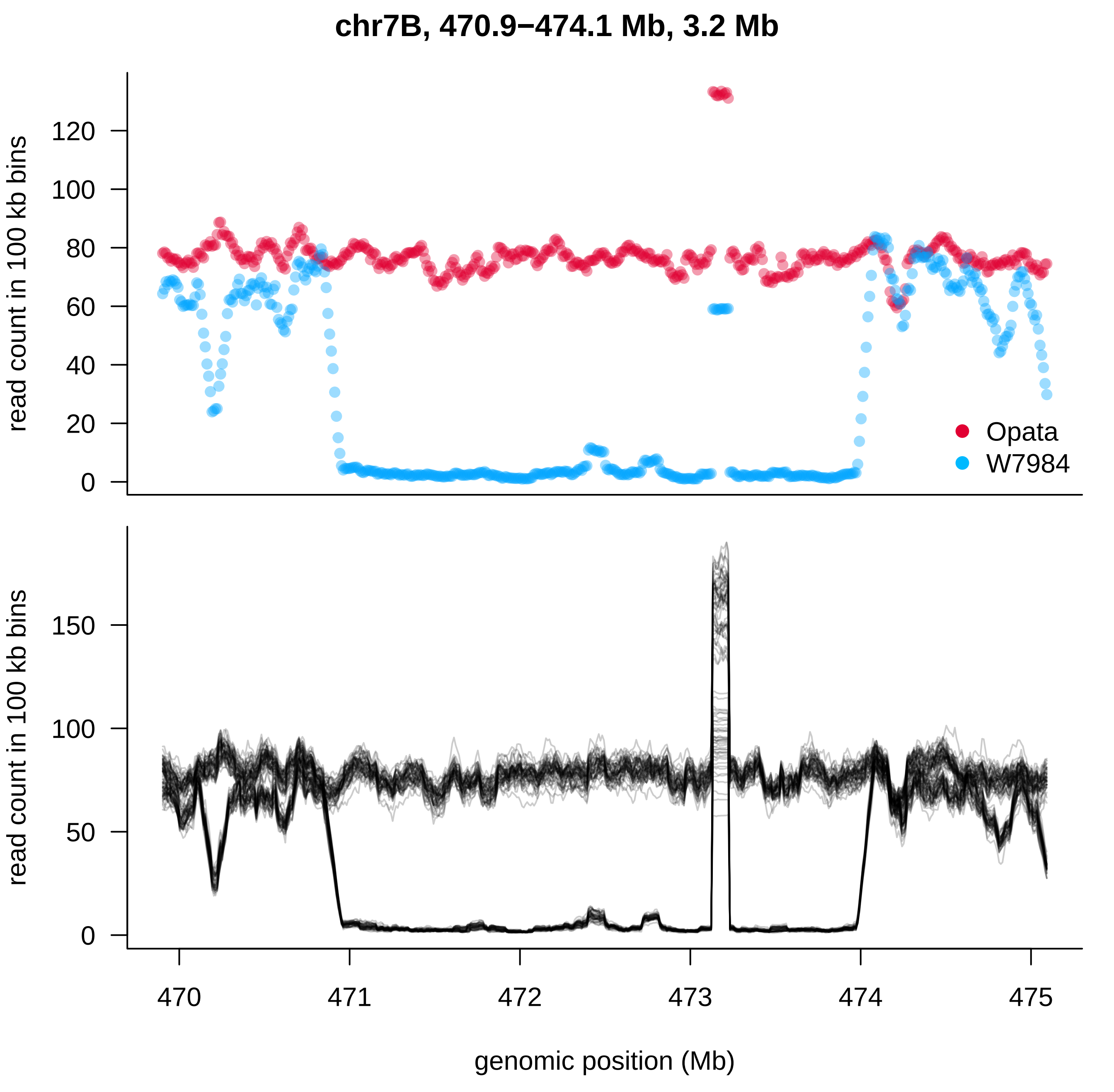
chr7B, 470.9−474.1 Mb, 3.2 Mb
read count in 100 kb bins
0
20
40
60
80
100
120
Opata
W7984
read count in 100 kb bins
0
50
100
150
470
471
472
473
474
475
genomic position (Mb)

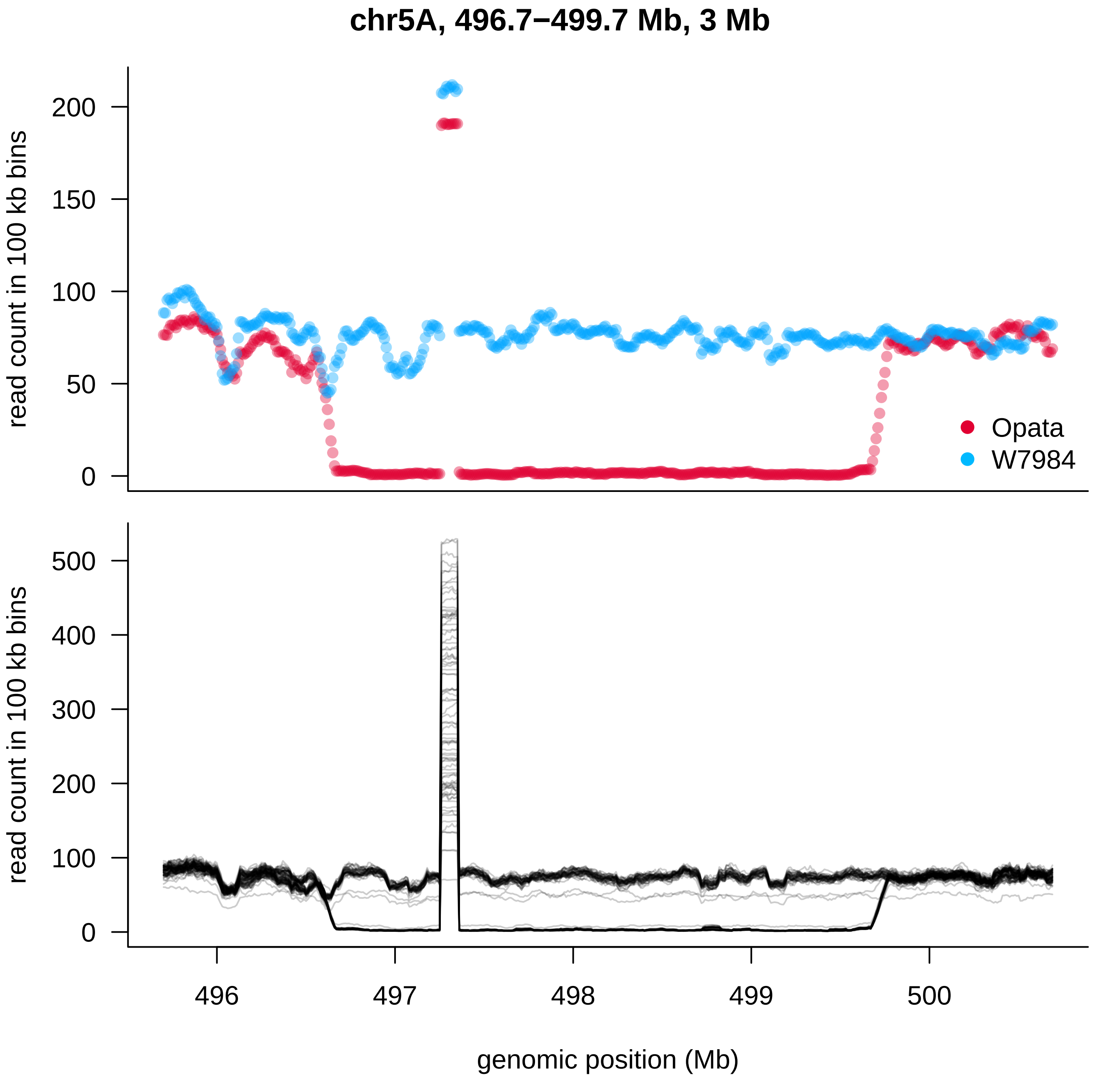
chr5A, 496.7−499.7 Mb, 3 Mb
read count in 100 kb bins
0
50
100
150
200
Opata
W7984
read count in 100 kb bins
0
100
200
300
400
500
496
497
498
499
500
genomic position (Mb)

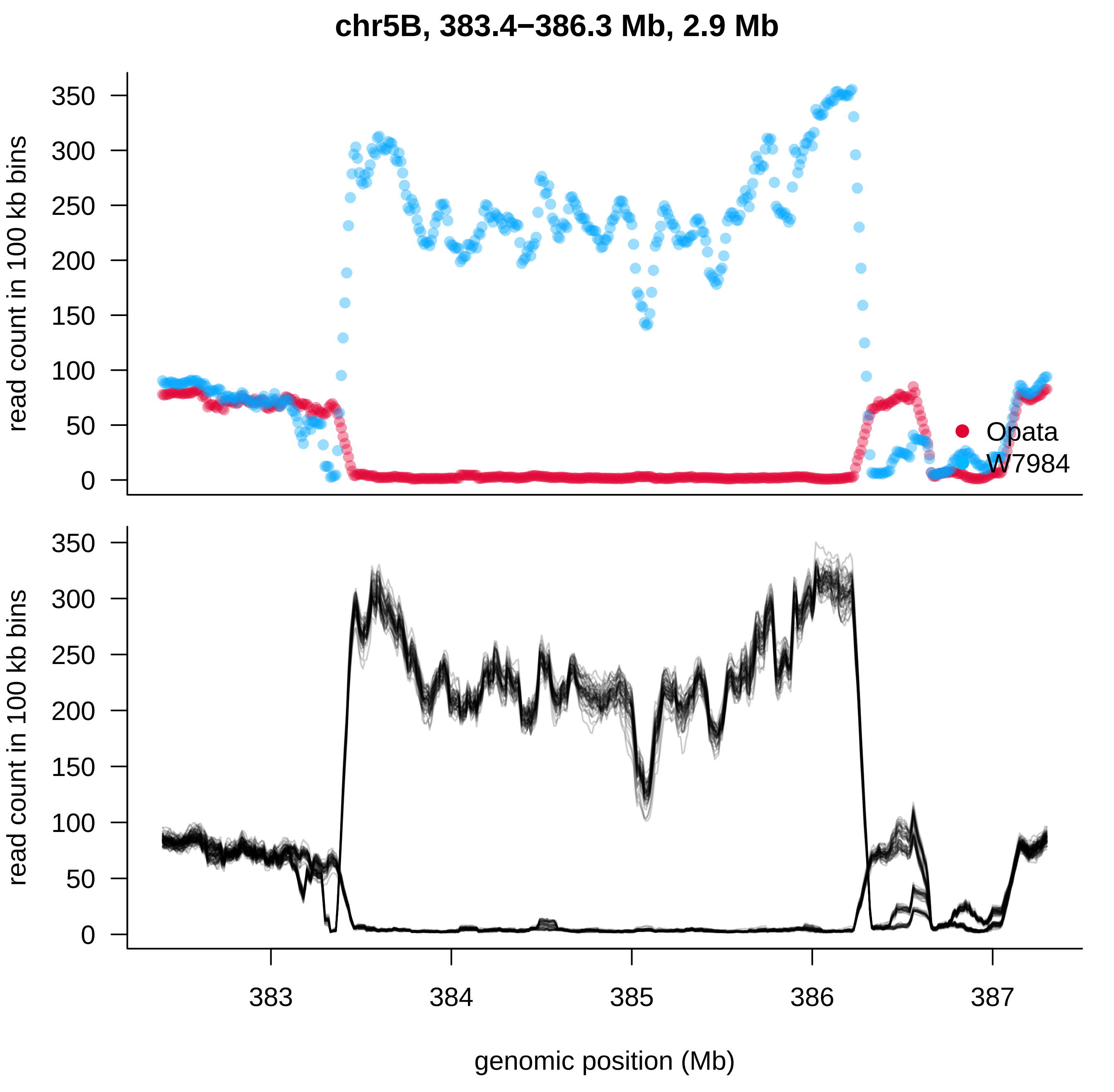
chr5B, 383.4−386.3 Mb, 2.9 Mb
read count in 100 kb bins
350
300
250
200
150
100
50
0
Opata
W7984
read count in 100 kb bins
350
300
250
200
150
100
50
0
383
384
385
386
387
genomic position (Mb)

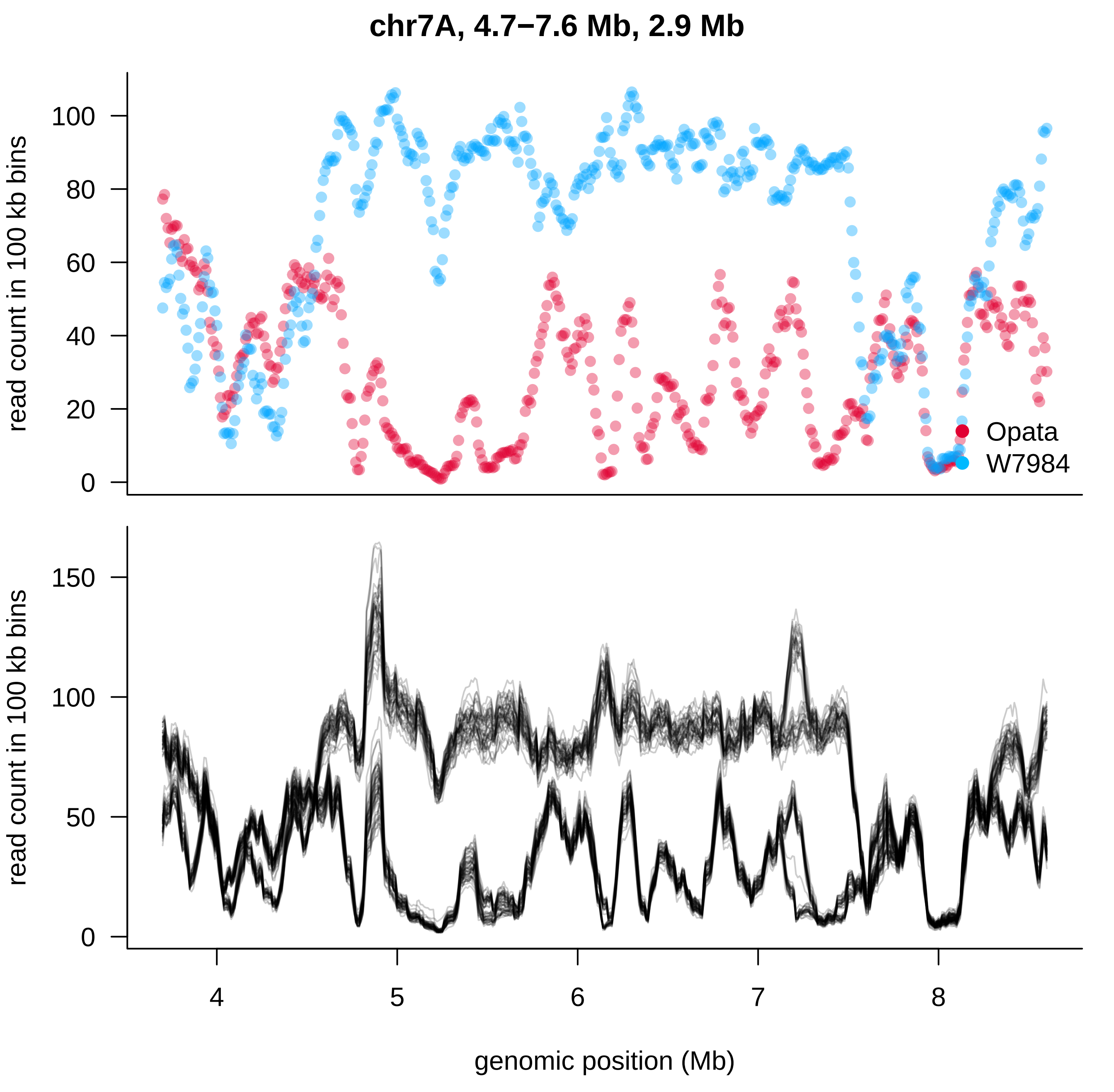

chr7A, 4.7−7.6 Mb, 2.9 Mb
read count in 100 kb bins
0
20
40
60
80
100
Opata
W7984
read count in 100 kb bins
0
50
100
150
4
5
6
7
8
genomic position (Mb)

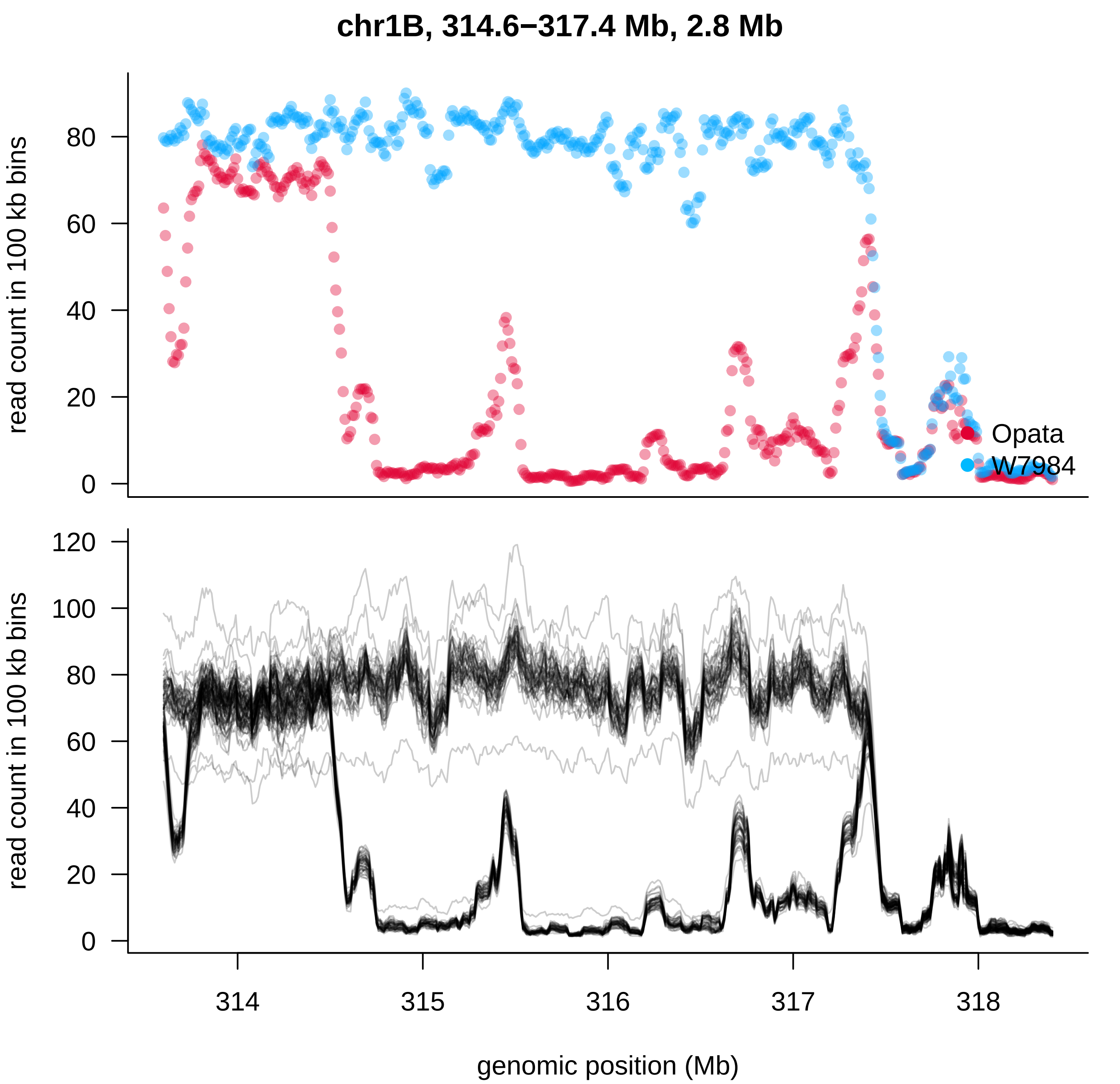
chr1B, 314.6−317.4 Mb, 2.8 Mb
read count in 100 kb bins
0
20
40
60
80
Opata
W7984
read count in 100 kb bins
0
20
40
60
80
100
120
314
315
316
317
318
genomic position (Mb)

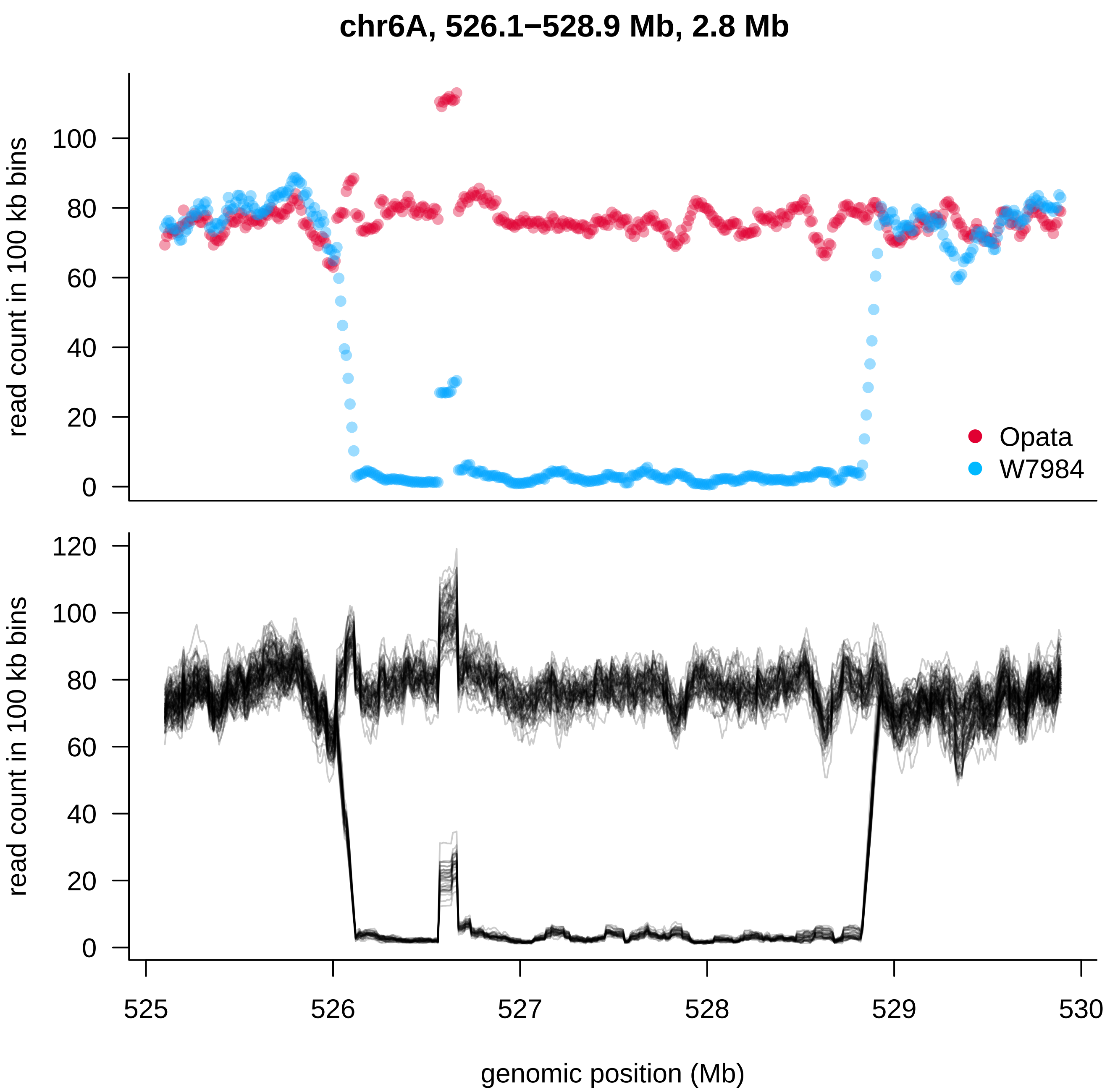
chr6A, 526.1−528.9 Mb, 2.8 Mb
read count in 100 kb bins
100
80
60
40
20
0
Opata
W7984
read count in 100 kb bins
120
100
80
60
40
20
0
525
526
527
528
529
530
genomic position (Mb)

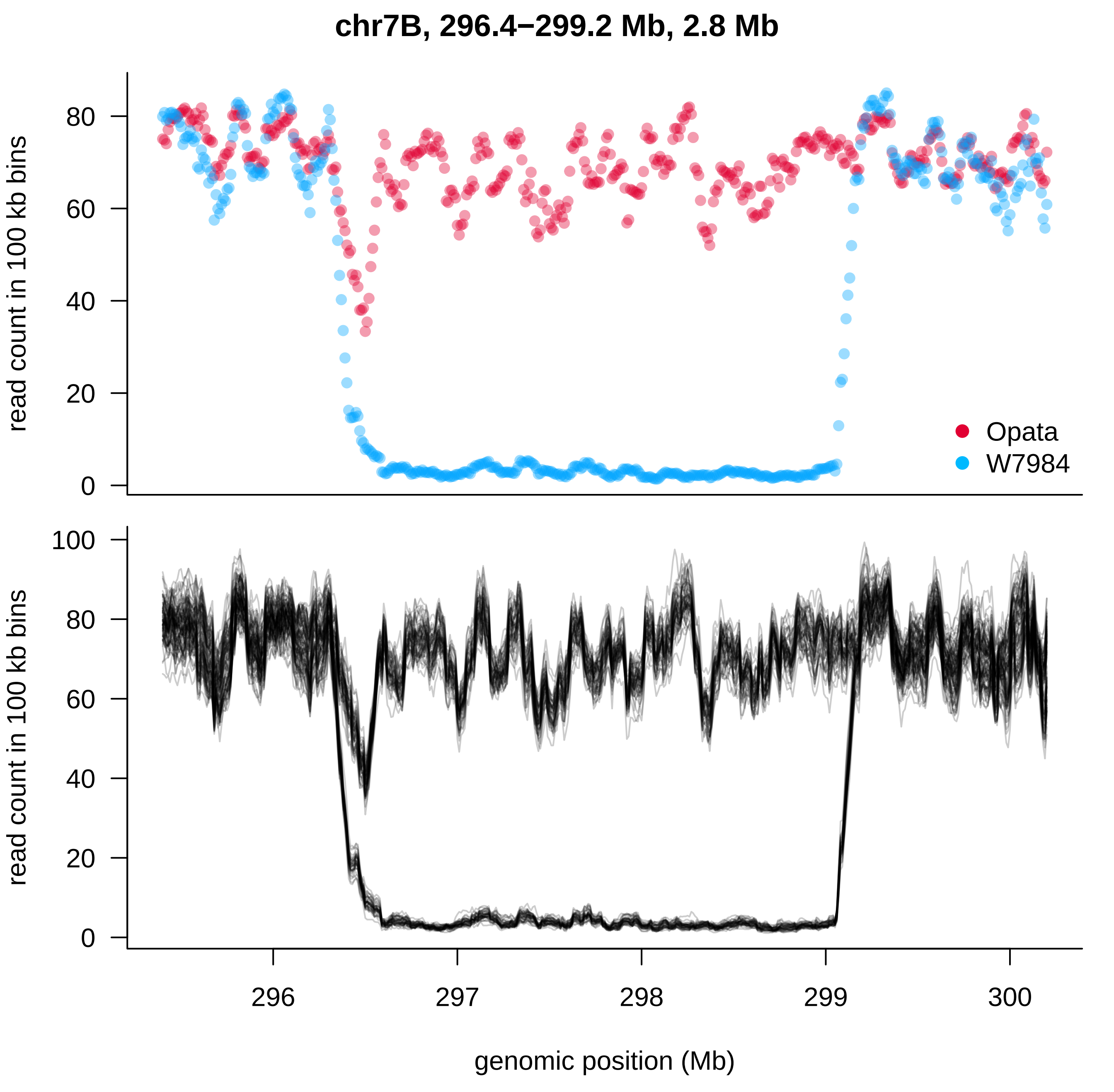

chr7B, 296.4−299.2 Mb, 2.8 Mb
read count in 100 kb bins
0
20
40
60
80
Opata
W7984
read count in 100 kb bins
0
20
40
60
80
100
296
297
298
299
300
genomic position (Mb)

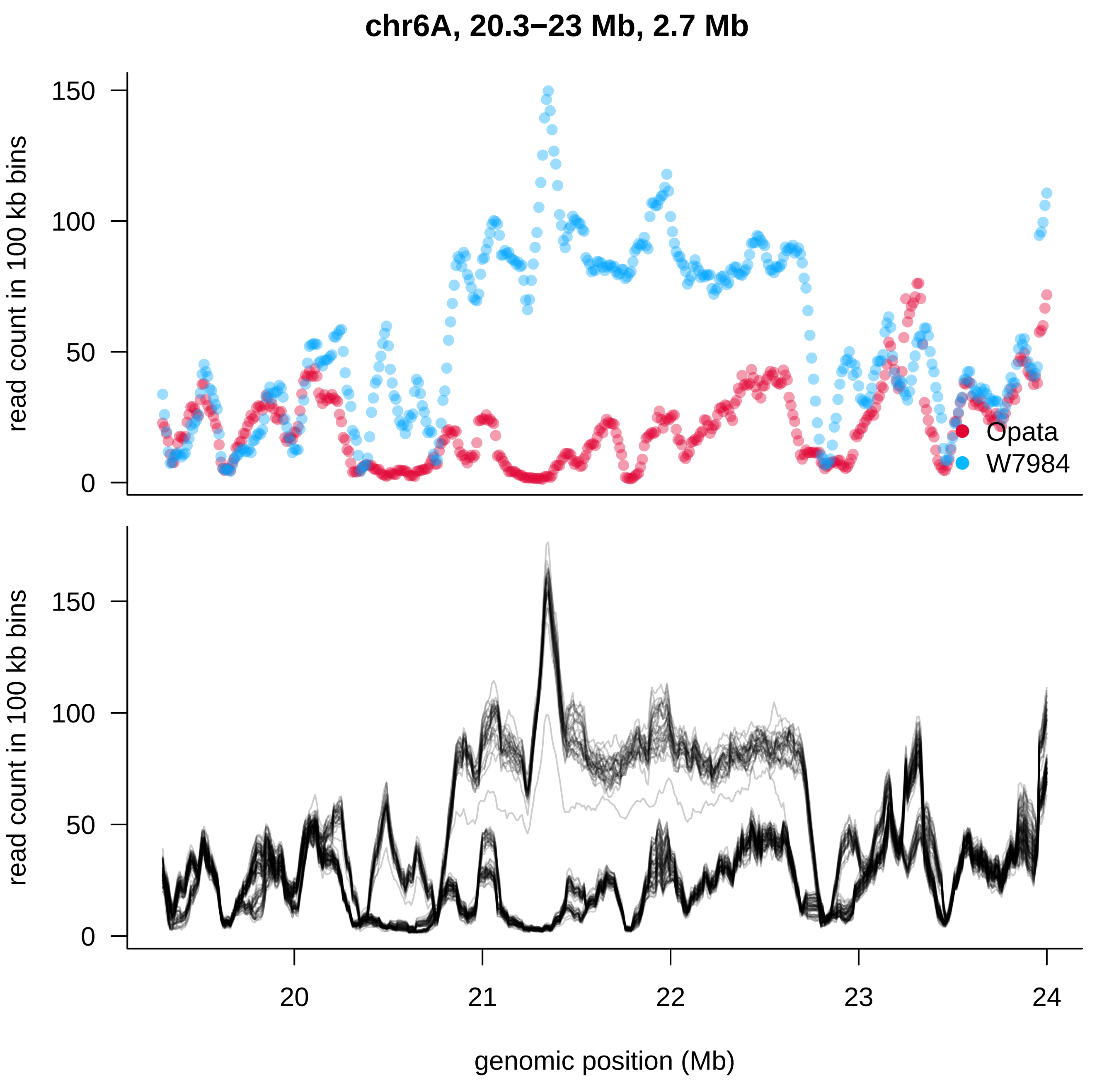

chr6A, 20.3−23 Mb, 2.7 Mb
read count in 100 kb bins
150
100
50
0
Opata
W7984
read count in 100 kb bins
150
100
50
0
20
21
22
23
24
genomic position (Mb)

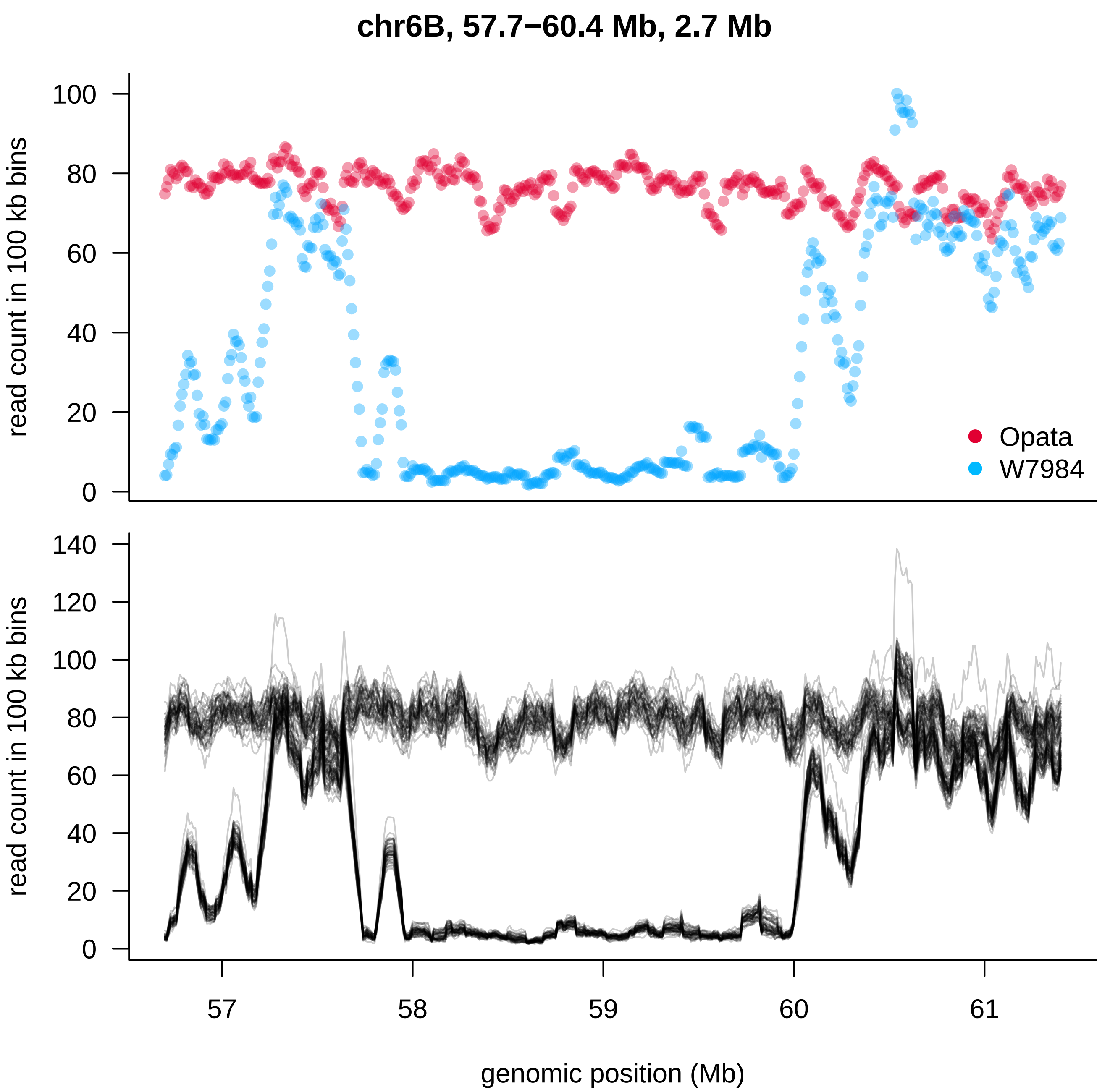
chr6B, 57.7−60.4 Mb, 2.7 Mb
read count in 100 kb bins
Opata
W7984
read count in 100 kb bins
genomic position (Mb)

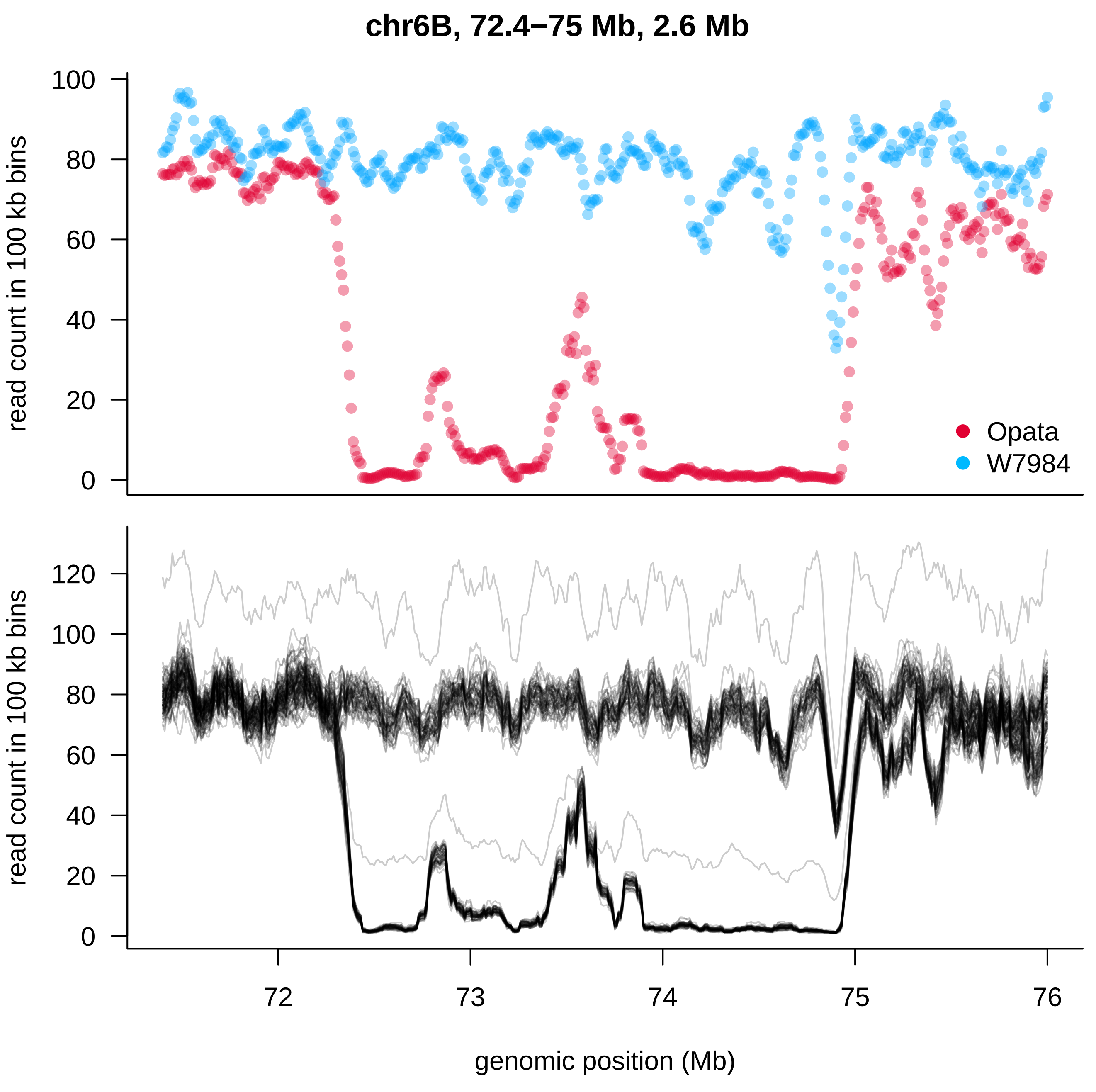
chr6B, 72.4−75 Mb, 2.6 Mb
read count in 100 kb bins
0
20
40
60
80
100
Opata
W7984
read count in 100 kb bins
0
20
40
60
80
100
120
72
73
74
75
76
genomic position (Mb)

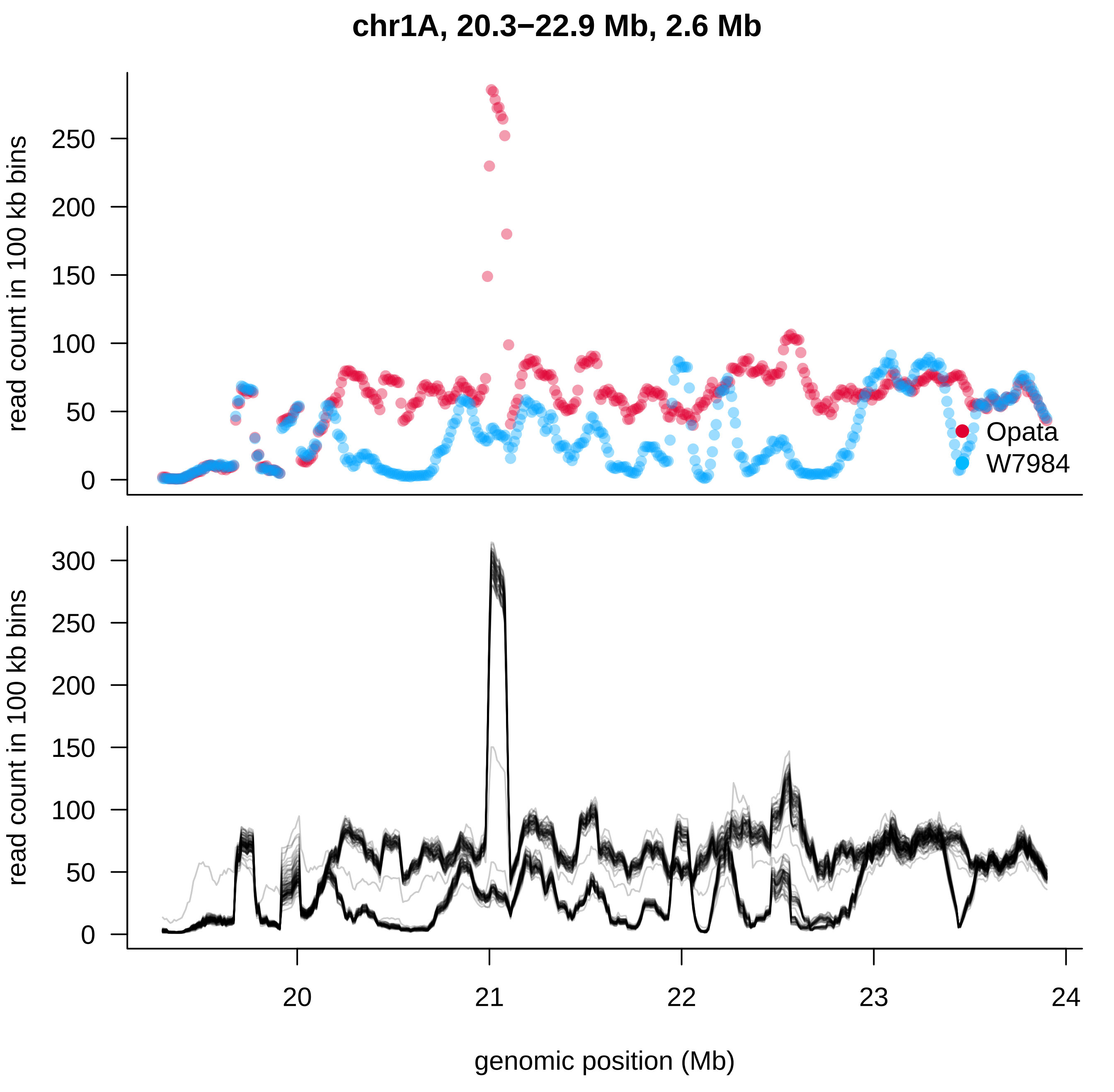
chr1A, 20.3−22.9 Mb, 2.6 Mb
read count in 100 kb bins
0
50
100
150
200
250
Opata
W7984
read count in 100 kb bins
0
50
100
150
200
250
300
20
21
22
23
24
genomic position (Mb)

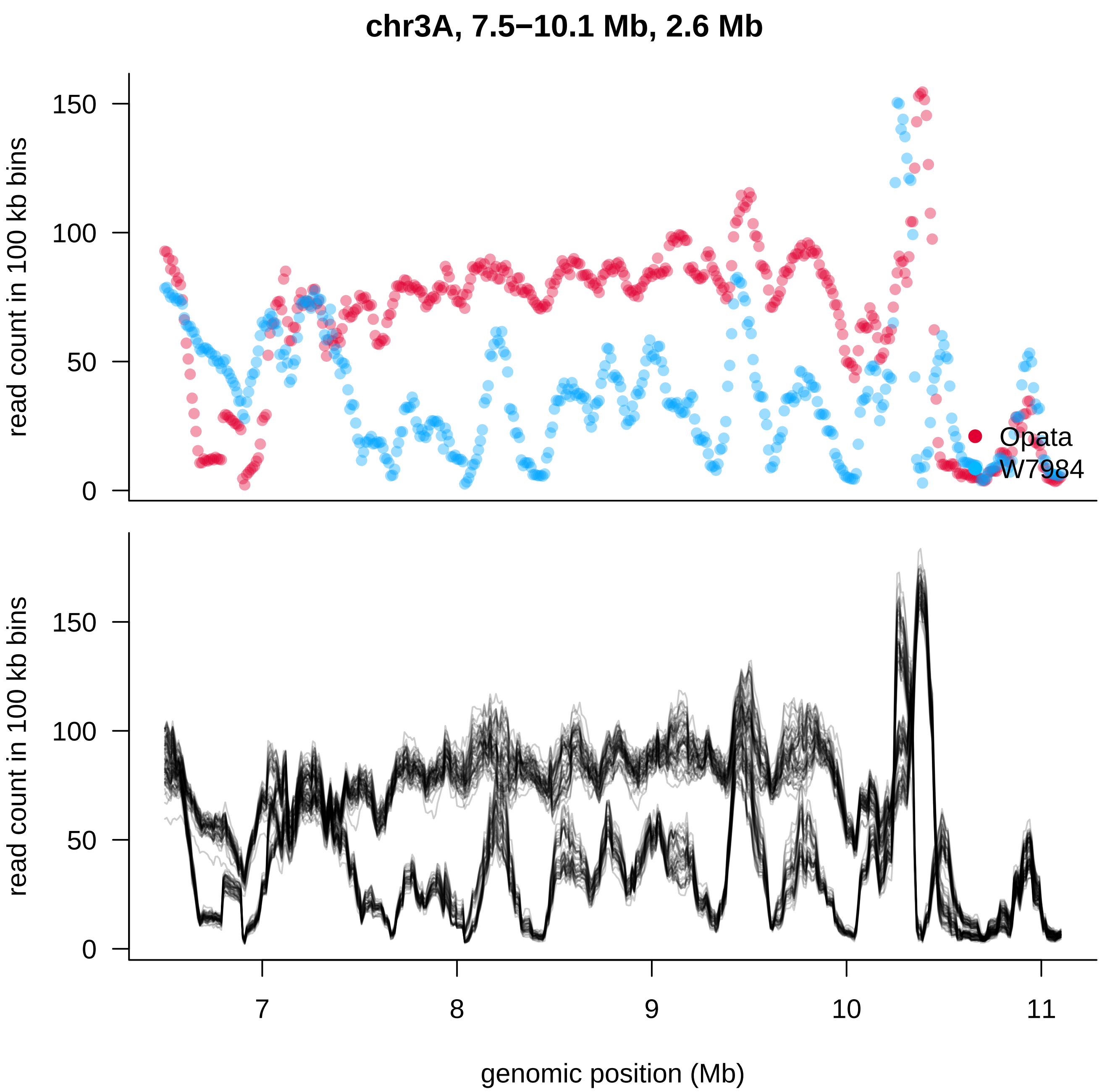

chr3A, 7.5−10.1 Mb, 2.6 Mb
read count in 100 kb bins
150
100
50
0
Opata
W7984
read count in 100 kb bins
150
100
50
0
7
8
9
10
11
genomic position (Mb)

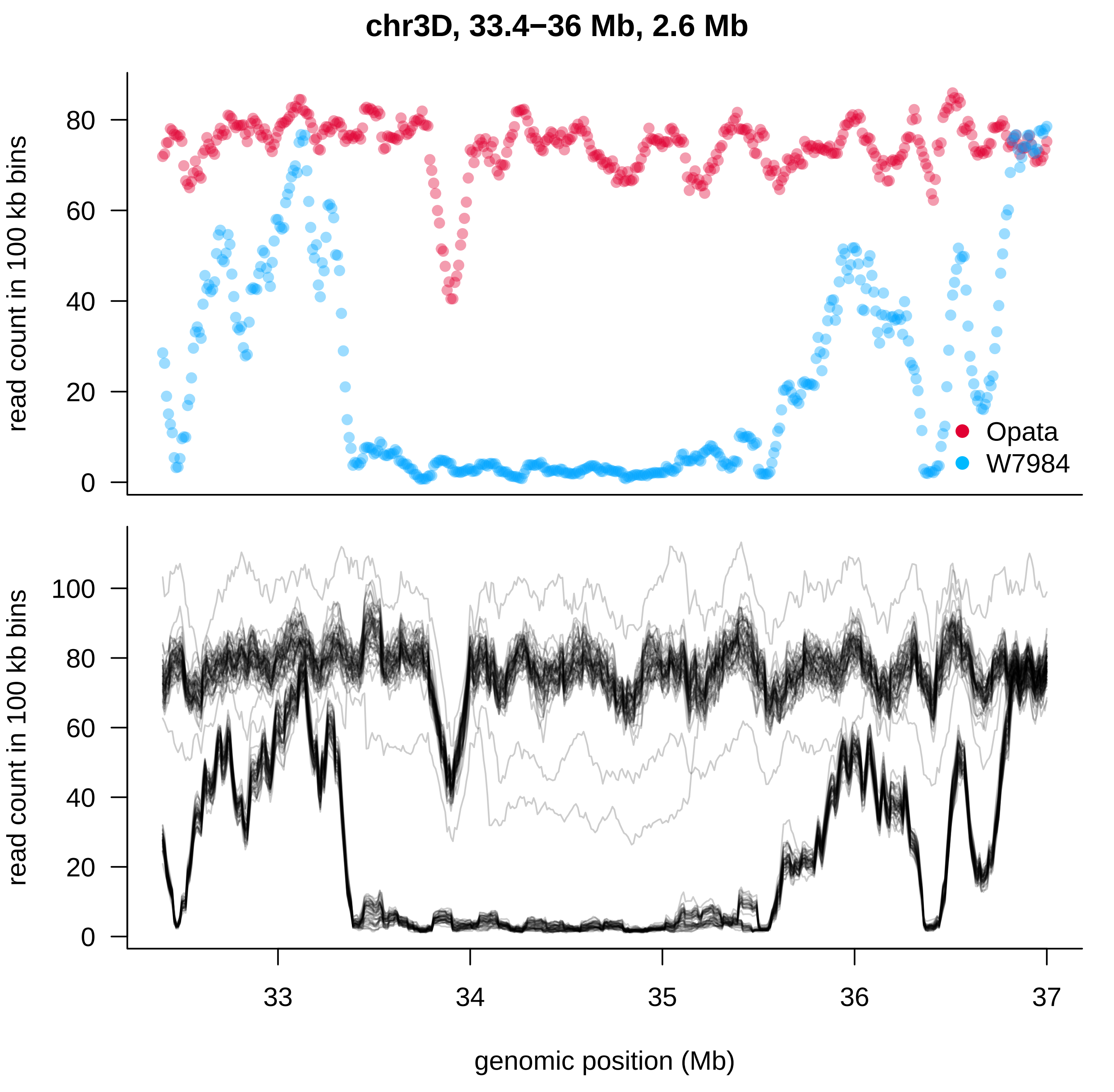
chr3D, 33.4−36 Mb, 2.6 Mb
read count in 100 kb bins
0
20
40
60
80
Opata
W7984
read count in 100 kb bins
0
20
40
60
80
100
33
34
35
36
37
genomic position (Mb)

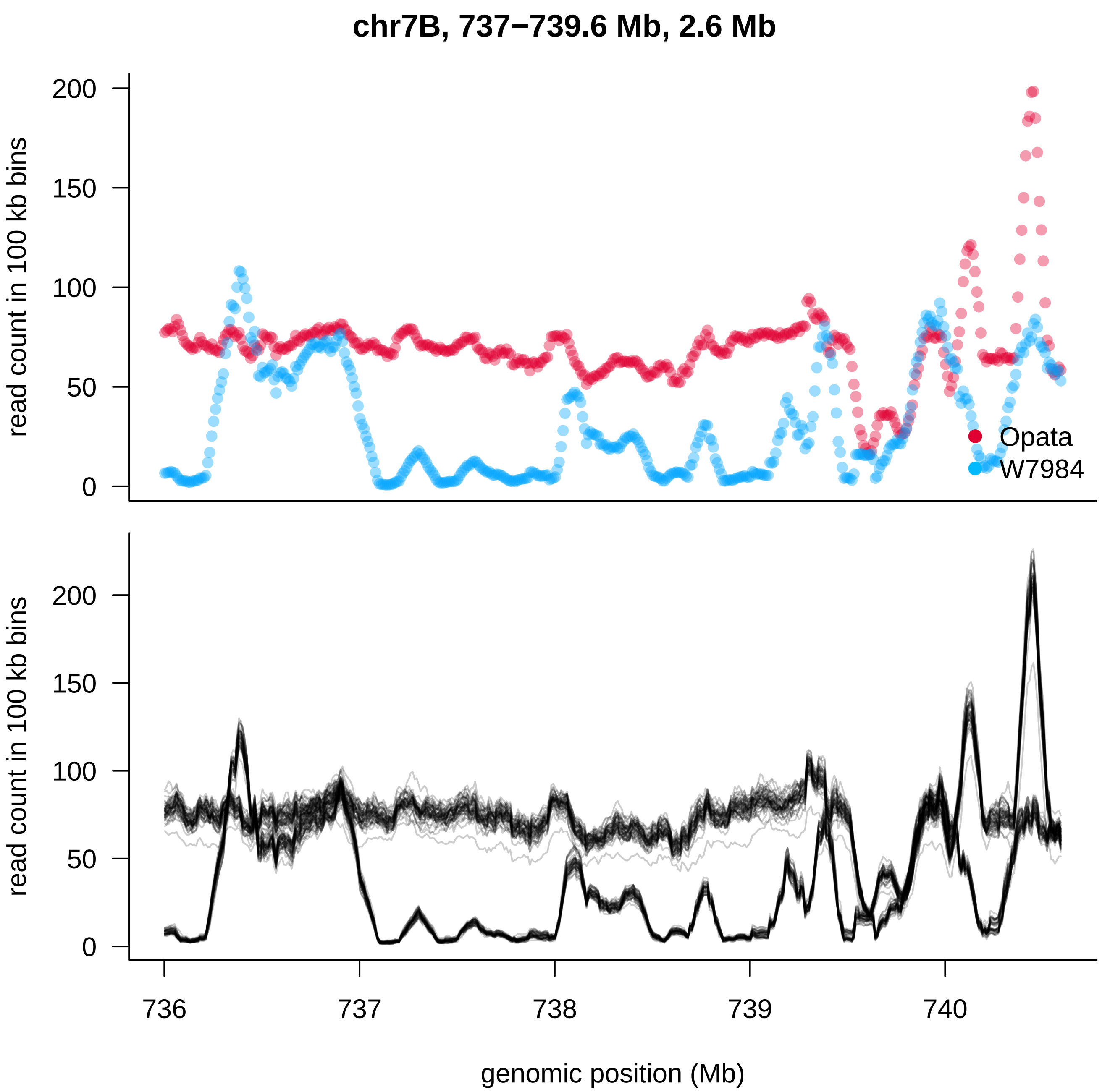

chr7B, 737−739.6 Mb, 2.6 Mb
read count in 100 kb bins
200
150
100
50
0
Opata
W7984
read count in 100 kb bins
200
150
100
50
0
736
737
738
739
740
genomic position (Mb)

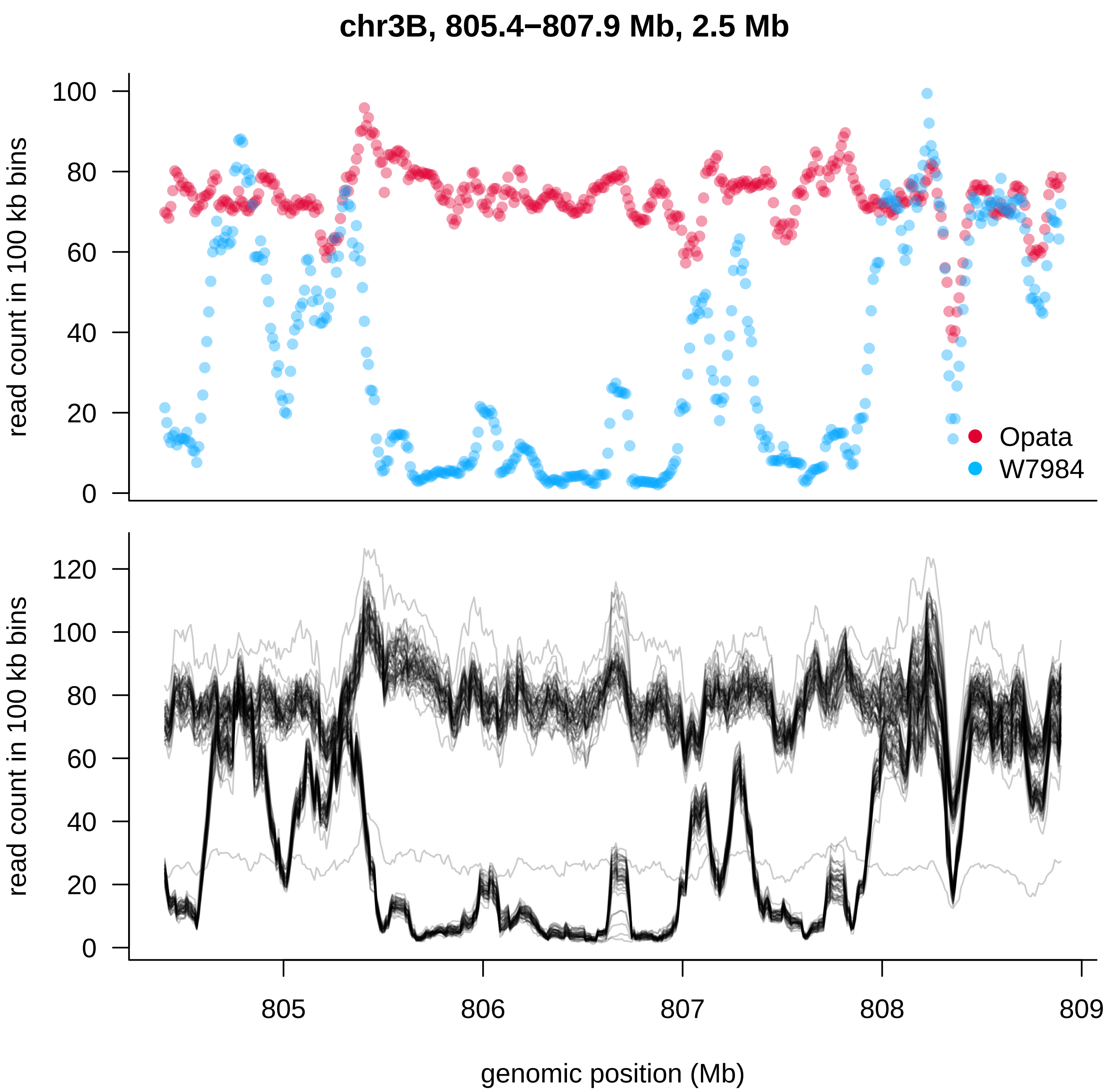
chr3B, 805.4−807.9 Mb, 2.5 Mb
read count in 100 kb bins
0
20
40
60
80
100
Opata
W7984
read count in 100 kb bins
0
20
40
60
80
100
120
805
806
807
808
809
genomic position (Mb)

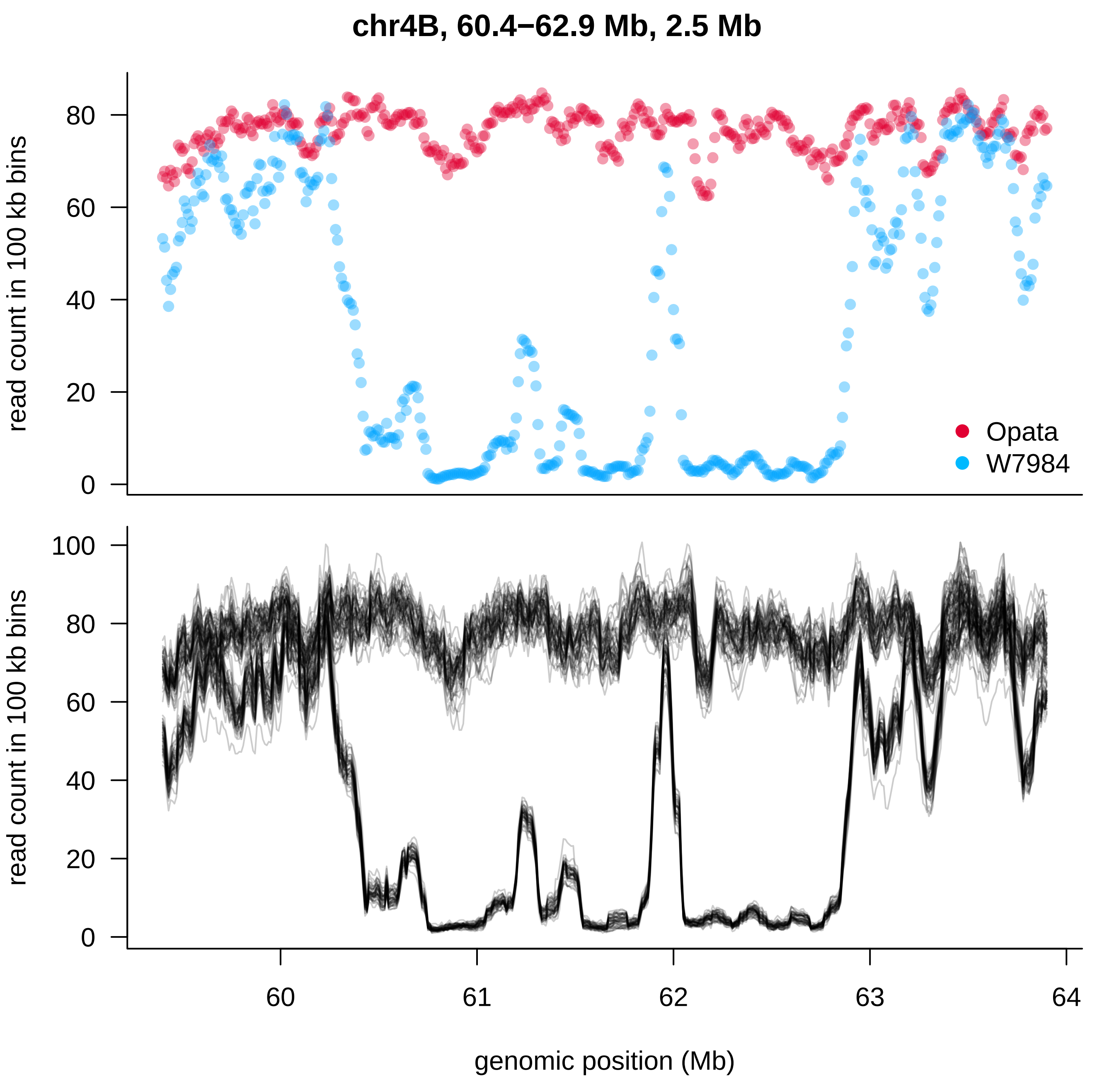
chr4B, 60.4–62.9 Mb, 2.5 Mb
read count in 100 kb bins
0
20
40
60
80
Opata
W7984
read count in 100 kb bins
0
20
40
60
80
100
60
61
62
63
64
genomic position (Mb)

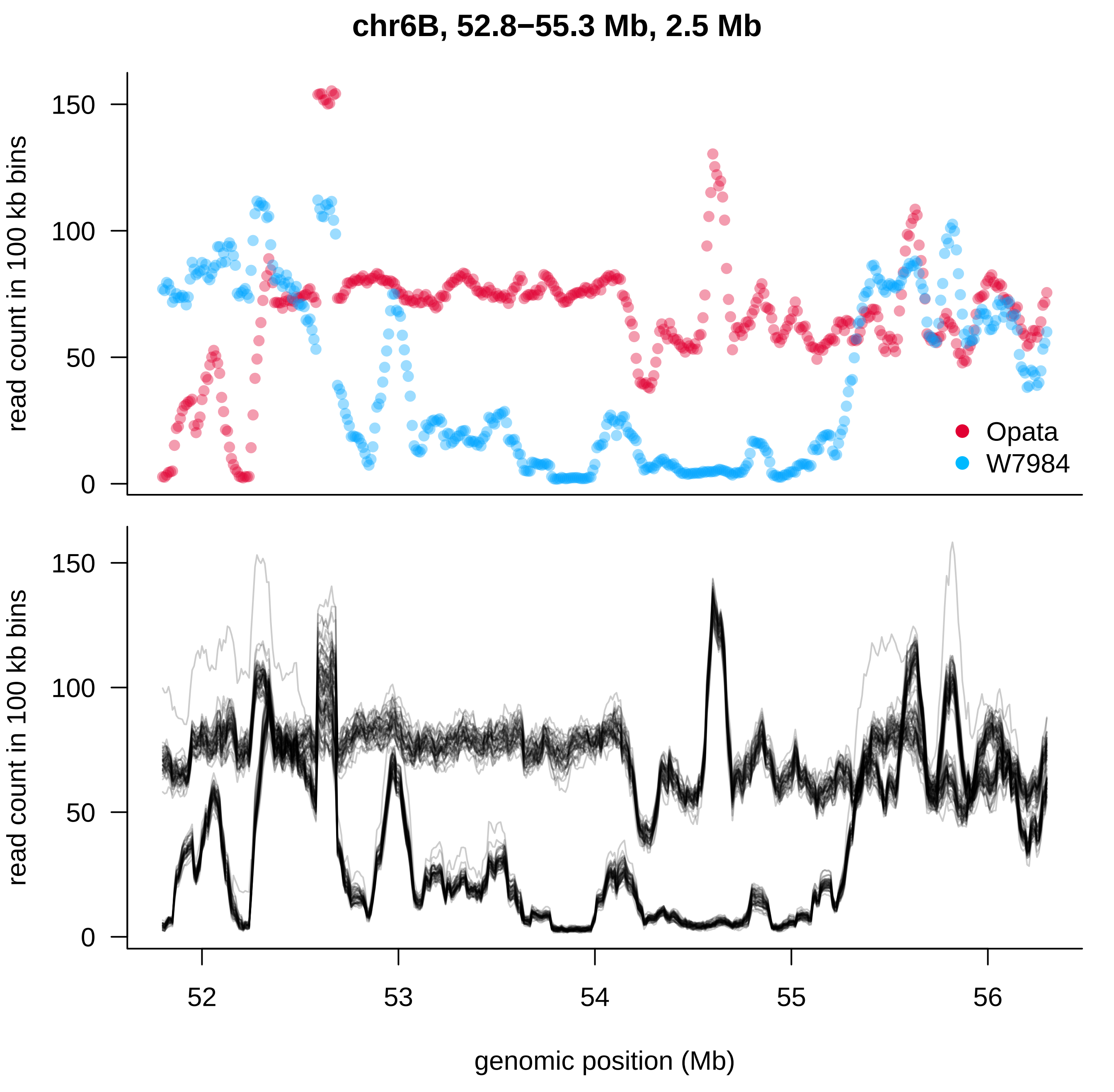
chr6B, 52.8−55.3 Mb, 2.5 Mb
read count in 100 kb bins
150
100
50
0
Opata
W7984
read count in 100 kb bins
150
100
50
0
52
53
54
55
56
genomic position (Mb)

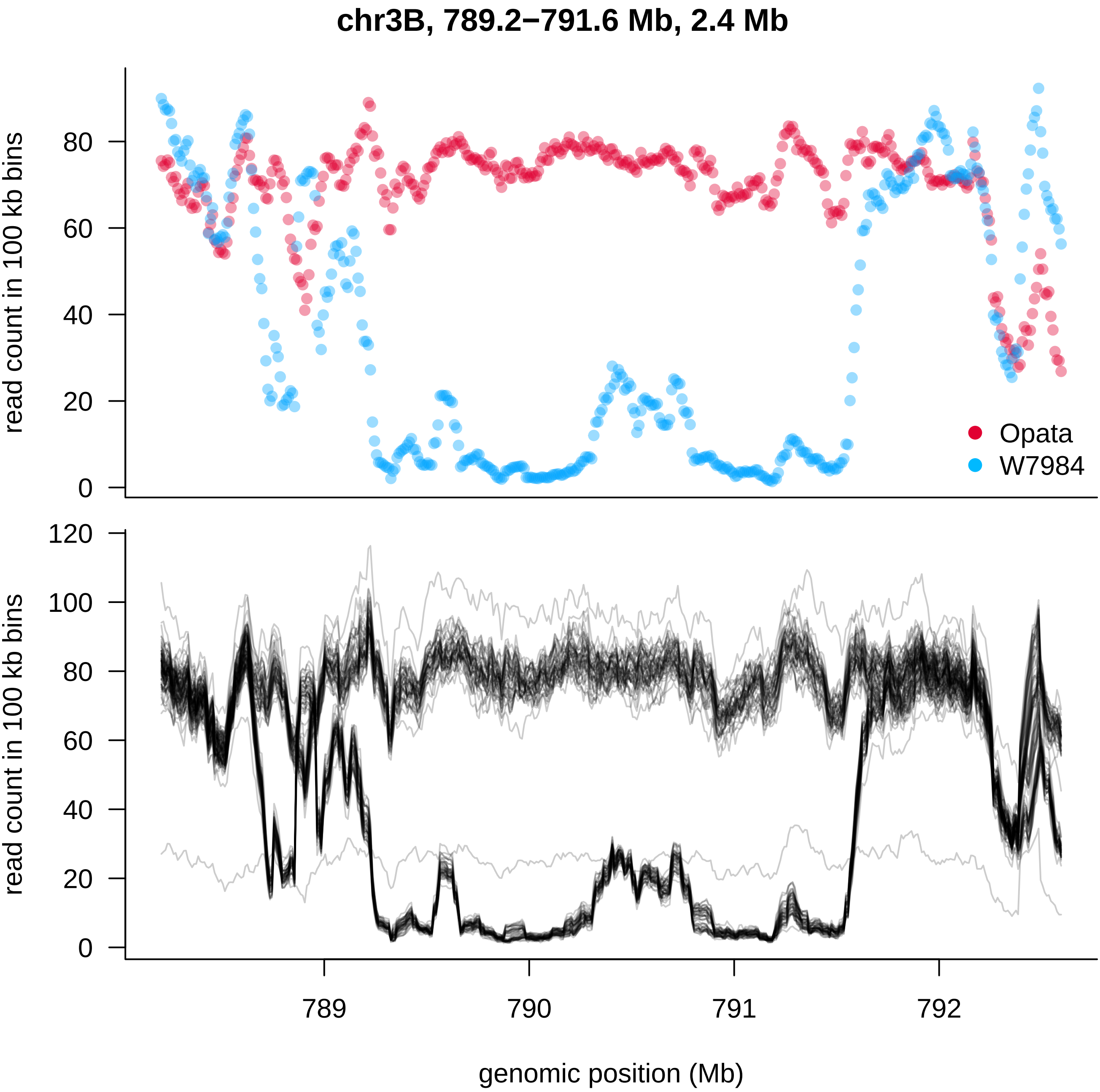
chr3B, 789.2−791.6 Mb, 2.4 Mb
read count in 100 kb bins
0
20
40
60
80
Opata
W7984
read count in 100 kb bins
0
20
40
60
80
100
120
789
790
791
792
genomic position (Mb)

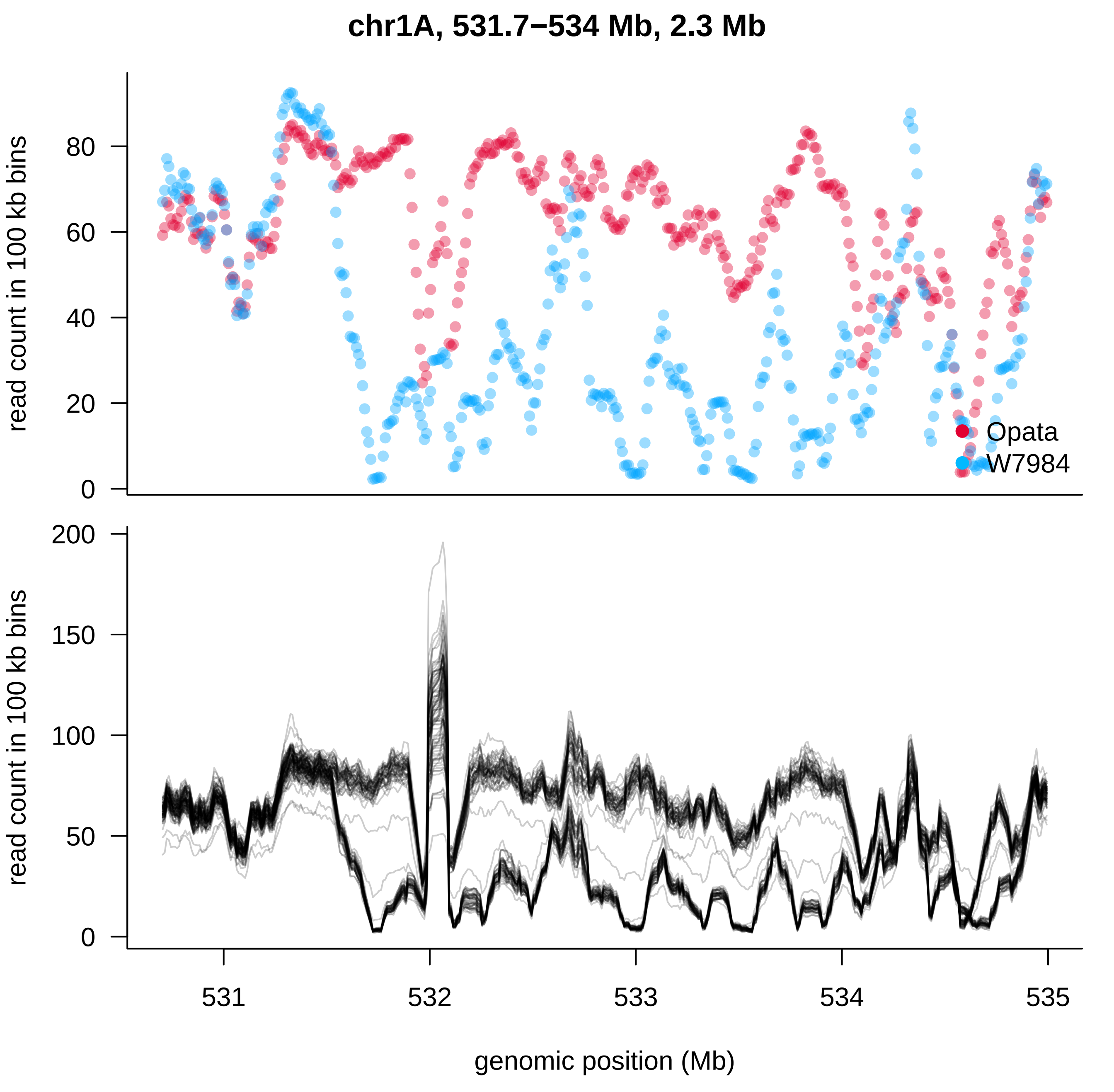

chr1A, 531.7−534 Mb, 2.3 Mb
read count in 100 kb bins
0
20
40
60
80
Opata
W7984
read count in 100 kb bins
0
50
100
150
200
531
532
533
534
535
genomic position (Mb)

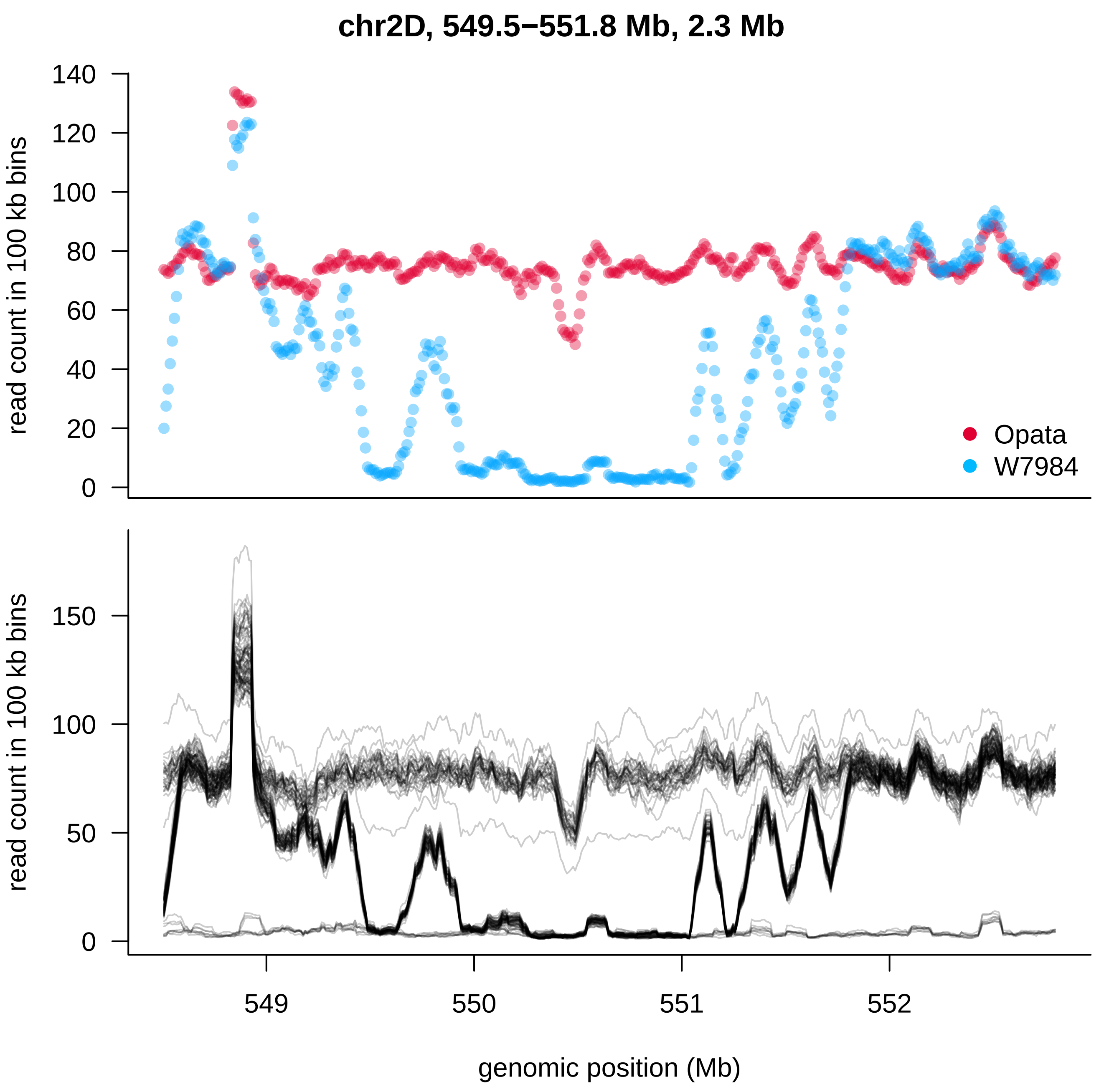
chr2D, 549.5−551.8 Mb, 2.3 Mb
read count in 100 kb bins
0
20
40
60
80
100
120
140
Opata
W7984
read count in 100 kb bins
0
50
100
150
549
550
551
552
genomic position (Mb)

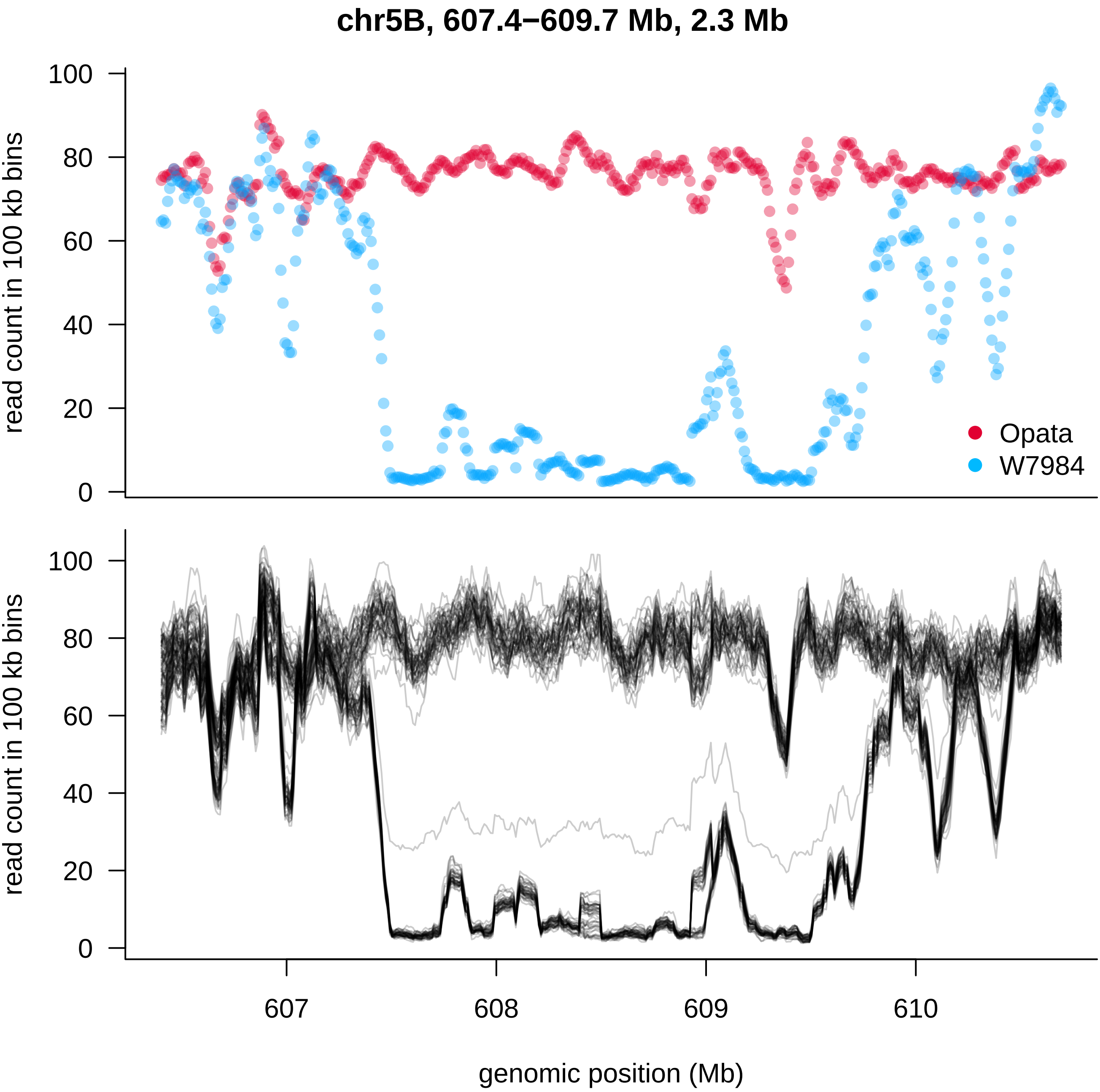
chr5B, 607.4–609.7 Mb, 2.3 Mb
read count in 100 kb bins
0
20
40
60
80
100
Opata
W7984
read count in 100 kb bins
0
20
40
60
80
100
607
608
609
610
genomic position (Mb)

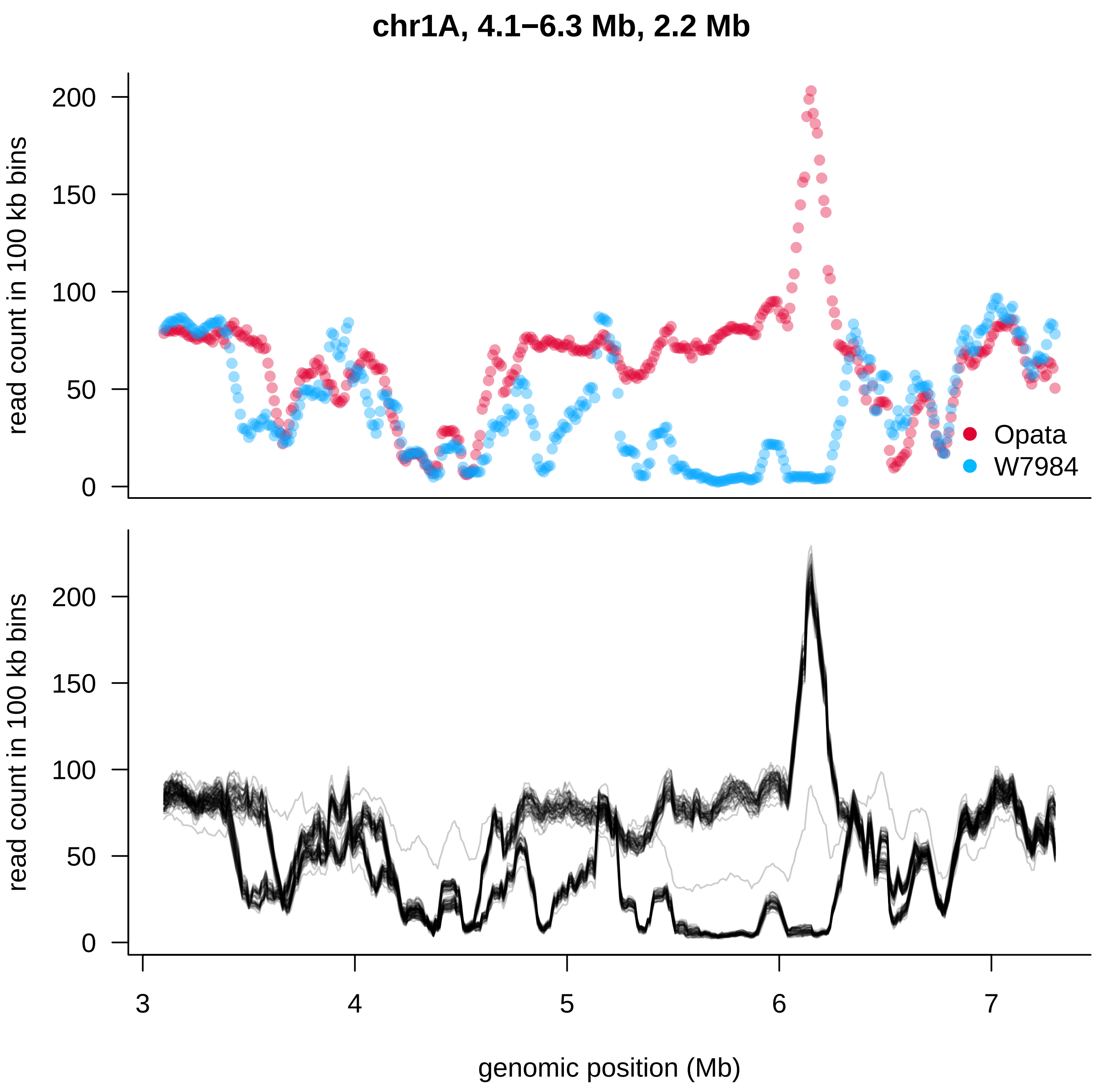

chr1A, 4.1−6.3 Mb, 2.2 Mb
read count in 100 kb bins
0
50
100
150
200
Opata
W7984
read count in 100 kb bins
0
50
100
150
200
3
4
5
6
7
genomic position (Mb)

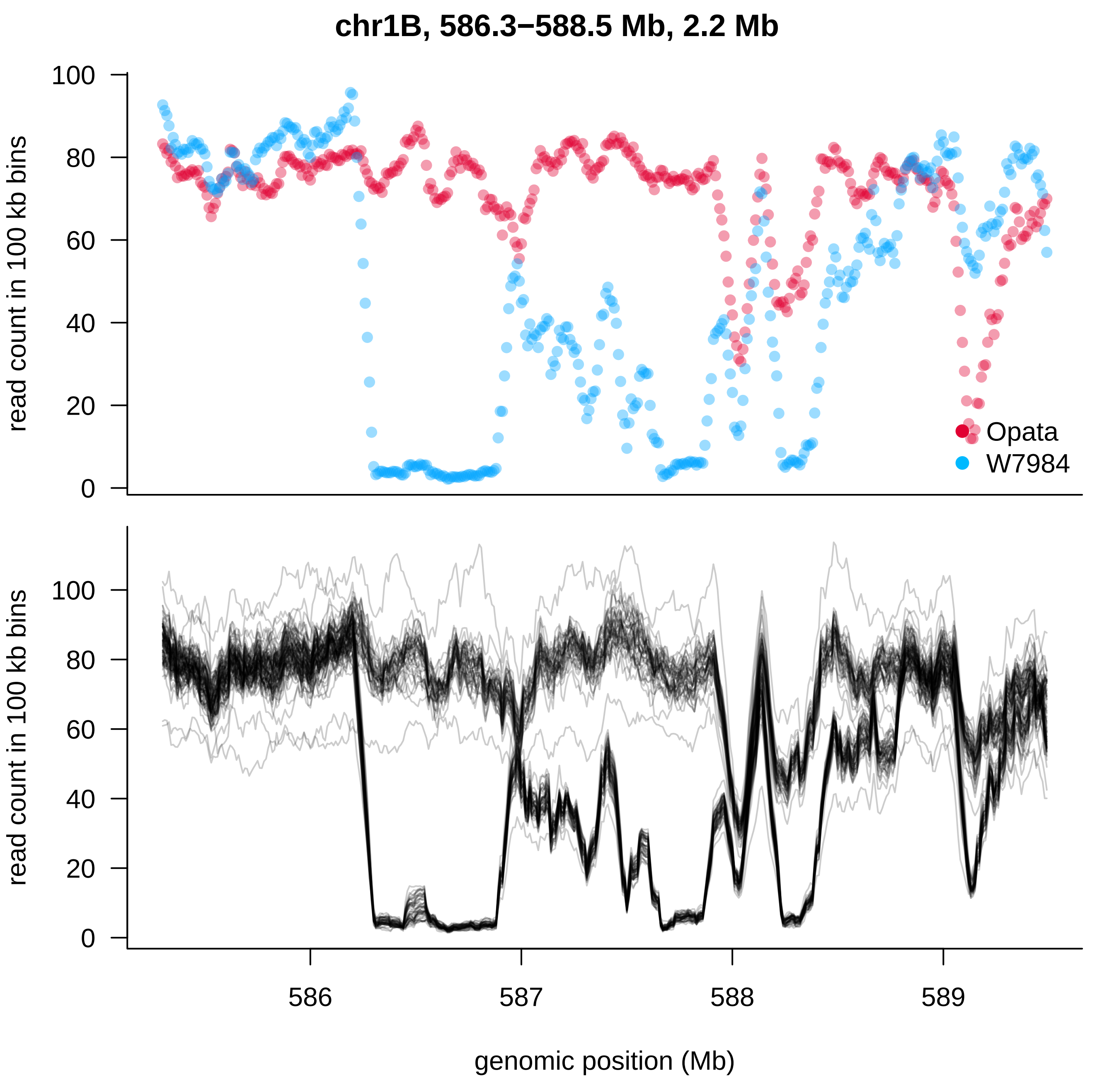

chr1B, 586.3−588.5 Mb, 2.2 Mb
read count in 100 kb bins
0
20
40
60
80
100
Opata
W7984
read count in 100 kb bins
0
20
40
60
80
100
586
587
588
589
genomic position (Mb)

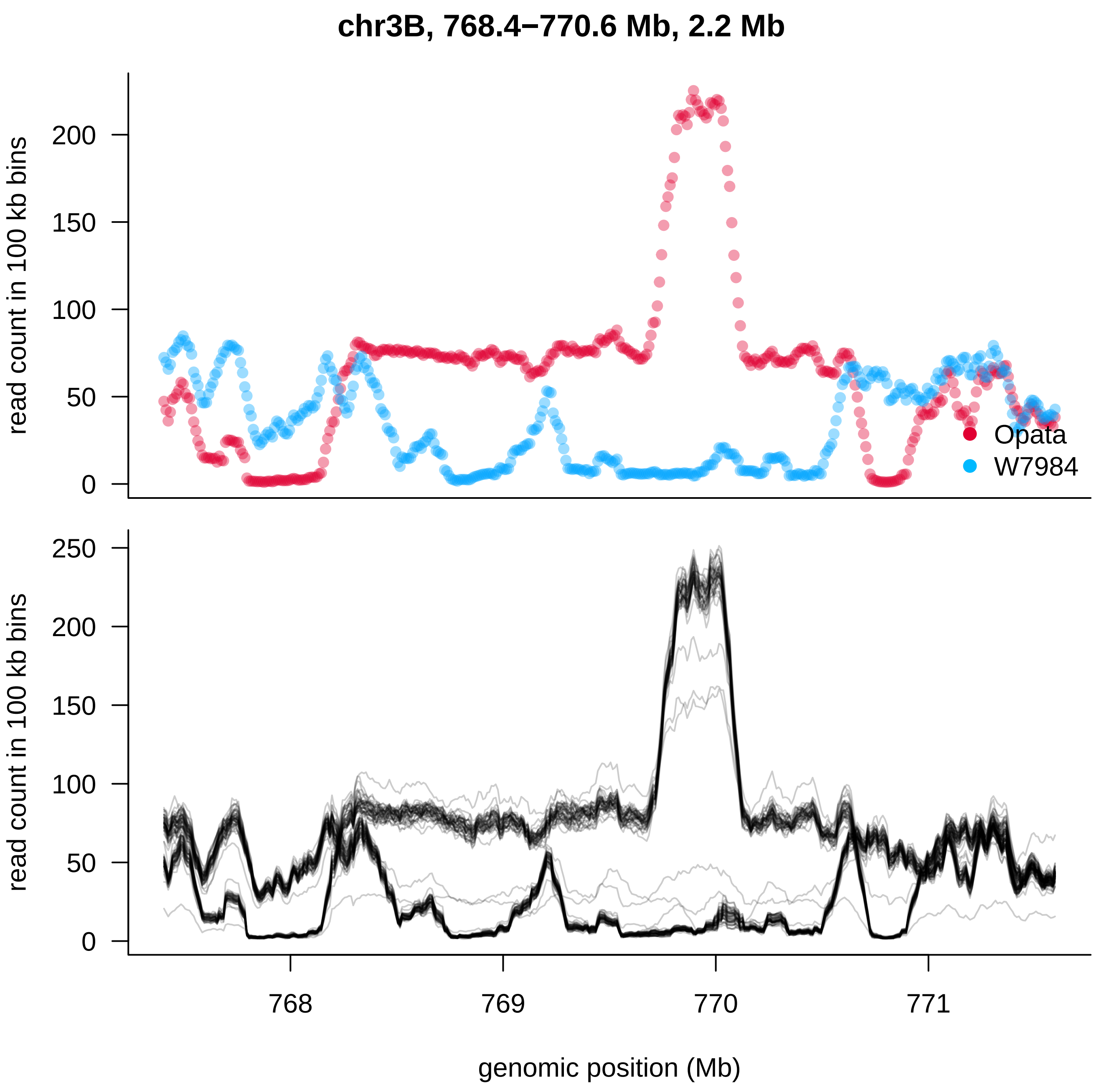

chr3B, 768.4–770.6 Mb, 2.2 Mb
read count in 100 kb bins
200
150
100
50
0
Opata
W7984
read count in 100 kb bins
250
200
150
100
50
0
768
769
770
771
genomic position (Mb)

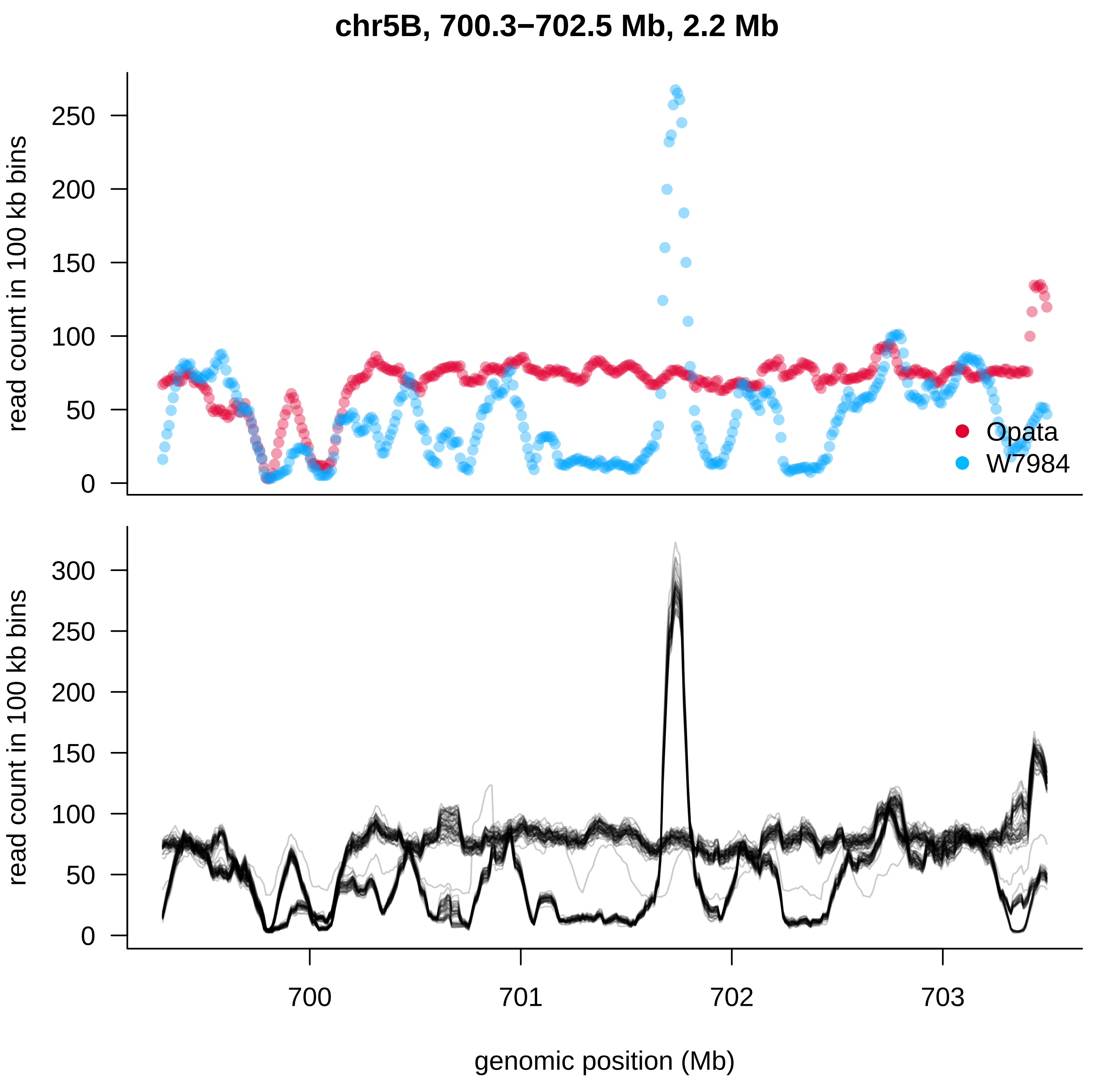

chr5B, 700.3−702.5 Mb, 2.2 Mb
read count in 100 kb bins
0
50
100
150
200
250
Opata
W7984
read count in 100 kb bins
0
50
100
150
200
250
300
700
701
702
703
genomic position (Mb)

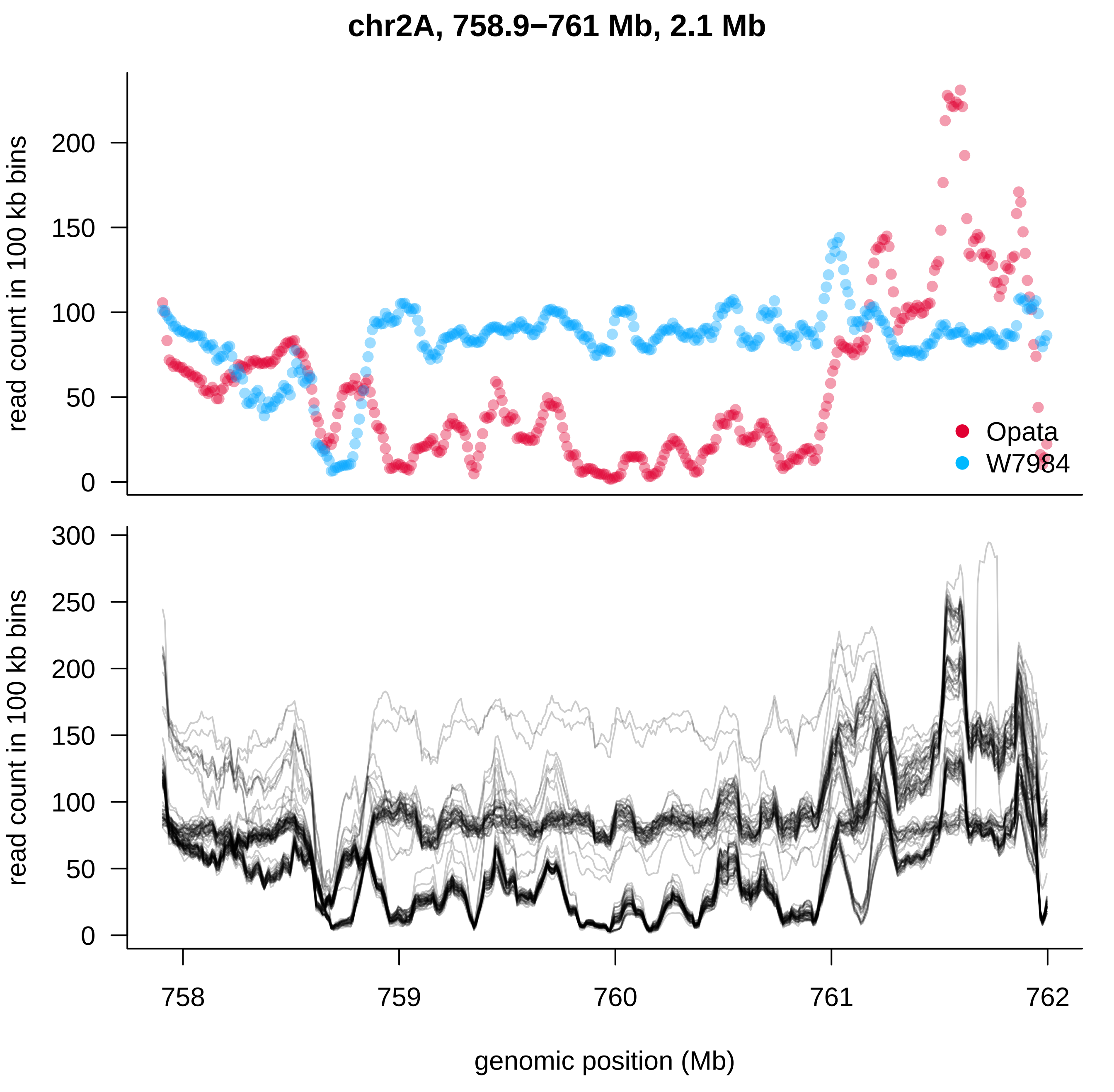
chr2A, 758.9−761 Mb, 2.1 Mb
read count in 100 kb bins
200
150
100
50
0
Opata
W7984
read count in 100 kb bins
300
250
200
150
100
50
0
758
759
760
761
762
genomic position (Mb)

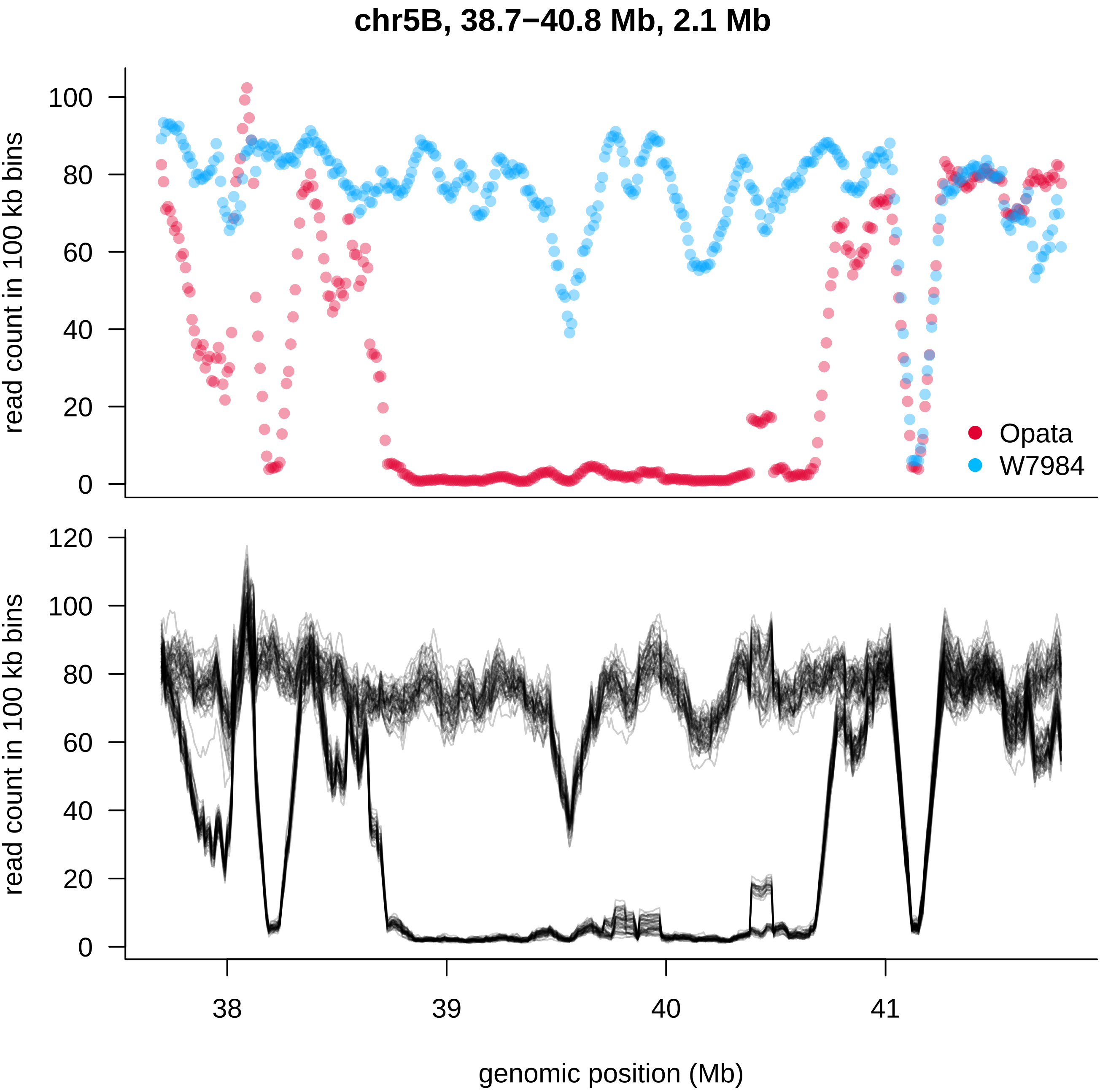
chr5B, 38.7−40.8 Mb, 2.1 Mb
read count in 100 kb bins
100
80
60
40
20
0
Opata
W7984
read count in 100 kb bins
120
100
80
60
40
20
0
38
39
40
41
genomic position (Mb)

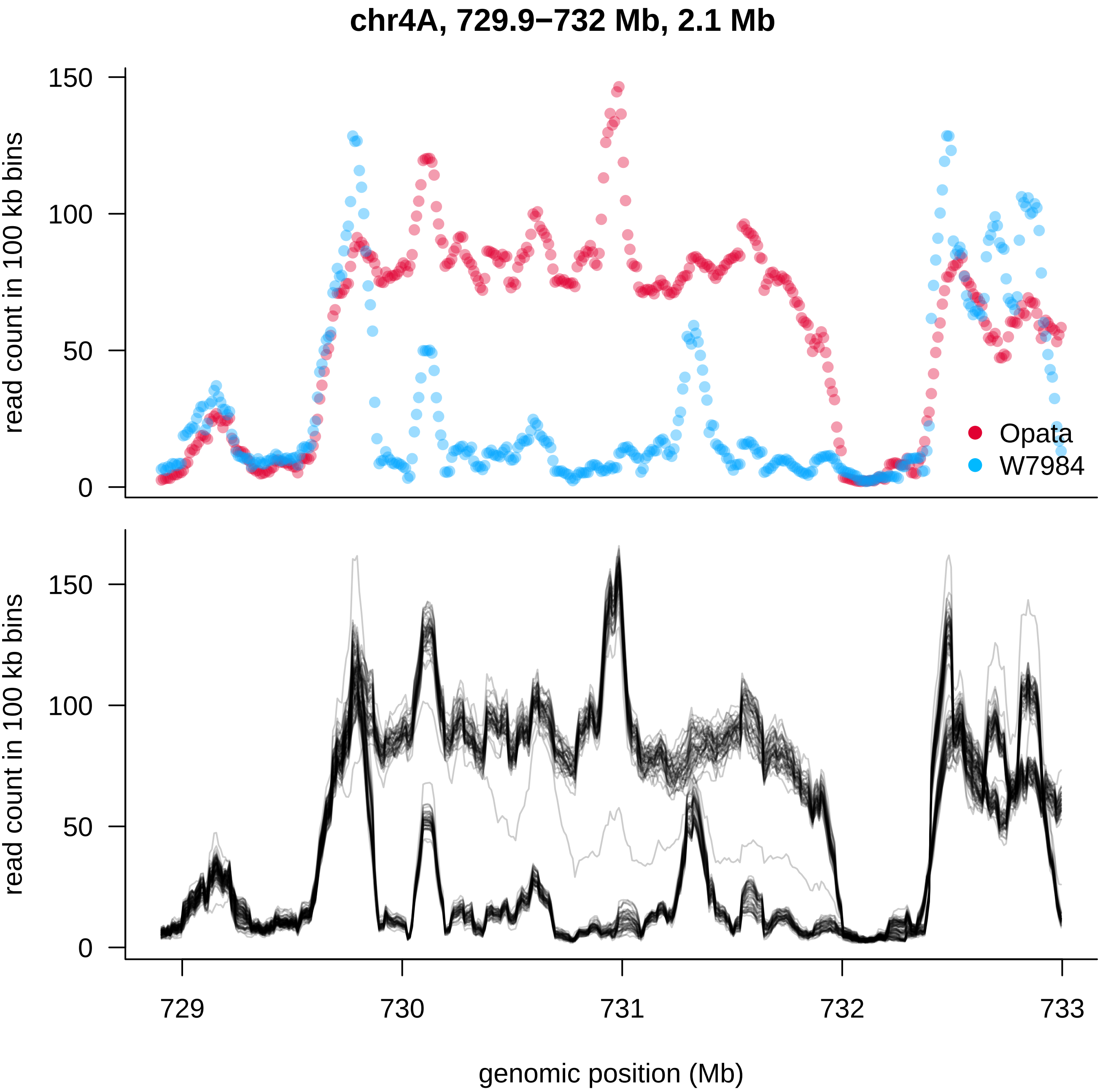

chr4A, 729.9−732 Mb, 2.1 Mb
read count in 100 kb bins
150
100
50
0
Opata
W7984
read count in 100 kb bins
150
100
50
0
729
730
731
732
733
genomic position (Mb)

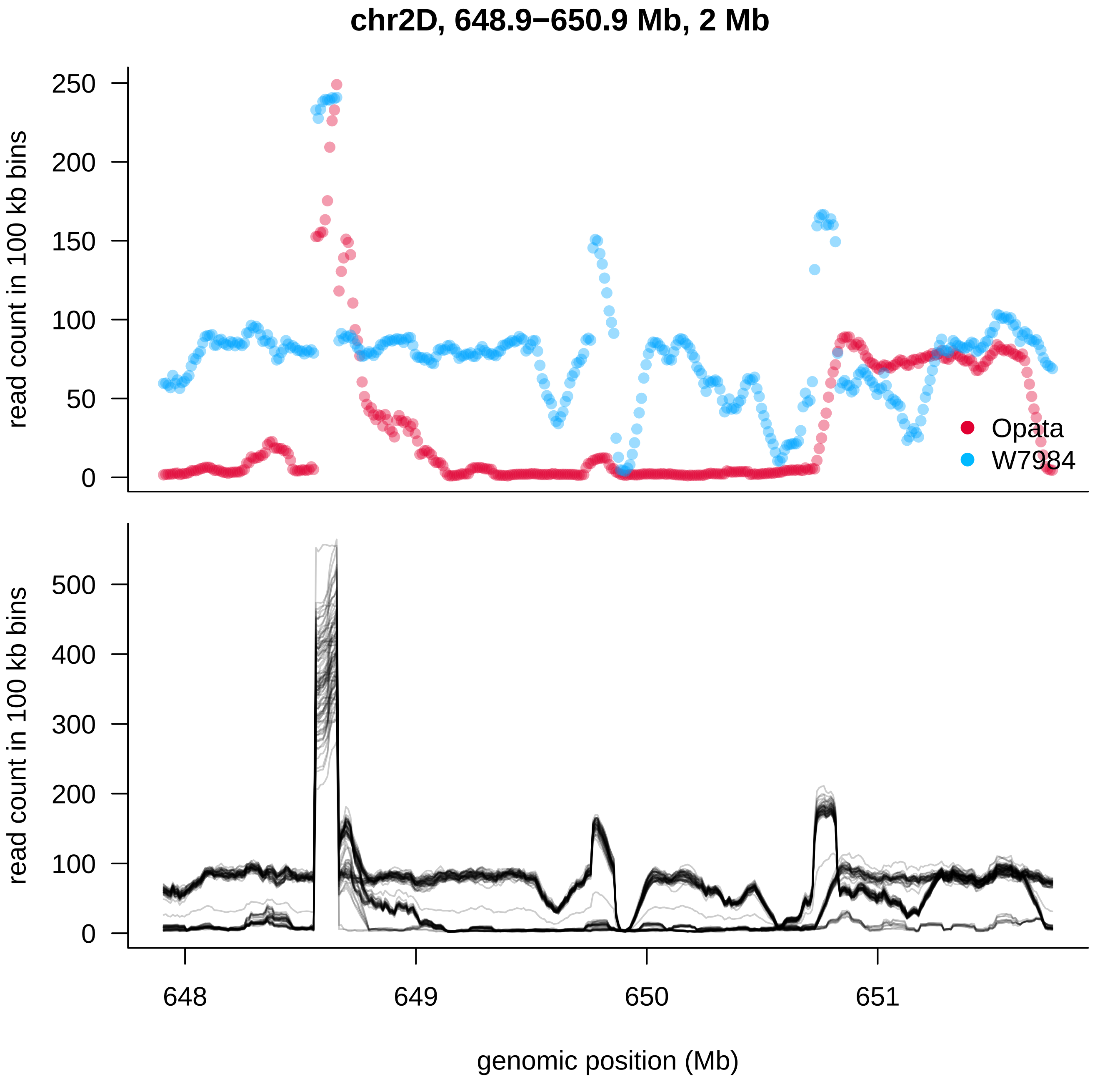

chr2D, 648.9−650.9 Mb, 2 Mb
read count in 100 kb bins
250
200
150
100
50
0
Opata
W7984
read count in 100 kb bins
500
400
300
200
100
0
648
649
650
651
genomic position (Mb)

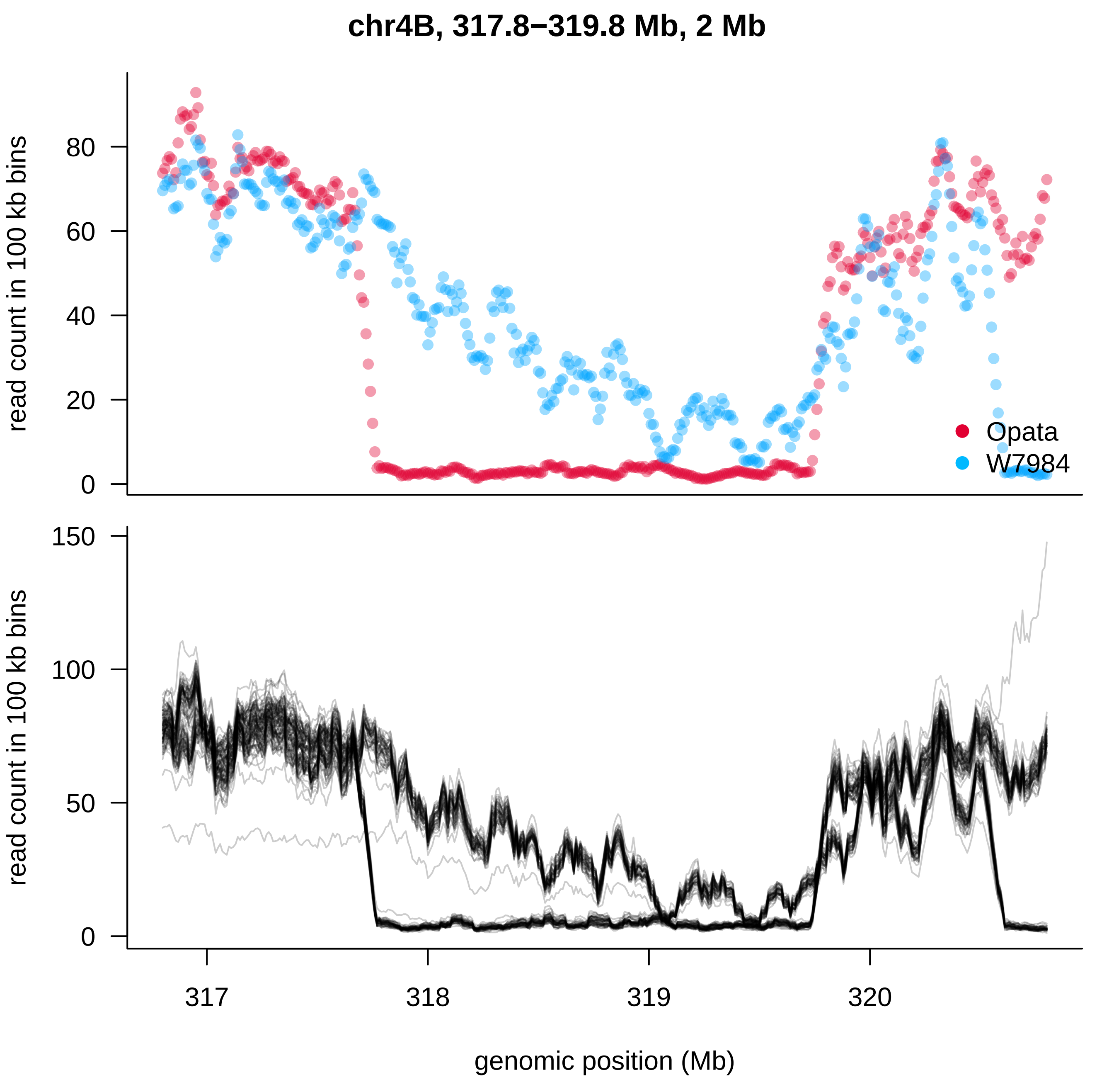
chr4B, 317.8−319.8 Mb, 2 Mb
read count in 100 kb bins
0
20
40
60
80
Opata
W7984
read count in 100 kb bins
0
50
100
150
317
318
319
320
genomic position (Mb)

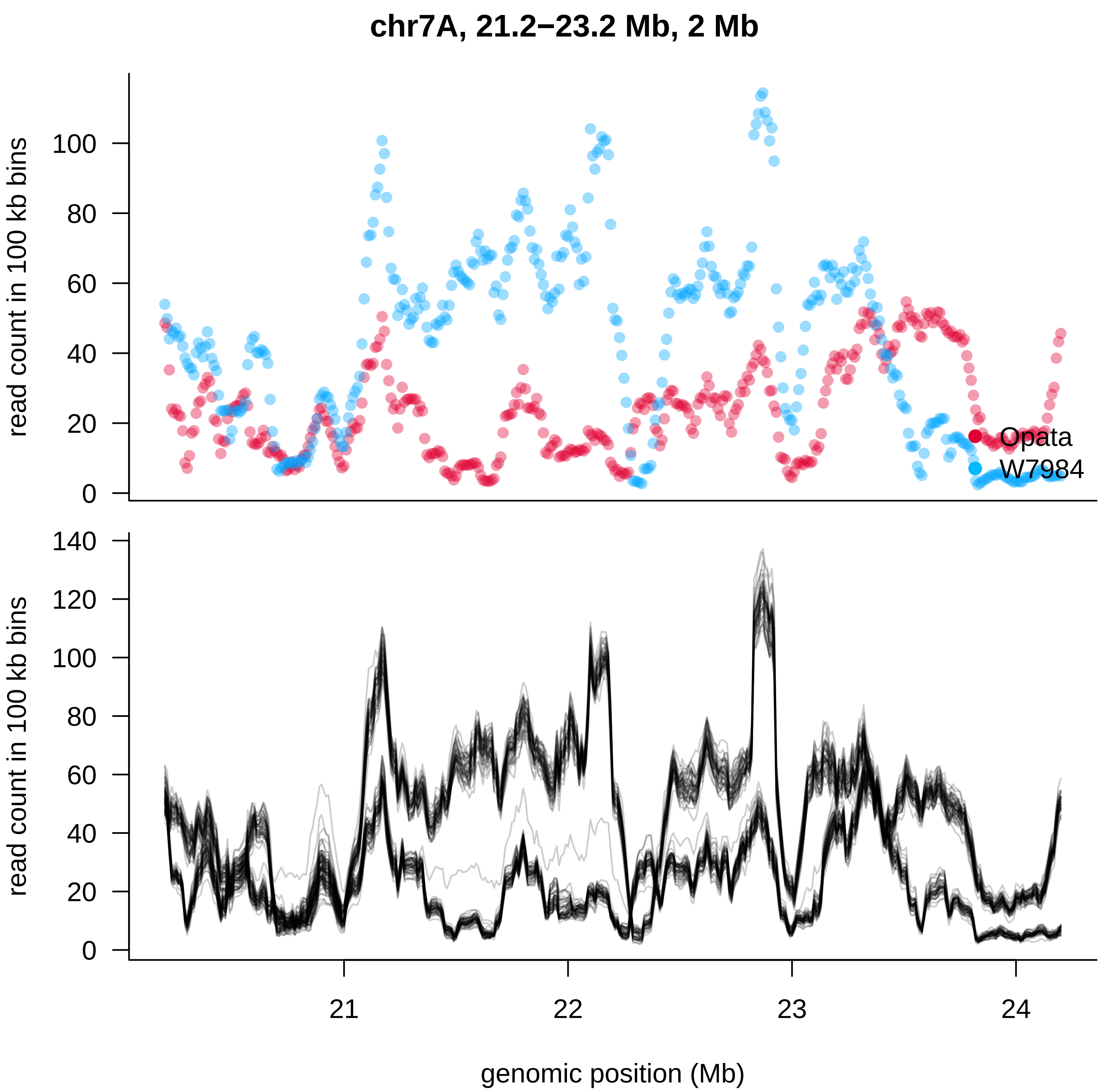
chr7A, 21.2−23.2 Mb, 2 Mb
read count in 100 kb bins
0
20
40
60
80
100
Opata
W7984
read count in 100 kb bins
0
20
40
60
80
100
120
140
21
22
23
24
genomic position (Mb)

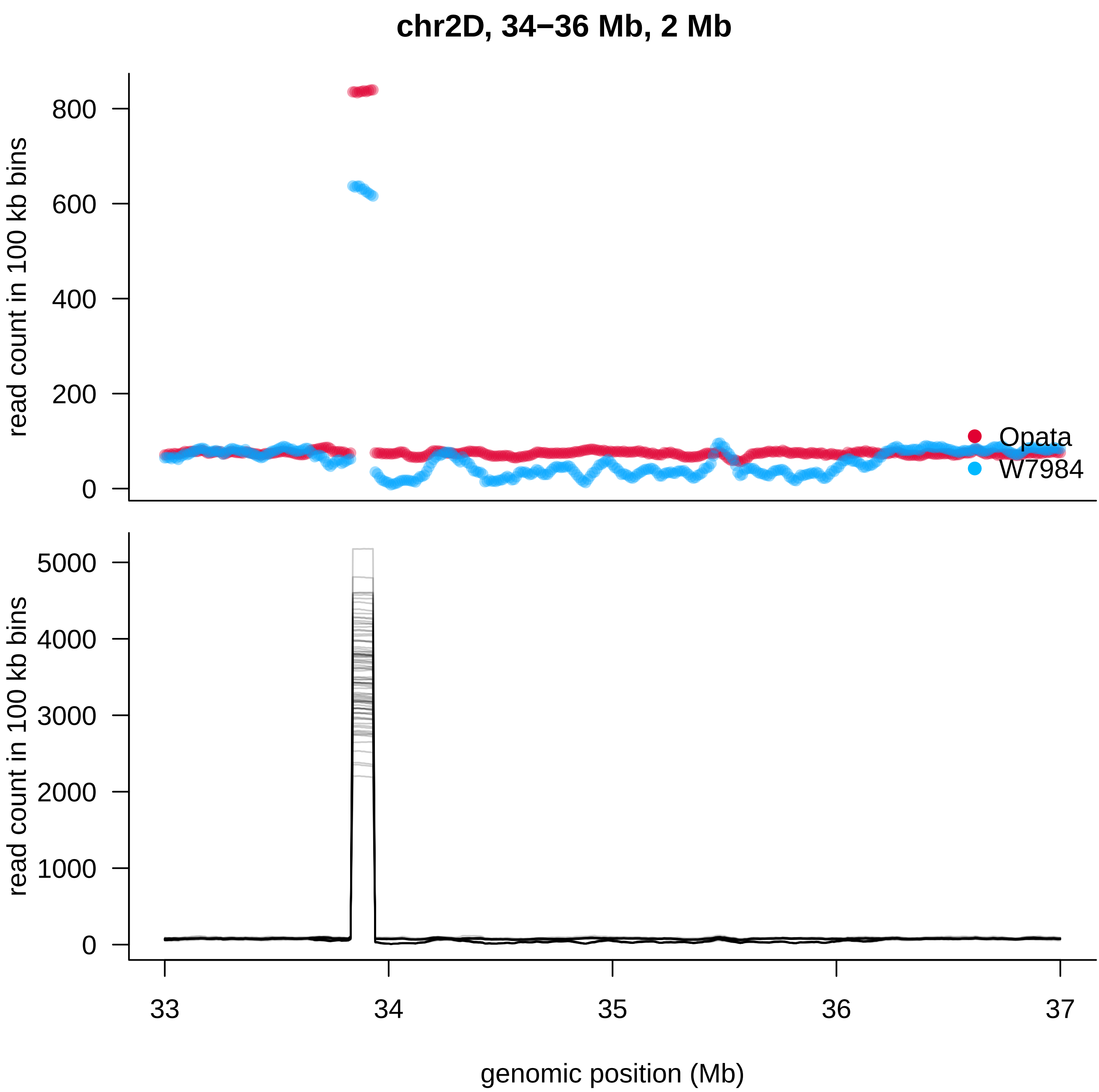
chr2D, 34−36 Mb, 2 Mb
read count in 100 kb bins
800
600
400
200
0
Opata
W7984
read count in 100 kb bins
5000
4000
3000
2000
1000
0
33
34
35
36
37
genomic position (Mb)

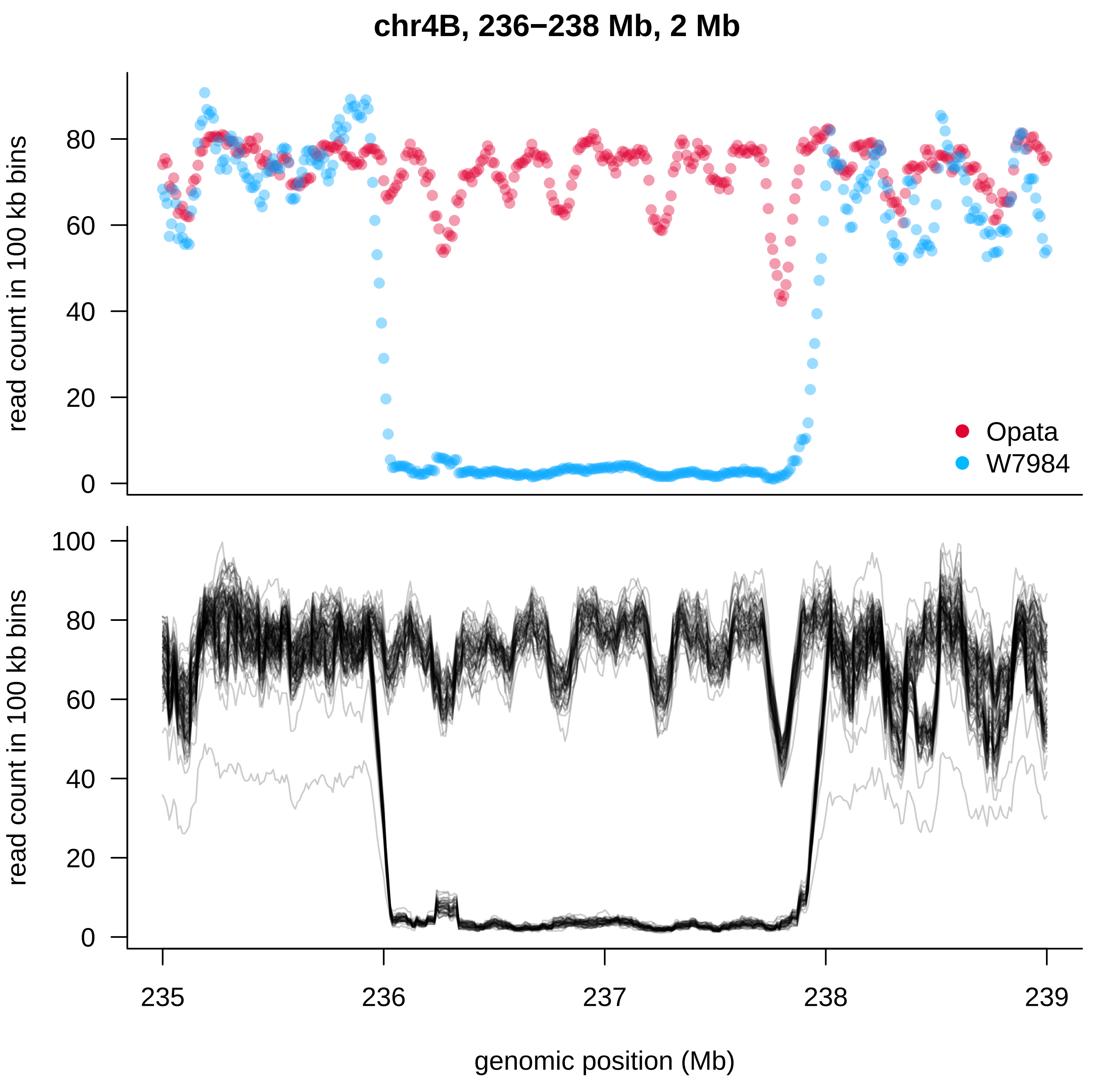

chr4B, 236−238 Mb, 2 Mb
read count in 100 kb bins
0
20
40
60
80
Opata
W7984
read count in 100 kb bins
0
20
40
60
80
100
235
236
237
238
239
genomic position (Mb)

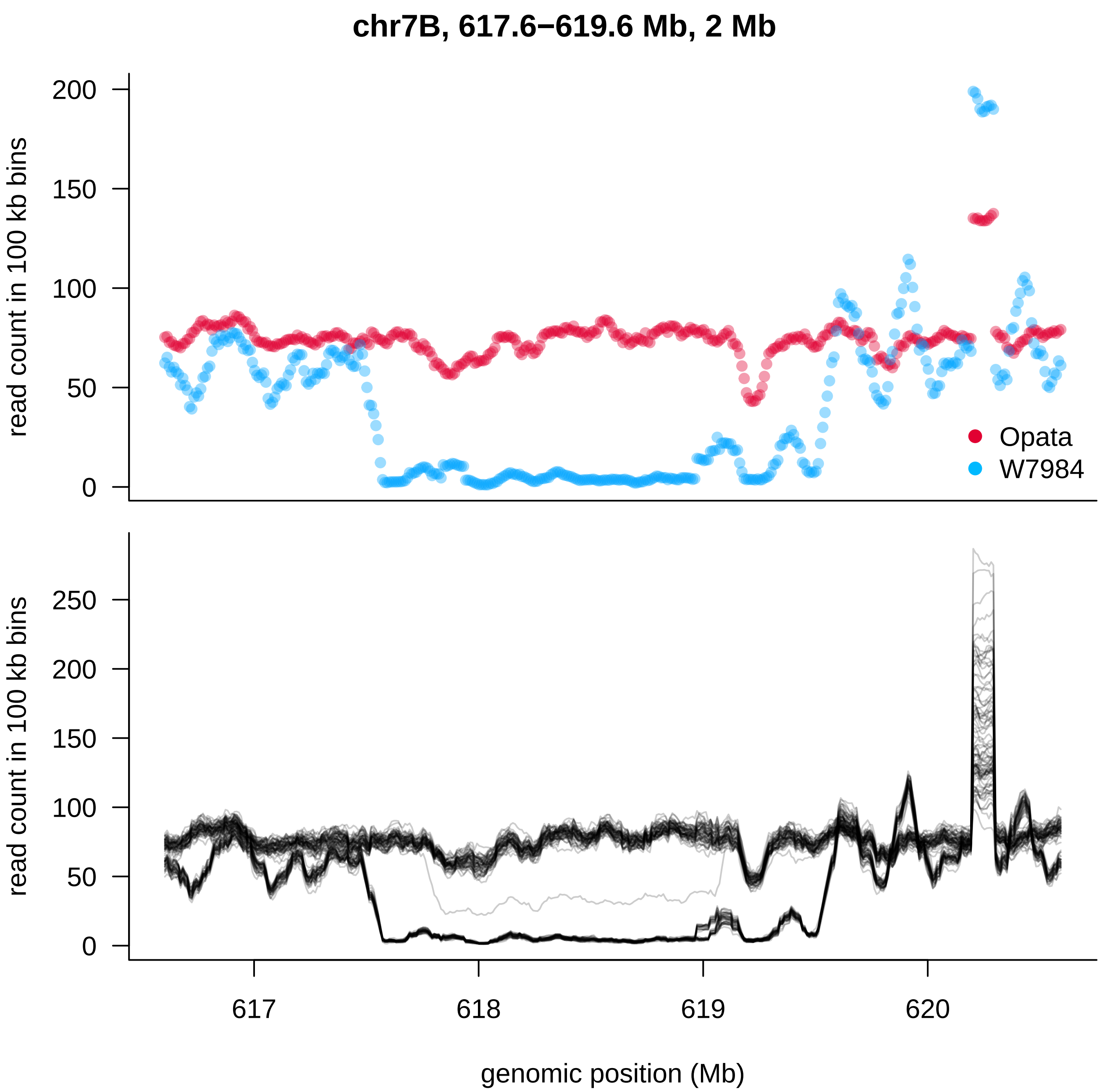
chr7B, 617.6−619.6 Mb, 2 Mb
read count in 100 kb bins
200
150
100
50
0
Opata
W7984
read count in 100 kb bins
250
200
150
100
50
0
617
618
619
620
genomic position (Mb)
