# Supplementary material for: Dense genotyping-by-sequencing linkage maps of two Synthetic W7984×Opata reference populations provide insights into wheat structural diversity
Source: Sci Rep. 2019 Feb 11;9:1793. doi: 10.1038/s41598-018-38111-3 (PMC6370774; doi:10.1038/s41598-018-38111-3)
Supplement: Supplementary file 15 — Dataset S14 [file 41598_2018_38111_MOESM15_ESM.docx]

#!/usr/bin/perl

# BY: Juan Gutierrez-Gonzalez

# USED FOR: Takes hamming distance output from Mascher et al., 2013a, and a genetic map. Places each imputed marker in the most likely position in the map.

# USAGE perl add.imputedmks.2.map.pl -m linkage.map.file -i hamming.file

# format of the linkage map file format of the hamming file (That is, output from Mascher et al., 2013a, with the genotype calls removed)

# 1A 0 chr1A_1145442 chr1A_1158042 16 0 0 2 chr1A_1145442,chr1A_1238074

# 1A 6.734047969 chr1A_3793383 chr1A_1159442 16 0 0 2 chr1A_1145442,chr1A_1238074

# 1A 7.872710958 chr1A_4804732 chr1A_1159478 16 0 0 2 chr1A_1145442,chr1A_1238074

# 1A 8.904535851 chr1A_4812400 chr1A_3838416 16 0 0 1 chr1A_3793383

# 1A 8.904535851 chr1A_6027915 chr1A_9567537 38 0 3 5 chr1A_10132856,chr1A_12502888,chr1A_12822017,chr1A_12822041,chr1A_12822066

#

# ---------------------------------------------------------------

# *** TERMS ***

# ...............................................................

#

# User acknowledges that the program is a research tool still

# under the development and that it is being supplied "as is".

# User is responsible for throughly testing program and ensure

# results are functionally corrected.

#

# Developer(s) shall not be held liable for any liability nor for

# any direct, indirect or consequential damages with respect

# to any claim by RECIPIENT or any third party on account of

# or arising from in use of the programs

#

# .......................................................................

use File::Basename;

use Getopt::Long;

my $PROGRAM = basename($0);

use strict;

use warnings;

sub usage { print "\nUsage: $PROGRAM -m linkage map -i hammingfile [-o output]\n"; }

if ($#ARGV < 2) { warn "number of commandline args: $#ARGV\n"; &usage; exit 1; }

my @Options =

(

"m=s" => \(my $mapfile),

"i=s" => \(my $hammingfile),

"o=s" => \(my $output),

);

if (!&GetOptions(@Options) ){ &usage; exit 1; }

if (!$mapfile ){ warn "\nMust specify a file containing a linkage map\n"; &usage; exit 1; }

if (!$hammingfile ){ warn "\nMust specify a file containing hamming distantces as Mascher et al,2013 \n"; &usage; exit 1; }

if (!$output ){ $output = &defaultName( $mapfile ); $output .= ".withimputedmks"; }

print "\n==========================================================================\n";

print " *** $PROGRAM running ***";

print "\n==========================================================================\n\n";

my @chrorder = qw(1A 2A 3A 4A 5A 6A 7A 1B 2B 3B 4B 5B 6B 7B 1D 2D 3D 4D 5D 6D 7D);

open (OUT, "> $output" ) or print "\nFail open $output \n" and die;

open (MAPFILE, "< $mapfile" ) or print "\nFail open $mapfile \n" and die;

open (HAMMINGFILE, "< $hammingfile" ) or print "\nFail open $hammingfile \n" and die;

my %hashLG;

while (<MAPFILE>){

chomp; my @mapfileline = split( /\t/, $_);

my $chr = $mapfileline[0]; $chr =~ s/\s+//g;

my $pos = $mapfileline[1]; $pos =~ s/\s+//g;

my $mk = $mapfileline[2]; $mk =~ s/\s+//g;

if (!exists $hashLG{$chr}{$pos}){

@{$hashLG{$chr}{$pos}} = ();

push @{$hashLG{$chr}{$pos}}, $mk;

}else{

push @{$hashLG{$chr}{$pos}}, $mk;

}

}

close (MAPFILE);

my %hashsimilarmks;

while (<HAMMINGFILE>){

chomp; my @hammingfileline = split( /\t/, $_);

my $mkid = $hammingfileline[0];

my @similarmks = split (/\,/, $hammingfileline[5]);

foreach my $i (@similarmks){

push @{$hashsimilarmks{$mkid}}, $i;

}

foreach my $n (@chrorder) {

foreach my $key1 (sort hashValueAscending (keys %{$hashLG{$n}}) ){

if(isSubset(\@{$hashsimilarmks{$mkid}},\@{$hashLG{$n}{$key1}})){

push @{$hashLG{$n}{$key1}}, $mkid;

}

}

}

}

close (HAMMINGFILE);

foreach my $nn (@chrorder) {

foreach my $k1 (sort {$a <=> $b} (keys %{$hashLG{$nn}}) ){

foreach my $k2 (@{$hashLG{$nn}{$k1}}) {

print OUT "$nn\t$k1\t$k2\n";

}

}

}close (OUT);

print " *** $PROGRAM ended ***\n";

sub hashValueAscending {$a cmp $b};

sub defaultName {

my ($sub_input) = @_;

my @sub_inline = split ( /\//, $sub_input );

my $sub_filename = $sub_inline[scalar(@sub_inline)-1];

return $sub_filename;

}

sub isSubset {

my ($littleSet, $bigSet) = @_;

my %hash;

undef @hash{@$littleSet};

delete @hash{@$bigSet};

return !%hash;

}
